# The *AvrPm3-Pm3* effector-NLR interactions control both race-specific resistance and host-specificity of cereal mildews on wheat

**DOI:** 10.1038/s41467-019-10274-1

**Published:** 2019-05-23

**Authors:** Salim Bourras, Lukas Kunz, Minfeng Xue, Coraline Rosalie Praz, Marion Claudia Müller, Carol Kälin, Michael Schläfli, Patrick Ackermann, Simon Flückiger, Francis Parlange, Fabrizio Menardo, Luisa Katharina Schaefer, Roi Ben-David, Stefan Roffler, Simone Oberhaensli, Victoria Widrig, Stefan Lindner, Jonatan Isaksson, Thomas Wicker, Dazhao Yu, Beat Keller

**Affiliations:** 1 Department of Plant and Microbial Biology, University of Zurich, 8008 Zurich, Switzerland; 2 Department of Forest Mycology and Plant Pathology, Division of Plant Pathology, Swedish University of Agricultural Sciences, 750 07 Uppsala, Sweden; 3 Institute of Plant Protection and Soil Science, Hubei Academy of Agricultural Sciences, Wuhan, 430064 China; 4 Ministry of Agriculture Key Laboratory of Integrated Pest Management in Crops in Central China, Wuhan, 430064 China; 5 College of Life Science, Wuhan University, Wuhan, 430072 China; 6 Institute of Plant Science, ARO-Volcani Center, 50250 Bet Dagan, Israel

**Keywords:** Effectors in plant pathology, Biotic

## Abstract

The wheat *Pm3* resistance gene against the powdery mildew pathogen occurs as an allelic series encoding functionally different immune receptors which induce resistance upon recognition of isolate-specific avirulence (AVR) effectors from the pathogen. Here, we describe the identification of five effector proteins from the mildew pathogens of wheat, rye, and the wild grass *Dactylis glomerata*, specifically recognized by the PM3B, PM3C and PM3D receptors. Together with the earlier identified AVRPM3^A2/F2^, the recognized AVRs of PM3B/C, (AVRPM3^B2/C2^), and PM3D (AVRPM3^D3^) belong to a large group of proteins with low sequence homology but predicted structural similarities. *AvrPm3*^*b2/c2*^ and *AvrPm3*^*d3*^ are conserved in all tested isolates of wheat and rye mildew, and non-host infection assays demonstrate that *Pm3b*, *Pm3c*, and *Pm3d* are also restricting the growth of rye mildew on wheat. Furthermore, divergent AVR homologues from non-adapted rye and *Dactylis* mildews are recognized by PM3B, PM3C, or PM3D, demonstrating their involvement in host specificity.

## Abstract (web summary)

The wheat Pm3 immune receptors confer resistance against powdery mildew by recognizing isolate-specific avirulence (AVR) effectors of the pathogen. Here, the authors identify and characterize two new *AVR* genes and demonstrate that Pm3 receptors are determinants of host-specificity for grass mildews.

## Introduction

Fungi are highly adaptive, widespread organisms and ubiquitous pathogens of animals and plants responsible for population declines and pandemics in natural and agricultural ecosystems^1–5^. In wheat, there have been two recent fungal pandemics caused by the emergence of wheat blast^6^ and stem rust (Ug99^7^). The *Sr35* resistance (*R*) gene encodes a nucleotide binding-leucine rich repeat (NLR) immune receptor that upon recognition of the avirulence protein AVRSr35 confers near-immunity to Ug99^8,9^. This case demonstrates that effector-NLR interactions conferring immunity following the simplest model of *Avr-R* gene-for-gene interactions^10,11^ provide an important leverage for achieving rapid control of emerging disease pandemics in crops.

Powdery mildews are agronomically important fungal pathogens of large numbers of wild and cultivated species. In cereals, the powdery mildew disease is caused by a single species, *Blumeria graminis*. It occurs in different, highly host-specific forms called *formae speciales*^12,13^. For example, *B. graminis* f. sp. *tritici* (*B.g. tritici*) exclusively grows on wheat, whereas *B. graminis* f. sp. *secalis* (*B.g. secalis*) and *B. graminis* f. sp. *dactylidis* (*B.g. dactylidis*) specifically infect rye and the wild grass species *Dactylis glomerata*, respectively^13^. Powdery mildews are obligate biotrophs, growing and reproducing exclusively on living host tissue. This requires suppression of host immune responses which is possibly achieved through the massive secretion of small virulence proteins called effectors^14,15^. Recent studies in barley powdery mildew have established the role of some effectors as virulence factors^16–21^. Effector proteins can be specifically recognised by NLR plant immune receptors whose activation confer resistance to the invading pathogen. This isolate-specific resistance is often linked to a rapid cell death response at sites of attempted infection, designated as the hypersensitive response (HR)^22,23^. Effectors recognised by R-proteins are called avirulence (AVR) proteins and are often found to be polymorphic in different isolates of the pathogenic fungus^15^.

Allelic series of NLR-type receptors have been described in several plant species. The largest allelic series of resistance genes in wheat is formed by the *Pm3* gene against powdery mildew. Up to date, 17 functional alleles (*Pm3a-g; Pm3k*-*Pm3t*) have been cloned and functionally validated^24,25^, and they confer complete resistance against distinctly different sets of *B.g. tritici* races^26^. Compared to other well studied allelic series of *R* genes such as the flax rust (*Melampsora lini*) resistance gene *L* or the *B.g. hordei* resistance gene *Mla*, the *Pm3* series stands out due to its very high (>97%) sequence identity on the protein level^22,25,27^.

The molecular basis of the functional diversity of the closely related *Pm3* alleles is largely unknown. Up to date, the only known avirulence gene for a *Pm3* allele is *AvrPm3*^*a2/f2*^, an effector gene recognised by the *Pm3a* and *Pm3f* alleles^28^. Despite high sequence conservation among the *Pm3* NLRs, no allele other than *Pm3a* and *Pm3f* can recognise *AvrPm3*^*a2/f2*^ or any of its closest gene family relatives or natural haplotype variants^28,29^. Furthermore Bourras and collaborators^28^ provided evidence that the *Pm3* resistance follows a genetically complex gene-for-gene model^11^, which involves a pathogen encoded suppressor of avirulence (*SvrPm3*^*a1/f1*^) acting on several *AvrPm3-Pm3* specificities.

In this study, we describe the identification of the *AvrPm3*^*b2/c2*^ and *AvrPm3*^*d3*^ genes and provide molecular evidence that specificity of the *Pm3* NLRs is based on recognition of highly sequence diverse, but structurally similar effectors. We also demonstrate that the same effector genes are conserved in the non-adapted rye and *Dactylis* powdery mildews, thus demonstrating that the *Pm3* NLRs, apart from their race-specific resistance function, are potent determinants of host-specificity for grass mildews. Therefore, we propose that the *AvrPm3-Pm3* interactions provide a unique model system to understand how NLRs can contribute to both host and non-host resistance.

## Results

### Identification of *AvrPm3* candidates by effector benchmarking

In a first approach to identify *AvrPm3* candidate genes we established a new assay based on the hypothesis that effector proteins acting as AVR factors are likely to share structural similarities, sequence polymorphism, and similar expression patterns at the haustorial stage. At the time this assay was designed, four mildew *Avrs* were cloned: *AvrPm3*^*a2/f2*^, *AvrPm2* from *B.g. tritici*^28,30^ and *Avr*_*a1*_, *Avr*_*a13*_ from *B.g. hordei*^31^. They are all encoded by typical effector proteins, ranging in size from 118 (AVR_A1_) to 130 aa (AVRPM3^A2/F2^), with a predicted N terminal signal peptide, two conserved cysteine residues (except AVR_A13_ which contains only one), and high expression at the haustorial stage^28,30,31^. Therefore, we hypothesised that mildew AVRs correspond to short proteins of ca. 120 aa on average, encoded by highly expressed candidate effector genes, that can be differentiated in mildew isolates with contrasting virulence patterns based on sequence polymorphism or gene expression levels.

We scanned the genomes of the three wheat powdery mildew reference isolates, Bgt_96224, Bgt_94202 and Bgt_JIW2, which are polymorphic for several *AvrPm3* specificities^28,32^, and systematically classified and scored 580 effectors based on sequence polymorphism (SNPs and deletions), presence of a functional signal peptide, cysteine content, native protein size, and gene expression levels (Supplementary Note 1, Supplementary Fig. 1). This resulted in an “effector benchmarking” list with effector scores for candidate *AvrPm3* genes ranging from −8 for the worst candidate (*BgtE-5642*) to + 18 for the two best ones (*Bgt_avrF2_9* and *BgtE-20041*) (Supplementary Note 1, Supplementary Data 1, Supplementary Fig. 1). *AvrPm2* and *AvrPm3*^*a2/f2*^ were used as a control for assessing *Avr* candidate scoring, and they ranked in the top 20, with a total score of +17 and +16, respectively. We selected all effector genes scoring at least +10 (i.e. 100 candidates) for further functional validation (Supplementary Data 1).

### Identification of *AvrPm3* candidates using GWAS

**Fig. 1:**
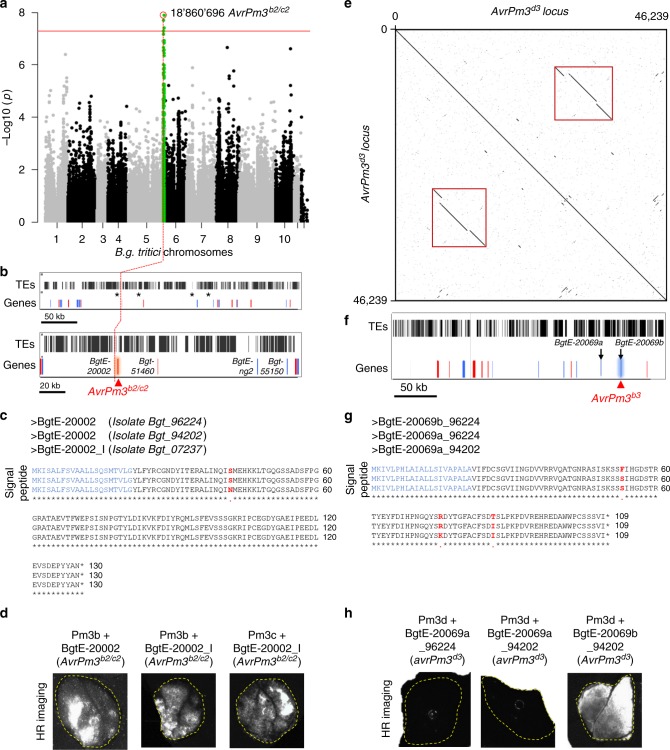
Identification and functional validation of *AvrPm3*^*b2/c2*^ and *AvrPm3*^*d3*^. **a**–**d** Identification and functional validation of *AvrPm3*^*b2/c2*^. **a** GWAS based on 26 avirulent and 74 virulent isolates on *Pm3b*. The peak for *AvrPm3*^*b/c*^ on chromosome 5 is indicated in green. **b** TE content of the *AvrPm3*^*b2/c2*^ locus is indicated (black bars), gene content in blue for polymorphic genes between Bgt_96224 and Bgt_94202 and red for non-polymorphic genes. The four effector genes selected for functional validation are indicated with asterisks (upper panel) and labelled in the lower panel. Position of the GWAS peak **is** indicated (dotted red line). The functionally validated AvrPm3b2/c2 gene is highlighted. **c** Alignment of the AVRPM3^B2/C2^ protein sequence encoded by BgtE-20002 in the isolates Bgt_96224, Bgt_94202 and Bgt_07237. The sequence corresponding to the signal peptide (blue), and polymorphic residues (red) are indicated. **d** Functional validation of the *AvrPm3*^*b2/c2*^-*Pm3b/c* interactions using transient expression assays in *N. benthamiana*. HR was scored 4–5 days after co-infiltration (Fusion-FX imager). **e**–**h** identification and functional validation of *AvrPm3*^*d3*^. **e** Dot-plot depiction of the duplicated region on chromosome 9 (red frame) from isolate Bgt_96224, containing *AvrPm3*^*d3*^ (*BgtE-20069b*) and its paralog *BgtE-20069a*. **f** Relative position of the *BgtE-20069a* and *BgtE-20069b* in the *AvrPm3d* locus. TE content is indicated (black bars), polymorphic genes between Bgt_96224 and Bgt_94202 are indicated in **blue** and non-polymorphic in red. Functionally validated *AvrPm3*^*d3*^ gene is highlighted. **g** Alignment of the AVRPM3^D3^ protein sequences encoded by BgtE-20069b, and the duplicated BgtE-20069a paralog from Bgt_96224, and Bgt_94202. **h** Functional validation of *AvrPm3*^*d3*^ in transient assays in *N. benthamiana*. HR was scored 4–5 days after co-infiltration (Fusion-FX imager). All effector constructs were codon optimised for expression in *N. benthamiana*, synthesised without the signal peptide, and recombined into the pIPKb004 expression vector. All *Pm3* alleles were expressed as native wheat sequences in the pIPKb004 expression vector. Complete *N. benthamiana* leaf pictures are provided in a Source Data File. Results are consistent over at least two independent assays each consisting of 6–8 independent leaf replicates

In a second approach to identify *AvrPm3*^*b*^, *AvrPm3*^*c*^ and *AvrPm3*^*d*^ candidates, we sequenced 72 additional wheat powdery mildew genomes to complete a GWAS (genome wide association study) mapping population of 100 races originating exclusively from China (Supplementary Data 2). Genetic association between sequence polymorphisms and differences in virulence/avirulence patterns on *Pm3b* and *Pm3d* was assessed using the Genome Association and Prediction Integrated Tool (GAPIT)^33^. We found no significant association for *AvrPm3*^*d*^. We found significantly associated SNPs for *AvrPm3*^*b*^ on chromosome 5^34^, with a peak at position 18,860,696 (Fig. 1a, b). This region overlaps with the physical position of *Locus_3*, a genetic locus that has been previously described as encoding for the *AvrPm3*^*b2*^ and *AvrPm3*^*c2*^ specificities^28^ (Supplementary Note 2). The position of the *AvrPm3*^*b2*^ peak was located within the genetic interval defined by the *Locus_3* flanking markers M049LE and ctg118_21 in the powdery mildew consensus genetic map^28^ (Supplementary Fig. 2).

We used a combination of resources to thoroughly annotate this genetically complex locus (Supplementary Note 2). We found in total four candidate effector genes in the locus: *Bgt-51460*, *BgtE-20002*, *Bgt-55150* and *BgtE-ng2*, all members of the effector gene family E018^34^ (Supplementary Fig. 3). *Bgt-51460* is not expressed and not polymorphic between the *Pm3b/c* avirulent Bgt_96224, and the *Pm3b/c* virulent Bgt_94202 parents. *BgtE-20002* is not polymorphic between the isolates Bgt_96224 and Bgt_94202, and it was also identified as a candidate *Avr* by the benchmarking approach with an overall score of +12 (Supplementary Data 1). An initial search for sequence variants in a few isolates from the Swiss population, led to the identification of *BgtE-20002_I*, a haplotype from isolate Bgt_07237 (avirulent on *Pm3b*, *Pm3c* and *Pm3d*) differing from the *BgtE-20002* variant by 1 non-synonymous SNP (Fig. 1c). *Bgt-55150* carries 2 SNPs leading to amino acid changes distinguishing the *Bgt-55150* alleles from the avirulent Bgt_96224 and the virulent Bgt_94202 isolates (Supplementary Fig. 4). *BgtE-ng2* is also polymorphic with one functional allele, *BgtE-ng2b*, encoded by the virulent isolate Bgt_94202, while the allele encoded by the avirulent Bgt_96224 isolate, *BgtE-ng2a* is disrupted by a transposable element insertion (Supplementary Fig. 4). Based on the GWAS results, genetic information from the powdery mildew consensus map^28^, and thorough annotation of *Locus_3*, we selected *Bgt-51460, BgtE-20002*, *BgtE-20002_I, Bgt-55150a, Bgt-55150b* and *BgtE-ng2b* for further functional validation (Supplementary Note 2, Supplementary Data 3 and 4).

### Functional validation of *AvrPm3*^*b2/c2*^ and *AvrPm3*^*d3*^

To functionally validate the *AvrPm3* candidates identified by effector benchmarking and GWAS, we took advantage of our well established agrobacterium-infiltration assay in *N. benthamiana*^28–30^, allowing transient, heterologous, co-overexpression of effector and NLR proteins. One hundred candidates from the effector benchmarking and all six candidates from the GWAS were codon optimised for expression in *N. benthamiana* (Supplementary Data 3), synthesised without the signal peptide, and screened for recognition by *Pm3b*, *Pm3c* and *Pm3d* using our transient assays in *N. benthamiana* leaves (see Methods). We took advantage of the high sensitivity reading (HSR) technology embedded in the Fusion FX fluorescence imager (www.vilber.com) to quantitatively score the hypersensitive response 5 days after infiltration^29,30^. One *Avr* candidate, *BgtE-20002*, commonly found by benchmarking and GWAS, induced strong HR when combined with *Pm3b* and weak HR with *Pm3c*, while the sequence variant *BgtE-20002_I*, induced strong HR both with *Pm3b* and *Pm3c* (Fig. 1d, Supplementary Fig. 5a, b). We observed no HR when both variants were co-infiltrated with *Pm3a*, *Pm3f*, *Pm3d* and *Pm3e*, indicating that this effector is implicated in dual and specific recognition by the *Pm3b* and *Pm3c* alleles that have previously been shown to exhibit overlapping recognition spectra, with *Pm3c* representing the weaker allele^35^. Therefore, we concluded that *BgtE-20002* is *AvrPm3*^*b2/c2*^, the *Avr* recognised by *Pm3b* and *Pm3c*. Also, because *BgtE-20002* is not polymorphic between the *Pm3b/c* avirulent isolate Bgt_96224 and the virulent isolate Bgt_94202, we conclude that *AvrPm3*^*b2/c2*^ is genetically suppressed by the active *SvrPm3*^*a1/f1*^ allele encoded in Bgt_94202, in a similar way as *AvrPm3*^*a2/f2*^^28,32^^,^.

A second *Avr* candidate, *BgtE-20069b*, only found in the benchmarking list, and encoded within a segmental duplication on chromosome 9 (Fig. 1e–g), induced HR in combination with *Pm3d* (Fig. 1h, Supplementary Fig. 5c). No HR was observed when *BgtE-20069b* was combined with *Pm3a*, *Pm3f*, *Pm3b*, *Pm3c* and *Pm3e*, indicating that this effector gene is *AvrPm3*^*d3*^, the *Avr* recognised by *Pm3d*. The *BgtE-20069b* copy corresponding to *AvrPm3*^*d3*^, is deleted in the virulent isolate Bgt_94202. Its paralog, *BgtE-20069a*, has two non-synonymous SNPs compared to the *Avr*, and it is polymorphic between Bgt_96224, and Bgt_94202 (Fig. 1g). We used the same transient assays in *N. benthamiana* to test for possible recognition of both alleles of *BgtE-20069a* by *Pm3d*, and we observed no HR (Fig. 1h). All together this data demonstrates that specificity of the *AvrPm3*^*d3*^*-Pm3d* interaction is based on specific recognition of one paralog of a duplicated effector gene, *BgtE-20069b*.

**Fig. 2:**
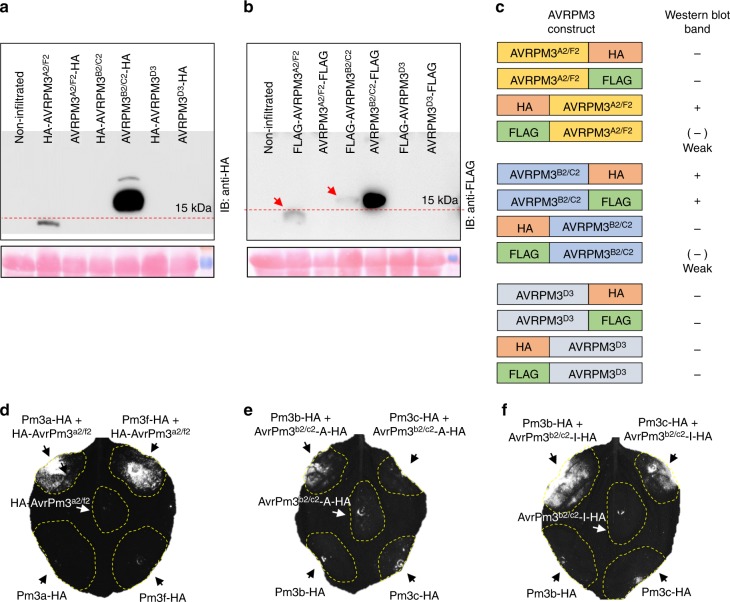
Impact of epitope fusion of the AVRPM3 protein expression and detectability. **a**, **b** Western blot detection of C and N terminal HA (**a**) and FLAG (**b**) epitope tag fusion of AVRPM3^A2/F2^, AVRPM3^B2/C2^ and AVRPM3^D3^ (upper panel) and Ponceau staining of the Western blot membrane (lower panel) are depicted. Uncropped western blot images are provided in a Source Data File. **c** Graphical summary of AVRPM3 protein tagability based on detection of the protein on a western blot, demonstrating significantly different impacts of epitope fusions based on tag position and sequence. **d**–**f** experimental assessment of HA tagged AVR variants in functional validation assays in *N. benthamiana*. Protein expression data is provided in Supplementary Fig. 6. HA-AVRPM3^A2/F2^ (**d**), AVRPM3^B2/C2^-A-HA (**e**) and AVRPM3^B2/C2^-I-HA (**f**), co-expressed with their respective NLRs. HR was assessed using HSR imaging 4–5 days after *Agrobacterium* infiltration. Results are consistent over at least two independent assays each consisting of 6–8 independent leaf replicates

In a last series of functional validation assays, we attempted epitope tagging of AVRPM3^A2/F2^, AVRPM3^B2/C2^ and AVRPM3^D3^. All effector proteins were C and N terminally fused to HA and FLAG epitope tags, and subsequently tested for (i) detectability on a western blot, and (ii) functionality in the *N. benthamiana* assay (Fig. 2, Supplementary Note 3). For AVRPM3^A2/F2^, N terminal HA and FLAG fusions were detectable but not the C terminally tagged constructs. We observed the opposite for AVRPM3^B2/C2^, where the C terminal HA and FLAG fusion were detectable but not the N terminal ones. For AVRPM3^D3^, none of the epitope fusions was detectable on the western blot (Fig. 2a–c). We have also tested the functionality of the detectable HA-AVRPM3^A2/F2^, AVRPM3^B2/C2^-HA constructs in co-infiltration assays with their respective PM3 NLRs, and found that these epitope fusions did not interfere with AVR function (Fig. 2d–f, Supplementary Fig. 6). Together, these assays demonstrate that AVRPM3 protein function is sensitive to epitope fusions in a tag sequence and position-dependent manner.

### *AvrPm3* recognition is suppressed by Svr and NLR interactions

**Fig. 3:**
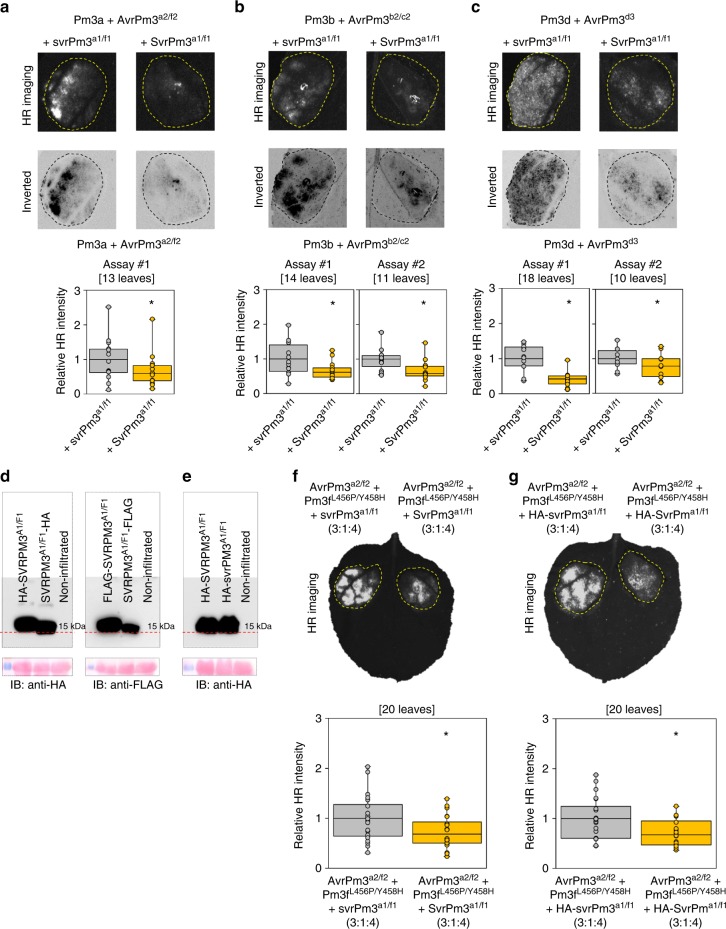
Suppression of *AvrPm3-Pm3* recognition by *SvrPm3*^*a1/f1*^. **a-c** Suppression of the recognition of *AvrPm3*^*a2/f2*^ (**a**), *AvrPm3*^*b2/c2*^ (**b**) and *AvrPm3*^*d3*^ (**c**) in presence of the active allele of the *SvrPm3*^*a1/f1*^ suppressor (right spot). Ratios of *Avr-R-Svr* mixes for *AvrPm3*^*a2/f2*^, *AvrPm3*^*b2/c2*^ and *AvrPm3*^*d3*^ were 3:1:4, 4:1:4, and 2:1:4 respectively. HR is compared to a negative control in presence of the inactive suppressor allele *svrPm3*^*a1/f1*^ (left spot). Results are consistent over at least two independent assays each consisting of at least 10 independent leaf replicates. HR quantification in presence of the inactive vs. active suppressor is depicted in the lower panels. Number of independent leaf replicates is indicated. Complete *N. benthamiana* leaf pictures are provided in Source Data File. **d** Western blot detection of C and N terminal HA and FLAG epitope fusions of the active SVRPM3^A1/F1^ and Ponceau staining of the membrane (lower panel) are depicted. **e** Western blot detection HA-SVRPM3^A1/F1^ and HA-svrPM3^A1/F1^ suppressors demonstrating there is no difference in protein abundance between active vs. inactive alleles, respectively. Uncropped Western blot images are provided in Source Data File. **f**,**g** Functionality of tagged HA-svrPm3^a1/f1^ and HA-SvrPm3^a1/f1^ (**g**) compared to non-tagged svrPm3^a1/f1^ and SvrPm3^a1/f1^ (**f**). HR is induced by combining AvrPm3^a2/f2^ and Pm3f ^L456P/Y458H^. Suppression is assessed by comparing active vs. inactive suppressors, as originally described in Bourras et al.^28^. *Avr-R-Svr* ratios are indicated. HR was measured using HSR imaging 3 days post *N. benthamiana* agro-infiltration (f-g, upper panel). HR Quantification from the same assays revealed by HSR imaging (f-g lower panels). In both cases, the presence of the active suppressor allele (SvrPm3^a1/f1^) resulted in significant reduction of HR as compared to the inactive alleles (svrPm3^a1/f1^) independently of the epitope, demonstrating that HA-svrPm3^a1/f1^ and HA-SvrPm3^a1/f1^ are functional. Number of independent leaf replicates is indicated. Statistical significance in a-c, f, g was assessed with a two-sided Student t-Test for paired data and indicated with (*; p < 0.05). Mean values are indicated by the middle line in the boxplot. Individual data points are plotted along the whiskers delineating minimum and maximum values. Raw data underlying the reported averages in a-c, f, g are provided in a Source Data File

Genetic studies in F1 segregating populations of wheat powdery mildew have revealed that *SvrPm3*^*a1/f1*^ is genetically suppressing several *Avrs* of the *Pm3* genes (including *AvrPm3*^*b2/c2*^ and *AvrPm3*^*d3*^), but so far functional evidence was only provided for *AvrPm3*^*a2/f2*^^28^^,^. Here, with the cloning of two novel *AvrPm3* genes, we had a unique opportunity to functionally validate that *SvrPm3*^*a1/f1*^ can act as a suppressor of *Avr* recognition for additional (probably all) *AvrPm3* genes^28^. We co-expressed *AvrPm3*^*b2/c2*^, and *AvrPm3*^*d3*^ with *Pm3b* and *Pm3d*, respectively, in presence of the active *SvrPm3*^*a1/f1*^ allele originating from the mildew isolate Bgt_94202^28^. HR was assessed relative to a control where the active *SvrPm3*^*a1/f1*^ suppressor allele was replaced by the inactive *svrPm3*^*a1/f1*^ allele encoded by the mildew isolate Bgt_96224, as previously described^28^. As an additional control, we also assayed suppression of the *AvrPm3*^*a2/f2*^*-Pm3a* interaction using the same experimental set-up (see Methods). As previously demonstrated^28^, *AvrPm3*^*a2/f2*^ recognition was suppressed by *SvrPm3*^*a1/f1*^ and resulted in significant reduction of HR intensity in our control assay (Fig. 3a), thus demonstrating the functionality of our improved experimental system based on codon-optimised constructs and HSR imaging. Consistent with previous genetic evidence from Bourras and colleagues^28^, we found in two independent assays that recognition of *AvrPm3*^*b2/c2*^ and *AvrPm3*^*d3*^ was also suppressed by *SvrPm3*^*a1/f1*^ and resulted in significant reduction of HR intensity (Student *t*-test, *p*-value < 0.05) (Fig. 3b, c). We also produced C and N terminal HA and FLAG epitope tag fusions of the active SVRPM3^A1/F1^ variant and found that all constructs were detectable on the western blot, suggesting significant differences between AVRPM3 and SVRPM3 proteins in terms of tolerance to epitope fusions (Fig. 3d). We have therefore tagged the inactive svrPM3^A1/F1^ variant that we use as negative control for AVRPM3 suppression, and tested for protein expression levels as compared to the active suppressor. We found no difference in protein abundance between HA-SVRPM3^A1/F1^, and HA-svrPM3^A1/F1^ (Fig. 3e), and we could show that the tag is also not interfering with SVRPM3^A1/F1^ function (Fig. 3f, g). These data demonstrate that differences between active and inactive suppressor variants are not due to differences in protein expression. Based on this data, we could confidently test the hypothesis that *SvrPm3*^*a1/f1*^is possibly acting as a transcriptional or translational suppressor of *AvrPm3* or *Pm3*, based on a previous observation suggesting this effector encodes a fungal ribonuclease-like protein^32^. To do so, we tested protein expression levels of AVRPM3^A2/F2^, AVRPM3^B2/C2^ and individual PM3 NLRs in presence vs. absence of SVRPM3^A1/F1^ and found no differences (Supplementary Fig. 7). These results demonstrate that *SvrPm3*^*a1/f1*^ can indeed act as a suppressor of recognition of several *AvrPm3* genes, and suggest this function is not based on an *AvrPm3* or *Pm3*-specific ribonuclease activity but occurs at the level of protein–protein interactions.

In addition to gene suppression based on the action of *SvrPm3*^*a1/f1*^, evidence from genetic studies in wheat and transient assays with auto-activated PM3 constructs^26,28^, indicated that the *AvrPm3-Pm3* interactions can also be suppressed by inter-allelic interactions between the PM3 NLRs. Here, with the cloning of additional *AvrPm3* genes and the availability of an improved cell death assay in *N. benthamiana*, we had a unique opportunity to test for inter-allelic NLR-NLR interactions in presence of the AVR proteins. We assayed the suppression activity of *Pm3a-f* and the non-functional ancestral allele *Pm3CS* in transient co-expression assays when each allele was combined with *AvrPm3*^*a2/f2*^*-Pm3a*, *AvrPm3*^*a2/f2*^*-Pm3f*, *AvrPm3*^*b2/c*2^-I*-Pm3b*, *AvrPm3*^*b2/c2*^-I-*Pm3c*, and *AvrPm3*^*d3*^*-Pm3d* (Supplementary Figs. 8 and 9, Supplementary Note 4). We found that *Pm3a* and *Pm3f* had no NLR suppression activity and were mostly suppressed by the other *Pm3* alleles. *Pm3e* was only active on the weaker *Pm3c* and *Pm3f* alleles, while *Pm3b*, *Pm3c* and *Pm3d* had reciprocal suppression capacity and acted as the strongest suppressors among the functional *Pm3* NLRs. Here we conclude that inter-allelic suppression occurs upon AVR-dependent activation of the PM3 NLRs, and it is NLR sequence dependent.

### The AVRPM3 proteins belong to a group of related effectors

*AvrPm3*^*b2/c2*^ and *AvrPm3*^*d3*^ are not members of the *AvrPm3*^*a2/f2*^ effector gene family E008 but belong to the mildew effector families E018 and E034, respectively^34^ (Supplementary Figs. 3 and 10). Sequence alignment of the AVRPM3^A2/F2^, AVRPM3^B2/C2^, and AVRPM3^D3^ full proteins showed that the three AVRs share low identity (14.5 to 19.9%, Supplementary Fig. 11). Sequence alignment of the three effector families revealed conservation of the signal peptide, a conserved Hydrophobic x C (HxC) motif, and a characteristic conserved pattern of alternating hydrophobic residues, (Supplementary Fig. 12). Analysis of the phylogenetic relationships between the AVRPM3 families suggests that AVRPM3^A2/F2^ and AVRPM3^B2/C2^ belong to the same superfamily which groups separately from AVRPM3^D3^, and all three families are phylogenetically distinct from the SVRPM3^A1/F1^ family (Supplementary Fig. 13).

The PM3 NLRs are highly similar on the protein level with only few amino acid differences^24^. We therefore hypothesised that a basis for specificity could be that the sequence unrelated AVRPM3 effectors are encoding for structurally similar proteins, based on conservation of amino-acid properties and the position of structural residues such as cysteines and prolines (Supplementary Fig. 12). We applied AVRPM3^A2/F2^, AVRPM3^B2/C2^, and AVRPM3^D3^ to secondary structure modelling using four different prediction algorithms available at the Quick2D platform (https://toolkit.tuebingen.mpg.de/#/tools/quick2d^36^,). All modelling methods have consistently predicted the presence of one alpha helix followed by three to four beta-sheets for all three AVRs (Supplementary Fig. 14a). To further substantiate these results, we applied the AVRPM3^A2/F2^, AVRPM3^B2/C2^, and AVRPM3^D3^ effector family members to three dimensional structural modelling using the RaptorX platform (raptorx.uchicago.edu^37^,). We found no statistically robust structural model common to all three families or to all members of the same family (Supplementary Data 5). However, despite differences in the templates assigned by RaptorX to individual members, we found that several of the tertiary folds predicted for the AVRPM3^A2/F2^ and AVRPM3^B2/C2^ families consisted of one central helix facing three to four beta-sheets, reminiscent of the previously predicted secondary folds (Supplementary Fig. 14b, c). In particular, this putative tertiary structure was also predicted for the AVRPM3^A2/F2^ and AVRPM3^B2/C2^ avirulence proteins. Therefore, based on protein alignments, phylogeny, and in silico secondary and tertiary structure modelling, we suggest that AVRPM3^A2/F2^ and AVRPM3^B2/C2^ are possibly encoding for structurally similar proteins. Finally, all *AvrPm3* genes are highly induced upon infection, and all encode for typical pathogen effector proteins (Supplementary Note 5, Supplementary Fig. 15). We therefore propose that the *AvrPm3* genes are possibly acting as *bona-fide* virulence factors suppressing host immunity.

### *AvrPm3*^*b2/c2*^ and *AvrPm3*^*d3*^ conservation in mildew populations

**Fig. 4:**
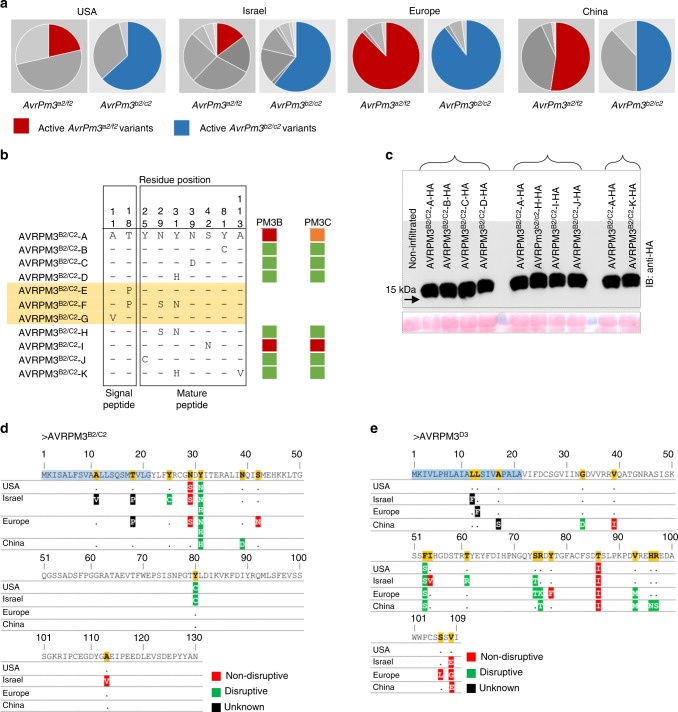
Functional characterisation of AVRPM3^B2/C2^ and AVRPM3^D3^ based on natural and artificial sequence variation. **a** Geographical distribution and frequency of *AvrPm3*^*b2/c2*^ sequence haplotypes encoding active AVRPM3^B2/C2^ virulence protein variant as compared to *AvrPm3*^*a2/f2*^. Underlying source data is provided in a Source Data File. **b** Summary of AVRPM3^B2/C2^ haplovariants, only polymorphic residues are depicted. Strong recognition by PM3B and PM3C based on *N. benthamiana* infiltration assays are indicated with a red box, and weak recognition with an orange box. All assays are based on expression of the mature protein after removal of the signal peptide. Therefore, haplovariants E and G, and haplovariant F were not tested because the mature protein sequence is identical to variant A and variant H, respectively. **c** Western blot detection (upper panel) of C terminal HA epitope fusion to the mature proteins of the AVRPM3^B2/C2^ haplovariants. Ponceau staining of the western blot membranes is depicted in the lower panel. Braces indicate samples where all constructs were combined on the same leaf and rotated together with AVRPM3^B2/C2^-A as a reference. Uncropped Western blot images are provided in a Source Data File. **d**, **e** Functional characterisation of the impact of natural sequence diversity of AVRPM3^B2/C2^ (**d**) and AVRPM3^D3^ (**e**) on AVR function

In a previous study, McNally and colleagues^29^ found limited sequence diversity for *AvrPm3*^*a2/f2*^ in worldwide mildew populations, and a high frequency of the recognised haplotype variant. These observations are contrasting with the general hypothesis arguing that avirulence genes are highly polymorphic in pathogen populations, and that active variants tend to be lost because of selective pressure to escape recognition from the host immune system^29^. To study the genetic diversity of *AvrPm3*^*b2/c2*^ and *AvrPm3*^*d3*^, we applied a collection of 185 powdery mildew isolates with a worldwide geographical distribution to haplotype mining as previously described by McNally and colleagues^29^. The full sequences of *AvrPm3*^*b2/c2*^ and *AvrPm3*^*d3*^ were recovered using genome sequencing data or PCR gene amplification and subsequent sequencing (see Methods). For *AvrPm3*^*b2/c2*^, we identified 10 non-synonymous mutations across the gene, including 8 in the mature protein resulting in 7 protein variants (Fig. 4a, b, Supplementary Fig. 16). Based on those haplotype sequences, we found a pN/pS (ratio of polymorphic nonsynonymous (pN) to polymorphic synonymous (pS) sites within a species) of 1.26 indicating that the *AvrPm3*^*b2/c2*^ gene is under diversifying selection, which is consistent with previous finding by Müller et al.^34^ and McNally et al.^29^ showing that the whole *AvrPm3*^*b2/c2*^ and *AvrPm3*^*a2/f2*^ families are under diversifying selection. All haplotypes of *AvrPm3*^*b2/c2*^ were codon optimised for expression in *N. benthamiana*, synthesised without the signal peptide and screened for recognition by *Pm3b* and *Pm3c*. No haplotype besides the previously validated *AvrPm3*^*b2/c2*^-A variant from Bgt_96224 and the *AvrPm3*^*b2/c2*^-I from Bgt_07237, induced HR when combined with *Pm3b* and *Pm3c* in *N. benthamiana* (Fig. 4b). Protein expression assays demonstrated that loss of AVRPM3^B2/C2^ recognition is not due to absence of the protein as all haplovariants are equally detectable on a western blot (Fig. 4c, Supplementary Fig. 17). We found that the *AvrPm3*^*b2/c2*^-I is only present in the Swiss population and represented by only one isolate. Interestingly, the *AvrPm3*^*b2/c2*^-A variant was the most frequent haplotype found in all subpopulations, and in two regions, USA and Israel, this active *Avr* variant was more frequent than the active *AvrPm3*^*a2/f2*^-A (Fig. 4a). For *AvrPm3*^*d3*^, we identified 17 non-synonymous mutations across the whole gene, including 15 in the mature protein. However, it was not possible to distinguish between the SNPs that should be assigned to a haplotype of the *BgtE-20069a* or the *BgtE-20069b* paralogs (i.e. *AvrPm3*^*d3*^) which only differ by two residues (Fig. 1g). Therefore, while we could not resolve the haplotype diversity of *AvrPm3*^*d3*^ which shows high level of copy number variation in mildew isolates (*AvrPm3*^*b2/c2*^ is present in a single copy only, see Supplementary Fig. 18), these results show that at least one paralog of the *AvrPm3*^*d3*^ effector can be found globally in all isolates. Here we conclude that data from diversifying selection studies and global population genetics provide further evidence substantiating that the *AvrPm3* effectors are important virulence factors.

We also assessed the individual impact on AVR recognition of the 7 amino-acid variations in the mature protein of AVRPM3^B2/C2^ found in our natural diversity (residue changes in the signal peptide were not tested) (Fig. 4d). For AVRPM3^D3^, 14 amino-acid variations were similarly assessed which very likely include polymorphic residues from its paralog BgtE-20069a, because these could not be distinguished in our haplotype mining (Fig. 4e). Every single amino acid variation was used to generate point mutants in AVRPM3^B2/C2^ and AVRPM3^D3^, and all were screened for recognition by their respective NLRs using transient assays in *N. benthamiana* as previously described^29^. We found that most of the single amino acid mutations disrupted the recognition of AVRPM3^B2/C2^ and AVRPM3^D3^ by their respective NLRs (Fig. 4d, e), suggesting that selective pressure to escape *R* gene recognition is a driving force for sequence diversification of AVRPM3^B2/C2^ and AVRPM3^D3^. Protein expression analysis demonstrated that loss of AVRPM3^B2/C2^ recognition is not due to absence of the protein as all point mutants are equally detectable on a western blot (Supplementary Fig. 19). Interestingly, we found that most of the polymorphic positions in AVRPM3^B2/C2^, including 3 out of the 4 disruptive mutations, are located towards the N-terminal end within a stretch of 18 residues (aa 25–42), immediately after the predicted signal peptide cleavage site (Fig. 4d). By contrast, most of the natural diversity in AVRPM3^D3^ and its duplication BgtE-20069a, including 5 out of the 8 residues disrupting recognition, is found within a stretch of 24 residues (aa 74–97) towards the C-terminal end of the protein (Fig. 4e). This data suggests that specific domains of the AVR proteins are involved in recognition, and these seem to be located in distinctly different regions of AVRPM3^B2/C2^ and AVRPM3^D3^.

**Fig. 5:**
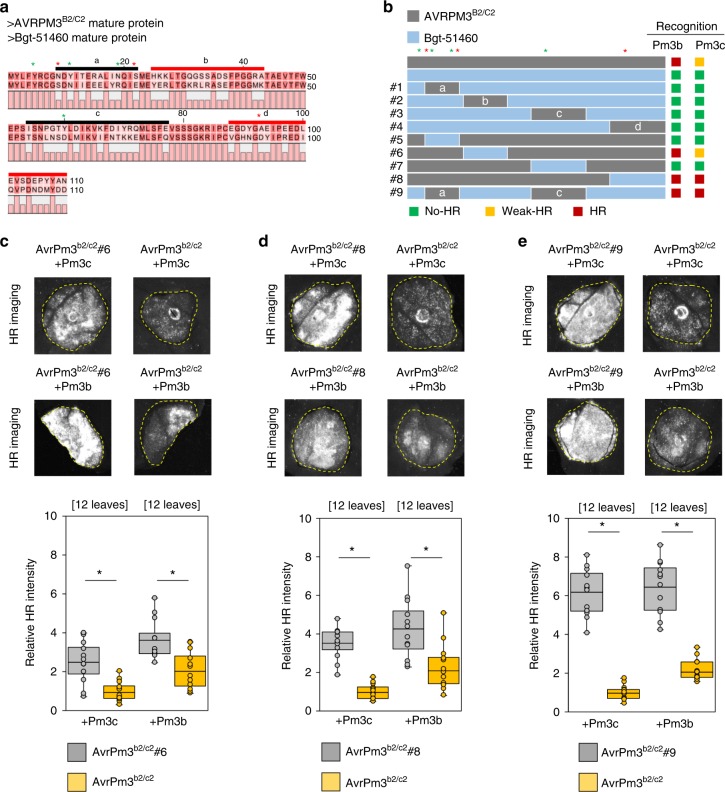
Consequence of synthetic domain swaps on the recognition of AVRPM3^B2/C2^. **a**–**b** Consequence of synthetic domain swaps on the AVRPM3^B2/C2^ AVR function. **a** Protein sequence alignment of the mature protein of AVRPM3^B2/C2^-A and the closest family member Bgt-51460. Swapped domains are indicated and labelled a, b, c and d. Domains that do not abolish AVR recognition when introduced from Bgt-51460 into AVRPM3^B2/C2^ are indicated in red. **b** Schematic representation of the protein domains swapped between AVRPM3^B2/C2^ (grey) and Bgt-51460 (blue). **a**, **b** Position of the residues identified from the natural sequence diversity (Fig. 4b, d) are indicated with asterisks. The impact of individual residues on AVR recognition is indicated with green for mutations with a disruptive effect, and red for mutations, with a neutral effect, according to the results summarised in Fig. 4b, d. **c**–**e** Transient expression assays in *N. benthamiana* indicating recognition (upper panel) and HR quantification (lower panel) for AVRPM3^B2/C2^ swaps #6 (**c**), swap #8 (**d**) and swap #9 (**e**) by *Pm3c*, and *Pm3b*. The number or independent leaf replicates is indicated. Mean values are indicated by the middle line in the boxplot. Individual data points are plotted along the whiskers delineating minimum and maximum values. Statistical significance was assessed with a two-sided Student’s *t*-test for paired data and indicated with (*p < 0.05). Complete *N. benthamiana* leaf pictures and raw data underlying the reported averages are provided in a Source Data File

We therefore designed domain swaps covering different stretches of residues polymorphic between AVRPM3^B2/C2^ and AVRPM3^D3^ and the closest members of their effector families, BGT-51460 and BGTE-5883, respectively (Fig. 5a, b, Supplementary Fig. 20a, b, Supplementary Note 6). For both AVR proteins we found that NLR-recognition is dependent on multiple distinct protein regions that overlap with regions with higher level of polymorphism in worldwide mildew accessions (Fig. 5b, Supplementary Fig. 20b). For AVRPM3^D3^, all single region exchanges resulted in loss of AVR recognition, except for one highly conservative swap (construct #6) where only a few residues at the C and N termini were exchanged with the corresponding sequence from BGTE-5883 (Supplementary Fig. 20b, c). For AVRPM3^B2/C2^, protein expression assay indicated all chimeric proteins can be detected on a western blot thus demonstrating that loss of recognition in our assays is not based on loss of AVR protein (Supplementary Fig. 21). We found that segments b and d from AVRPM3^B2/C2^, corresponding to regions of low levels of polymorphism, could be exchanged with the corresponding sequence from BGT-51460 without loss of AVR function (swap #6 and #8, Fig. 5b–d). Moreover, both chimeric proteins resulted in significantly stronger recognition by PM3B and PM3C as compared to the natural sequence (Fig. 5c, d). In one additional swap (construct #9), we simultaneously introduced segment a corresponding to the region with higher level of polymorphism, and segment c from AVRPM3^B2/C2^ to BGT-51460. Consistent with the observed natural diversity, chimeric protein #9 resulted in gain of AVR function by BGT-51460, and induced a much stronger recognition reaction by PM3B and PM3C as compared to the natural sequence (Fig. 5e). This further supports the hypothesis that overall protein structure, conserved between members of effector families, as well as specific contact regions are important for PM3-dependent recognition of AVR proteins, and that natural diversity studies can be used to predict such regions.

### The *Pm3* alleles can recognise AVRs from non-adapted mildews

**Fig. 6:**
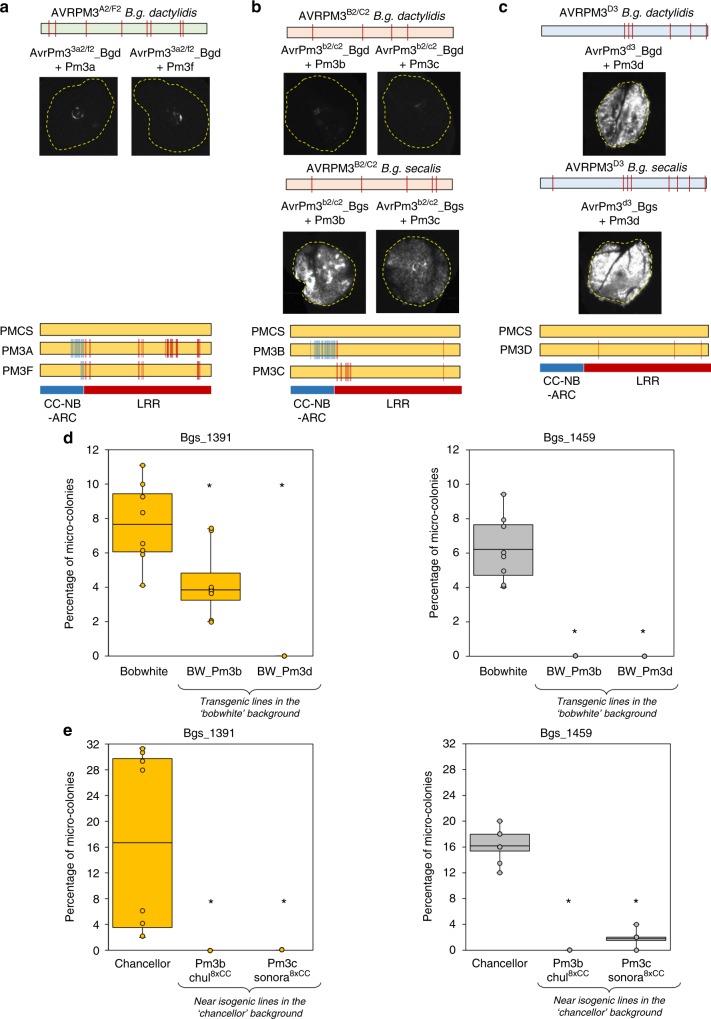
Conservation of *AvrPm3-Pm3* recognition in grass mildews **a**–**c** Transient expression assays combining the identified grass mildew homologues of AVRPM3^A2/F2^ (**a**), AVRPM3^B2/C2^ (**b**), and AVRPM3^D3^ (**c**), with their corresponding PM3 NLR receptors. Results are consistent over at least two independent assays each consisting of 6–8 independent leaf replicates. Complete *N. benthamiana* leaf pictures are provided in a Source Data File. Polymorphic residues compared to the *B.g. tritici* encoded sequences are schematically indicated with vertical bars. Similarly, polymorphic residues in the PM3 protein variants compared to the susceptible PM3CS sequence are indicated with vertical blue (CC-NB-ARC) and red (LRR) lines in the lower panel. **d**, **e** Percentage of micro-colonies formed by the non-adapted *B.g. secalis* isolates Bgs_1391 and Bgs_1459 on **d** transgenic lines expressing the *Pm3b* (BW_PM3b) and *Pm3d* (BW_Pm3d) resistance genes in the hexaploid wheat genotype ‘*Bobwhite*’, compared to the non-transgenic ‘*Bobwhite*’ control, **e** Near isogenic lines (NILs) expressing the *Pm3b* (Chul^8xCC^) and *Pm3c* (Sonora^8xCC^) resistance loci backcrossed 8 times with the hexaploid wheat genotype ‘*Chancellor*’, compared to a ‘*Chancellor*’ control. Both ‘*Bobwhite* and ‘*Chancellor*’ are fully susceptible to adapted *B.g. tritici* races. Micro-colony formation was scored microscopically 2 days post rye mildew infection (see Methods). Values are representative of the average from eight independent leaf replicates. Mean values are indicated by the middle line in the boxplot. Individual data points are plotted along the whiskers delineating minimum and maximum values. Differences to the control were statistically tested with the Wilcoxon Rank Sum Test. Statistical significance (*p* < 0.05) is indicated with a star. Raw data underlying the reported averages are provided in a Source Data File

There has been accumulating genetic evidence suggesting that *Avr-R* interactions are playing an important role as determinants of host-specificity in cereal mildews (Supplementary Note 7). Therefore, we aimed at investigating the role of the *Pm3* alleles as possible determinants of host specificity to *B.g. secalis* and *B.g. dactylidis*, two mildew *formae speciales* only growing on rye (*Secale cereale*) or the wild grass *Dactylis glomerata*, respectively. We took advantage of the availability of the genome sequences of 5 isolates of rye mildew and 2 of *Dactylis* mildew to recover the sequences of the homologues of *AvrPm3*^*a2/f2*^, *AvrPm3b2*^*b2/c2*^ and *AvrPm3*^*d3*^. We found homologues of all three *AvrPm3* genes in *B.g. dactylidis* (hereafter referred to as *AvrPm3*^*a2/f2*^*-Bgd, AvrPm3*^*b2/c2*^*-Bgd,* and *AvrPm3*^*d3*^*-Bgd*) and homologues of *AvrPm3b*^*b2/c2*^ and *AvrPm3*^*d3*^ in *B.g. secalis* (*AvrPm3b2*^*b2/c2*^*-Bgs,* and *AvrPm3*^*d3*^*-Bgs*) (Supplementary Fig. 22a–c). We found no homologue of *AvrPm3*^*a2/f2*^ in rye mildew. RNA-Seq data from two rye mildew isolates S-1391 and S-1459^38^ shows that *AvrPm3b2*^*b2/c2*^*-Bgs* and *AvrPm3*^*d3*^*-Bgs* are expressed during compatible interaction between *B.g. secalis* and rye (Supplementary Fig. 22d, e). All *AvrPm3* homologues were tested for *R* gene recognition using the transient assay in *N. benthamiana* (Fig. 6a–c, Supplementary Fig. 23a–g). We observed no HR when *AvrPm3*^*a2/f2*^*-Bgd* was co-expressed with *Pm3a* or *Pm3f* (Fig. 6a). For *AvrPm3*^*b2/c2*^, co-expression of the homologue from *B.g. dactylidis* with *Pm3b* and *Pm3c* resulted in no HR, while interestingly the homologue from *B.g. secalis* was recognised by both NLRs (Fig. 6b). Similarly, both *AvrPm3*^*d3*^ homologues from rye and *Dactylis* powdery mildews were recognised by *Pm3d* (Fig. 6c). For AVRPM3^B2/C2^-BGD, protein expression data indicate that loss of recognition is not due to absence of the proteins (Supplementary Fig. 23j, k). However, data for AVRPM3^A2/F2^-BGD indicate this protein is low expressed in our assay (Supplementary Fig. 23h,i), therefore we cannot exclude that *AvrPm3*^*a2/f*2^-*Bgd* *-Bgd* is also recognised by *Pm3a/f* if expressed at higher levels.

The recognised AVRPM3^B2/C2^-BGS, AVRPM3^D3^-BGS and AVRPM3^D3^-BGD homologues differ by 4, 7 and 6 residues in the mature protein from their *B.g. tritici* encoded homologues respectively, demonstrating a higher level of sequence divergence as compared to the haplotypes of the *AvrPm3* genes in *B.g. tritici*. Finally, we also tested possible recognition of the rye and *Dactylis* mildew *AvrPm3* homologues by the rye powdery mildew resistance gene *Pm8* in transient assays in *N. benthamiana* and observed no HR (Supplementary Fig. 24). Altogether, this data indicates strong conservation of *AvrPm3* function and recognition specificity in grass mildews, and suggests that highly specific *AvrPm3-Pm3* interactions are involved in defence against non-host mildew forms.

To further substantiate these results, we designed a micro-phenomics assay to test for wheat susceptibility to non-host mildews in presence vs. absence of the *Pm3* genes. Transgenic wheat lines expressing the *Pm3b* and *Pm3d* resistance alleles were infected with two isolates from the non-adapted rye powdery mildew (Bgs_1391 and Bgs_1459). As no macroscopic growth or sporulation of rye mildew can be observed in this non-host interaction, we microscopically phenotyped these assays for micro-colony formation, i.e., the ability of mildew to establish a haustorium and few secondary hyphae (see Methods). Transgenic lines were compared to the non-transgenic ‘Bobwhite’ genotype, i.e., the identical genetic background of the *Pm3b* and *Pm3d* transgenics. We found a significant reduction of the ability of the non-adapted rye powdery mildew to form micro-colonies on the transgenic lines as compared to the non-transgenic control (Fig. 6d). To further extend these observations, we performed the same assay using the *Pm3b* and *Pm3c* near isogenic wheat lines (NILs) ‘Chul_8xChancellor’ and ‘Sonora_8xChancellor’, respectively. The backcrossing line ‘Chancellor’ corresponding to the genetic background of the *Pm3b* and *Pm3c* NILs, was used as a control. In agreement with our first assay, we also found a significant reduction of micro-colony formation on the *Pm3* NILs as compared to the control (Fig. 6e), further substantiating that the *Pm3b,c,d* alleles are acting as determinants of host specificity for non-adapted mildews. Finally, we also cloned and assayed the suppressor activity of the *SvrPm3*^*a1/f1*^ homologue encoded in the Bgs_1391 and Bgs_1459 genomes. We found these isolates encode for the previously described *SvrPm3*^*a1/f1*^*-J* haplotype, which differs by only one residue from the *B.g. dacylidis* sequence (Supplementary Fig. 25a). RNA-Seq data indicated the rye mildew encoded *SvrPm3*^*a1/f1*^*-J* is expressed at very low levels upon infection of rye (Supplementary Fig. 25b). Using transient assays in *N. benthamiana*, we could show that this variant is indeed an inactive suppressor comparable to the previously described *SvrPm3*^*a1/f1*^-C from the *Pm3* avirulent wheat mildew isolate Bgt_96224 (Supplementary Fig. 25c).

We conclude that, (i) specific recognition of divergent *AvrPm3* homologues from rye and *Dactylis* mildews by the *Pm3b,c,d* alleles, (ii) increased non-host resistance to rye-mildew isolates encoding active *AvrPm3* homologues in presence of the *Pm3* alleles, and (iii) presence of an inactive *SvrPm3*^*a1/f1*^ suppressor variant in those same isolates, is strong evidence supporting the role of the *Pm3* alleles as determinants of host-specificity in cereal mildews based on specific and conserved *AvrPm3-Pm3* interactions.

## Discussion

Allelic series of resistance genes are highly informative to molecularly characterise resistance function if they are studied both on the host and the pathogen side. Genetic work in the 1990s and more recent molecular analysis has suggested that allelic series of resistance genes with distinct specificities are very promising systems to dissect the molecular basis of complex, race-specific interactions of biotrophic fungal pathogens such as rusts or mildews with their hosts^10^. The analysis of recognition specificity in the cereal powdery mildew and the flax rust systems based on allelic series of resistance genes has revealed additional layers of complexity to the original gene-for-gene interaction model postulated by Flor^11^, because of the presence of suppressor/inhibitor genes and other modifiers of the interaction^10^. In addition to the *Pm3* allelic series of 17 race-specific resistance alleles to wheat mildew^25^, there are three other well studied examples of true allelic series of functionally different resistance specificities: the downy mildew (*Hyaloperonospora arabidopsidis*) gene *RPP13* in *Arabidopsis thaliana*^39^, the flax rust (*Melampsora lini*) resistance gene *L*^22^, and the *Mla* genes in barley (*Hordeum vulgare*)^27^. The *Pm3* alleles stand-out with a particularly high level of sequence conservation ( > 97% identity at the protein level)^25,40^, while the *Mla* alleles have 84.6% sequence identity and are as divergent as the *Arabidopsis* RPP13 and the flax *L* allelic variants^22,27,39^.

Whereas several allelic variants of RPP13 and the flax rust NLRs L5, L6 and L7 detect allelic AVR proteins, there is evidence that additional members of these allelic series also recognise sequence unrelated effectors^23,41,42^. This principle is further exemplified by MLA1 and MLA13, which show >91% identity on the protein level but recognise the sequence unrelated *Bgh* proteins AVR_A1_ and AVR_A13_^31^. Consistent with these findings, the so far identified AVR proteins of the *Pm3* allelic series AVRPM3^A2/F2^, AVRPM3^B2/C2^ and AVRPM3^D3^ are members of three distinct *B.g. tritici* effector gene families with low sequence identity, and belong to large groups of phylogenetically related effectors with a possible common origin (in particular AVRPM3^A2/F2^ and AVRPM3^B2/C2^) (Supplementary Figs. 11 and 13). Together, phylogenetic relatedness, positional conservation of amino-acid with similar properties and structural residues across AVRPM3 families (Supplementary Fig. 12), and possible similarities of tertiary protein folds (Supplementary Fig. 14), suggests that specificity of the *Pm3* alleles is possibly based on recognition of structurally similar effectors. This is reminiscent of the identification of diverse, but structurally conserved MAX effectors in different phytopathogenic fungi^43^, and would suggest it is plausible that AVRPM3^D3^, which stands out phylogenetically, would also share the same structure as AVRPM3^A2/F2^ and AVRPM3^B2/C2^. Here, the detection of structurally similar proteins might provide the plant with a crucial benefit in an evolutionary arms race with rapidly evolving fungal proteins, which are restricted only by the requirement to conserve important virulence functions that might be mainly dependent on protein structure. Thus, the AVRPM3-PM3 interactome might provide a highly suitable model for the study of rapid protein sequence evolution in eukaryotes under possibly the only constraint of structural conservation.

Recent studies of effector functions in barley powdery mildew have established the role of effectors as important virulence factors (reviewed in ref. ^14^) that have the ability to interfere with components of the host basal metabolism and host immunity^16–19^. High frequency of active *AvrPm3* haplotypes among wheat mildew populations, and the fact that homologues can also be found in the related *formae speciales B.g. secalis* and *B.g. dactylidis*, suggest that all *AvrPm3* genes are important for mildew virulence. Despite strong indication of diversifying selection on *AvrPm3*^*b2/c2*^ and as previously shown on *AvrPm3*^*a2/f2*^^29^^,^ no presence–absence polymorphisms or transposable element insertions could be detected for *AvrPm3*^*a2/f2*^^29^^,^ or *AvrPm3*^*b2/c2*^ (this study) in 272 and 185 *B.g. tritici* isolates respectively. Even in the case of *AvrPm3*^*d3*^ with the avirulent version being absent in numerous isolates, no cases of complete loss of the effector could be identified, thus indicating that the *AvrPm3* effectors are always present together in all wheat powdery mildew isolates globally. This stand in strong contrast to *AvrPm2*, *Avr*_*a1*_, *Avr*_*a13*_ and *AvrPib* from *B.g. tritici*, *B.g. hordei* and *Magnaporthe oryzae*, respectively, where loss of avirulence was exclusively (*AvrPm2*), predominantly (*AvrPib*) or partially (*Avr*_*a1*_ and *Avr*_*a13*_) found to be dependent on presence/absence polymorphisms, gene disruptions by transposable element insertions, and segmental deletions^30,31,44^. These findings suggest there is a selective advantage for the pathogen to simultaneously encode all three effectors. We suggest that the inability to completely lose these effector genes is compensated partially by the identified diversifying selection, giving rise to new, unrecognised variants, and in parts by the presence and activity of *SvrPm3*^*a1/f1*^ that allows the wheat powdery mildew fungus to mask, and preserve effectors that would otherwise be recognised by the *Pm3* alleles. Previous studies in a few mildew isolates indicated that suppression of *AvrPm3* recognition is only effective when the active *SvrPm3*^*a1/f1*^gene is highly expressed^28,29^. In this study, we did not determine the presence and expression levels of *SvrPm3*^*a1/f1*^ in the GWAS population. We propose that future integration of transcriptome data in genetic association and population genomics studies will provide important information on the role of *SvrPm3*^*a1/f1*^ in controlling both race-specificity and host-specificity in global cereal mildew populations. For AVRPM3^B2/C2^, domain swap studies identified two regions with higher levels of polymorphism in the mildew population that are sufficient for conferring AVR function, and two additional segments quantitatively affecting the strength of AVR recognition. McNally and colleagues^29^, also found that specific domains of AVRPM3^A2/F2^ are involved in immune receptor recognition, including one region quantitatively affecting the strength of AVR recognition. We suggest these domains are possibly acting as contact regions affecting AVR-R recognition and binding affinity, which is reminiscent of the identification of 4 residues, spanning a 21 amino-acid region in the rice blast avirulence protein AVR-Pik and affecting recognition and binding affinity to rice Pik resistance protein^45^.

The evolutionary history of grass powdery mildews has been reconstructed recently. Menardo and colleagues^13^ found that the radiation of the *B.g tritici*-clade, consisting of *B.g. tritici*, *B.g. secalis* and *B.g. dactylidis* has occurred 170,000 to 280,000 years ago and involved a host jump from wheat to *Dactylis glomerata*, a wild grass species, giving rise to *B.g. dactylidis*. The recent radiation of the *tritici*-clade is also reflected in their respective *Avr*-effector complement. Of the four *Avr* genes that have been identified in *B.g. tritici*, homologues can also be found in either *B.g. secalis* (*AvrPm2*), *B.g. dactylidis* (*AvrPm3*^*a2/f2*^) or both closely related *formae speciales* (*AvrPm3*^*b2/c2*^, *AvrPm3*^*d3*^). Furthermore, all *Avr* homologues from *B.g. secalis* and the *AvrPm3*^*d3*^ homologue from *B.g. dactylidis* are recognised by the corresponding *Pm3* alleles from wheat^30^ (this study). The recognised variants of *AvrPm3*^*b2/c2*^ from *B.g. secalis* and *AvrPm3*^*d3*^ from *B.g. secalis* and *B.g. dactylidis* contain 4, 7 and 6 non-synonymous SNPs in the mature peptide, respectively, when compared to the *Pm3*-recognised *B.g. tritici* variants. This stands in strong contrast to the rather limited *AvrPm3* variation found within wheat mildew populations, where single non-synonymous mutations often result in evasion of recognition. We hypothesise that this reflects the fact that the currently active *Pm3* alleles have evolved only after wheat domestication ~11,000 years ago^24^, and the *AvrPm3* homologues in closely related *formae speciales* have therefore not been targets of diversifying selection due to presence of the *Pm3* alleles in corresponding rye or *Dactylis* host species.

The presence of the *Pm3* alleles does severely restrict, often completely abolish the formation of non-sporulating micro-colonies by the non-adapted rye mildew (Fig. 6d, e). This is consistent with a previous study using a genetic cross between wheat and rye powdery mildews that has predicted over 20 years ago the presence of *AvrPm2, AvrPm3*^*b*^ and *AvrPm3*^*c*^ in the *B.g. secalis* genome, and suggested their role as host-specify determinant^46^. The ability of non-host mildews to establish micro-colonies on susceptible wheat cultivars might provide a basis for mating with adapted mildew isolates, thus giving raise to new hybrid forms with a broader host range such as Triticale powdery mildew^47^. Recent work by Praz and colleagues provided transcriptional evidence that the ability of Triticale mildew to grow on wheat and its new host triticale is based on downregulation (i.e. transcriptional removal) of numerous effectors mainly encoded within AVR gene families^38^. Therefore, loss of *Avr* function in grass mildew, combined with the deployment of susceptible wheat genotype (i.e. lacking *Pm* resistance genes), might provide a genetic basis for the raise of new adapted mildew forms. This hypothesis is substantiated by recent work by Inoue and colleagues^48^, showing that the emergence of the wheat blast pathogen is due to widespread deployment of *rwt3* wheat (i.e., lacking the *Rwt3* resistance genes), combined with the loss of function of *PWT3* (the *Avr* of *Rwt3*) in non-adapted forms of *Pyricularia oryzae* (syn. *Magnaporthe oryzae*), resulting in a host jump on wheat^48^. These findings suggest that major *R* genes such as *Pm2* and *Pm3* must be maintained in the breeding gene pool even if they are locally defeated by virulent races from the adapted wheat mildew, because they are conferring important non-host resistance preventing the emergence of new mildew pathogens.

Based on these findings, we propose that the ability of other *formae speciales* of the *tritici*-clade to grow on wheat is restricted by the presence of many *Avr-R* gene pairs. These are simultaneously involved in race-specific resistance and in host specificity, similar to more generalised models proposed by (i) Tosa ^49^, who argued that that the acquisition or loss of *Avr* genes is the main driving force behind the evolution of new *formae speciales*, a hypothesis recently substantiated by findings from wheat and rice blast^48,50^, and (ii) Schulze-Lefert and Panstruga (2011)^51,52^ suggesting that the contribution of major *R*-genes to non-host resistance inversely correlates with the phylogenetic divergence time between the analysed host and non-host plants of a certain pathogen species or *formae speciales*.

## Methods

### Fungal isolates and virulence tests

*Blumeria graminis* f. sp. *tritici* isolates were maintained in the asexual phase on detached leaves of the susceptible bread wheat (*Triticum aestivum*) cultivar Kanzler on 0.5% food grade agar (PanReac AppliChem) plates supplemented with 4.23 mM benzimidazole^53^. Virulence tests were performed on near-isogenic lines or varieties using ‘Asosan/8*Chancellor’ for *Pm3a*, ‘Chul/8xChancellor’ for *Pm3b*, ‘Sonora/8xChancellor’ for *Pm3c*, ‘Kolibri’ for *Pm3d*, ‘W150’ for *Pm3e*, ‘Michigan Amber/8xChancellor’ for *Pm3f* and the susceptible wheat cultivars ‘Kanzler’ and ‘Chancellor’ as a control^35^. Virulence scoring was performed 10 days after infection on at least three independent leaf segments. Scoring was performed qualitatively as follows: mildew leaf coverage (LC) of 60–100%, virulent; LC of 10–40%, intermediate; LC of 0%, avirulent^28^.

For the GWAS, a population of natural isolates was established by collecting mildew races from infected wheat leaves in the fields of major mildew epidemic regions in China from 2011 to 2014 described by Zeng and collaborators^54^. Single-pustule-derived isolates were purified by growing the pathogen on the susceptible wheat line ‘Chancellor,’ then multiplied and stored at −80 °C by drying spores at 23 °C for 5 h in the presence of silica gel before freezing^55^. One hundred pure isolates with diversified geographic origin and a balanced virulence/avirulence pattern on wheat differential resistance lines were manually selected from the natural population (Supplementary Data 2).

*Blumeria graminis* f. sp. *secalis* isolates S-1391 and S-1459 were maintained in the asexual phase on detached leaves of the susceptible rye (*Secale cereale*) cultivar ‘Matador’ on 0.5% food grade agar (PanReac AppliChem) plates supplemented with 4.23 mM benzimidazole^38^. To assess the influence of the *Pm3b*, *Pm3c* and *Pm3d* alleles on early stages of infection on wheat, detached primary leaves of near-isogenic wheat lines ‘Chul/8xChancellor’ for *Pm3b*, ‘Sonora/8xChancellor’ for *Pm3c*, transgenic wheat lines Pm3b#1^56^, Pm3d#1^57^ and the corresponding susceptible control ‘Chancellor’ and ‘Bobwhite’ were infected with S-1391 and S-1459 as described above for *B.g. tritici*. Leaf segments were stained 48hpi for reactive oxygen species in 1 mg ml^−1^ 3′3-diaminobenzidine (DAB)-HCl, pH3.8 solution for 12 h^58^ followed by complete de-staining of leaf pigments in ethanol:acetic acid solution (ratio 3:1) for several days. To detect fungal spores and hyphae the destained leaf segments were subsequently stained with Coomassie Brilliant Blue (0.15% in EtOH absolute), washed repeatedly in H_2_O and samples mounted for microscopy in 50% glycerol. Using a conventional bright-field microscope, spores were scored based on the following categories: (i) microcolony formation: establishment of a haustorium and production of secondary hyphae without apparent signs of hypersensitive cell-death (HR) indicating a compatible interaction in early stages of infection, (ii) penetration of epidermal cells resulting in HR and stop of growth, or early arrest of spore growth in the absence of HR. For each isolate/cultivar combination the average of eight biological replicates (=independent leaf segments) is shown. For each leaf segment at least 50 spores were assessed. The assay was repeated with similar results. Statistical significance of observed differences was tested using a Wilcoxon rank sum test for possibly tied observation (wilcox.exact function, R package exactRankTests).

### Haplotype mining

Haplotype diversity was assessed on a subset of the worldwide *B. graminis* f. sp. *tritici* collection previously described by McNally and colleagues^29^. For the isolates originating from the United States, Israel, and Europe the *AvrPm3*^*b2/c2*^ and *AvrPm3*^*d3*^ genes were PCR amplified from genomic DNA with primers listed in Supplementary Table 1, and Sanger-sequenced. All other isolates, including *B.g. secalis* and *B.g. dactylidis* accessions, were mined for the same two genes using genome sequence data^13,30,47^. Coding sequences of *AvrPm3*^*b2/c2*^ variants in *B.g. tritici*, *B.g. secalis* and *B.g. dactylidis* found in this study are available under GenBank accession numbers MK806469-MK806484. Estimates for pN/pS ratio^59^ of *AvrPm3*^*b2/c2*^ haplotypes were calculated using the DnaSP 6 software package with default parameters^60^. Estimation of copy number variation of *AvrPm3*^*b2/c2*^, and *AvrPm3*^*d*3^ and its paralog *BgtE-20069a* was visualised based on a previously published analysis of gene coverage in 36 wheat powdery mildew genomes by Müller and colleagues^34^.

### DNA/RNA isolation

Fungal high molecular weight DNA was extracted using an optimised procedure described in detail by Bourras and colleagues^28,30^. In short, ~100 mg of conidia were frozen in liquid nitrogen and ground three times 30 s using stainless steel beads and a high-speed plate grinder (MM200 Mixer Mill, Retsch, Germany) at a grinding frequency of 30 s^−1^. To the ground conidia, 300 μl of 65 °C preheated 5% Sarcosyl solution was added and vigorously vortexed. After addition of 700 μl of CTAB-Buffer (0.2 M Tris(hydroxymethyl)aminomethane at pH 7.5, 50 mM EDTA, 2 M NaCl, 2% Cetyl trimethylammonium bromide (CTAB) and 0.25 M Sodium metabisulfite (Na2S2O5)) the samples were incubated at 65 °C for 30 min. After incubation, 600 μl Chloroform was added, followed by 10 min of centrifugation at 14,000 rcf, 4 °C. The supernatant was collected and combined with one volume of −20 °C precooled Isopropanol, mixed by careful inversion and centrifuged for 10 min, 14,000 rcf, 4 °C. The resulting pellet was dried on ice and resuspended in standard TrisEDTA buffer before further purification on AmiconUltra 0.5 ml centrifugal filters MWCO 30 kDa (Sigma-Aldrich, Steinheim, Germany).

RNA samples were extracted from infected wheat leaves of the susceptible cultivar ‘Chinese Spring’ using the SV Total RNA Isolation System (Promega) according to the manufacturer’s protocol. RNA integrity was assessed by gel electrophoresis and spectrophotometric analysis using a NanoDrop 1000 Sepctrophotometer (ThermoScientific)^38^. RACE-PCR was performed using the SMARTer RACE cDNA Amplification Kit (Clontech Laboratories Inc.) according to the manufacturer and with gene-specific primers listed in Supplementary Data 4.

### Codon optimisation, gene synthesis and plasmids

Codon optimisation of effector coding sequences for expression in *N. benthamiana* was performed using the online tool provided by Integrated DNA technologies (https://eu.idtdna.com/CodonOpt). In all cases the signal peptide, as predicted by the SignalP4 algorithm^61^ (http://www.cbs.dtu.dk/services/SignalP/) was replaced by an ATG start codon. Gene synthesis including gateway-compatible attL cloning sites was performed with commercial partners: Gen9 (https://www.gen9bio.com), BioCat (https://www.biocat.com), Invitrogen (https://www.thermofisher.com) and Twist Bioscience (https://twistbioscience.com). The synthesised genes were cloned into the *A. tumefaciens* expression vector pIPKb004^62^ using Gateway LR clonase II (Invitrogen) according to the manufacturer and subsequently transformed into *A. tumefaciens* strain GV3101 using electroporation (1.44 kV, 25 μF, 200Ω)^28^.

Site-directed mutagenesis (SDM) was performed by PCR amplification on primary gene-synthesis products with non-overlapping primers (Supplementary Table 2). Linearised SDM products were phosphorylated using T4 polynucleotide kinase (New England Biolabs) and re-ligated with T4 DNA Ligase (New England Biolabs) according to the manufacturer. Epitope tagged Pm3 alleles have been published by Brunner and colleagues^56^. Epitope tagging of effector genes has been performed by gene-synthesis or SDM as described above (see also Supplementary Note 3). A complete list of all synthesised or mutagenized (SDM) constructs including nucleotide sequence and manufacturer can be found in Supplementary Data 3.

### BAC sequencing

The *B.g. tritici* BAC library has been previously produced from the reference isolate Bgt_96224 and is described in^53^. BACs were selected based on their positions in BAC fingerprint assemblies generated with LTC^63^ and manually combined into a scaffold (Supplementary Fig. 2, Supplementary Table 3). Candidate clones were confirmed by PCR and plasmids were extracted using the Qiagen Large-Construct Kit according to the manufacturer’s protocol. BAC insert sizes were estimated by digestion with NotI and pulsed field gel electrophoresis. Selected BAC clones were sequenced using Illumina MiSeq technology (2 × 250 bp paired end; GATC Biotech). Individual BAC reads were assembled using the CLC Genomics Workbench version 6.0.1 with default settings.

### RNA-seq data analysis

Gene expression data at 2 dpi on the susceptible cultivar ‘Chinese Spring’ for the *B.g. tritici* isolates Bgt_96224, _94202, and _JIW2 was obtained from the study of Praz and colleagues^38^ available at Gene Expression Omnibus (GEO) under accession number GSE108405 [https://www.ncbi.nlm.nih.gov/gds/?term=GSE108405]. Reads per kilo base pairs per million reads (rpkm) values were obtained with edgeR^64^ and used for the effector benchmarking analysis. Normalisation was done with edgeR using the command calcNormFactors(method = ”TMM”). Genes were considered as expressed if the cpm (count per million) was >10 in at least three of the nine replicates. Genes were considered as differentially expressed if the log2 fold change (log2FC) value obtained with edgeR was >1. Statistical analysis was performed with edgeR using the following sequence of commands: estimateGLMCommonDisp(), estimateGLMTrendedDisp(), estimateGLMTagwiseDisp(), glmFit() and glmLRT(). RNA-Seq data of three *B.g. tritici* and two *B.g. secalis* isolates (obtained from GSE108405, three replicates each) were also mapped on the new PacBio reference genome assembly (v3.16^34^) using STAR^65^ with the following parameters:–outFilterMultimapNmax 10–outFiltermismatchNoverLmax 0.02–alignIntronMax 500. We obtained expression counts for each gene with the most recent gene annotation from Müller and colleagues^34^ using Salmon with standard parameters^66^.

### Genome-wide association study

Fungal isolates used for GWAS are described in Supplementary Data 2. Genomic sequences are available under the accession number SRP062198 [https://www.ncbi.nlm.nih.gov/sra/?term = SRP062198] (from^30^ and this study). Illumina reads were mapped on the reference genome (v3.16) using Bowtie2 with parameters ‐L,‐0.6,‐0.25^67^. The resulting Bam files were processed with the SAMtools view and sort commands^68^. Duplicated reads were removed with the SAMtools command rmdup. Picard Tools was used to add read groups to the bam files (http://broadinstitute.github.io/picard). Bam files were merged using bamtools merge command and indexed using samtools index command. SNP calling was done using freebayes^69^ with the following parameters: -p 1 -m 30 -q 20 -z 0.03 -F 0.7–3 200–genotype-qualities. VCF file was subsequently filtered using vcftools with the following parameters:–maf 0.05–max-meanDP 40–remove-indels–min-alleles 2–max-alleles 2–minGQ 30–max-missing 0.9. VCF file was transformed into hapmap format using a custom perl script. GWAS was performed using the GAPIT package for R^33^ using the the PCA.total = 3 option.

### Transient assays in Nicotiana benthamiana and HR measurement

*A. tumefaciens* mediated transient expression of *AvrPm3* candidates, *SvrPm3* and *Pm3* alleles in *N. benthamiana* was achieved as follows: *Agrobacteria* were grown in Luria broth (LB) medium supplemented with appropriate antibiotics overnight at 28 °C with 200 rpm shaking. Fully grown cultures were harvested by centrifugation 3300 × *g*, 5 min, resuspended in fresh LB medium without antibiotics and further incubated for 30 min at 28 °C with 200 rpm shaking. Bacteria were harvested again by centrifugation at 3300×*g* for 5 min and the pellet was resuspended and diluted in AS-medium (10 mM MES-KOH, pH5.6; 10 mM MgCl_2_; 200 µM acetosyringone) to an OD of 1.2 before incubation for 3 to 4 h at 28 °C with 200 rpm shaking to induce virulence^28^. Before infiltration into *N. benthamiana, Agrobacteria* expressing *AvrPm3* candidates, *SvrPm3* or *Pm3* alleles were mixed in the indicated ratios.

HR was assessed visually and by fluorescence scanning using the Fusion FX Imaging System (Vilber Lourmat, Eberhardzell, Germany) with the following pre-settings: fluorescence sample; excitation: blue epi-illumintaion; emission: filter F-535 Y2; aperture: 0.84-open; sensitivity: full resolution^30^. The time-point of HR assessment was chosen depending on the experimental setup: 5 days after *Agrobacterium* infiltration for verification of AVR recognition by the corresponding R-gene, 2dpi for suppression assays involving *SvrPm3* and to test for inter-allelic suppression. HR was quantified using high-resolution epi-fluorescence images obtained with the FusionFX HRS technology, and analysed with the ImageJ image analysis software measuring the integrated density of fluorescence^29,30^. HR values were normalised to the infiltrated leaf area and to the control. Statistical significance was assessed with the Student’s *t-*test for paired data (*p* < 0.05).

### Protein detection

The soluble protein fraction was extracted from *A.tumefaciens* infiltrated *N. benthamiana* leaves 2 days after infiltration. Two leaf discs (0.5 mm diameter) from 4 infiltrated leaves were pooled, ground in liquid nitrogen and resuspended in 2x modified Laemmli-Buffer (100 mM Tris-HCl pH 6.8, 200 mM DTT, 0.04% bromphenol blue, 20% glycerol, 2% SDS), boiled for 5 min and centrifuged at 10,000 × *g* for 10 min at 4 °C. Ten microlitre of total protein extract was separated in SDS polyacrylamide (PA) gels (8% PA for PM3-proteins, 16% PA for effectors) and semi-dry blotted to a nitrocellulose membrane (Amersham Protran 0.2 µm NC) using a Trans-Blot SD Semi-Dry Transfer Cell (Bio-Rad). Blotted protein was stained with Ponceau S. For HA detection, a peroxidase conjugated antibody (anti-HA-HRP, rat monoclonal, clone 3F10, Roche) was used at a dilution of 1:3000. For FLAG detection the primary antibody (anti-FLAG, mouse monoclonal, clone M2, Sigma-Aldrich) was used at a dilution of 1:10,000, membranes washed with TBST and incubated with anti-mouse peroxidase antibody (anti-mouse-HRP, goat polyclonal, Sigma Aldrich) diluted 1:4000. Peroxidase chemiluminescence was detected using a Fusion FX Imaging System (Vilber Lourmat, Eberhardzell, Germany) and WesternBright ECL HRP substrate (Advansta).

### Phylogenetic analysis and structural modelling

Protein sequences of the AVRPM3^A2/F2^, AVRPM3^B2/C2^, AVRPM3^D3^, SVRPM3^A1/F1^ and the E005 effector families were obtained from Müller and colleagues^34^. Protein sequence alignments were performed using the MUSCLE v3.8 available online (https://www.ebi.ac.uk/Tools/msa/muscle/), assessment of phylogenetic relationships, and construction/depiction of the phylogenetic tree was performed using CLC workbench software (v7.9.1). We used the following parameters for multiple sequence alignments in CLC: gap open cost = 20.0, gap extension cost = 2.0, end gap cost = as any other, alignment = very accurate. We used the following parameters for tree construction: tree construction method = UPGMA, protein distance measure = Jukes-Cantor, bootstrapping replicates = 100. Phylogenetic trees were depicted as circular phylograms with a threshold for bootstrap value >80, using CLC workbench (v7.9.1). Prediction of secondary protein folds was done with Quick2D (https://toolkit.tuebingen.mpg.de/#/tools/quick2d) an integrative prediction tool from the MPI Bioinformatics Toolkit^36^. Briefly, sequences of the AVRPM3 proteins were submitted without the signal peptide, and secondary structure modelling was done with the recommended default parameters (database for MSA generation = nr90; maximal number of MSA generation steps = 3; threshold E-value = 1e-3). The Quick2D modelling employs four different algorithms for prediction, namely a PSI-blast based secondary structure prediction algorithm (PSIPRED), an iterative deep neural network (DNN) based algorithm (SPIDER2), a multiple backpropagation neural network predictor (PSSPRED), and a deep convolutional neural fields based method (DEEPCNF). Secondary folds depictions were extracted and adapted from the Quick2D output. Three dimensional protein modelling was performed using the RaptorX protein modelling tool (http://raptorx.uchicago.edu/). Protein sequences corresponding to the predicted mature protein after cleavage of the predicted signal peptide were modelled. The resulting predicted folds were manually inspected and visualised in order to identify possible common structures. For each predicted fold, the *P*-value, the Global Distance Test (GDT) score, and uSeqID identity score were recorded (Supplementary Data 5). Detailed definitions of these score values are available in the RaptorX user documentation (http://raptorx.uchicago.edu/documentation/).

### Reporting summary

Further information on research design is available in the Nature Research Reporting Summary linked to this article.

## Supplementary information

Supplementary Information

Description of Additional Supplementary Files

Supplementary Data 1

Supplementary Data 2

Supplementary Data 3

Supplementary Data 4

Supplementary Data 5

Reporting Summary

Source Data

## Data Availability

Gene expression data was obtained from Gene Expression Omnibus (GEO) under accession number GSE108405 [https://www.ncbi.nlm.nih.gov/gds/?term = GSE108405]. Genomic sequences of the fungal isolates used for GWAS are available under the accession number SRP062198 [https://www.ncbi.nlm.nih.gov/sra/?term = SRP062198]. Coding sequences of naturally occurring variants of *AvrPm3*^*b2/c2*^ were deposited under Genbank accession numbers MK806469-MK806484 [https://www.ncbi.nlm.nih.gov/nuccore/?term = MK806469]. Individual gene and protein sequences used in this study are provided in Supplementary Data 3. The source data underlying Figs. 1(d, h), 2(a, b), 3(a–g), 4(a, c), 5(c–e), and 6(a–e) are provided as Source Data file. The source date underlying Supplementary Figs. 6(a–d), 7(a–d), 8(a, b), 9(a–e), 15, 18, 19(b), 20(c), 21(b), 22(d, e), 23(h–k), and 25(b, c) are provided as Source Data file. Any additional data or biological material that support the findings of this study are available from the corresponding author upon reasonable request.
