# Supplementary material for: The AvrPm3-Pm3 effector-NLR interactions control both race-specific resistance and host-specificity of cereal mildews on wheat
Source: Nat Commun. 2019 May 23;10:2292. doi: 10.1038/s41467-019-10274-1 (PMC6533294; doi:10.1038/s41467-019-10274-1)
Supplement: Supplementary file 1 — Supplementary Information [file 41467_2019_10274_MOESM1_ESM.pdf]

Supplementary Information

The *AvrPm3-Pm3* effector-NLR interactions control both race-specific resistance and host-specificity of cereal mildews on wheat.

Bourras, Kunz, Xue et al. (2019)

Supplementary Figures

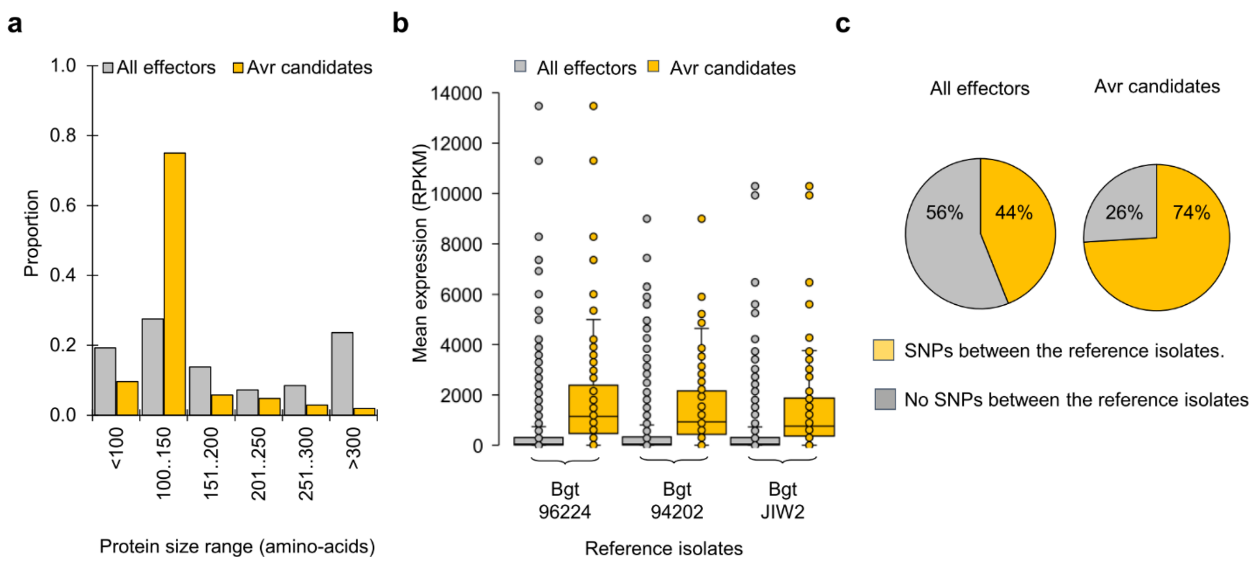

Supplementary Figure 1. Assessment of *Avr* gene features in the subset of 100 candidates selected by effector benchmarking. Candidate *Avrs* were characterized for the size of the encoded protein **(a)**, gene expression levels in the reference mildew isolate Bgt_96224, Bgt_94202 and Bgt_JIW2 **(b)**, and single nucleotide polymorphisms (SNPs) among the three mildew reference isolates Bgt_96224, Bgt_94202 and Bgt_JIW2 **(c)**. In (b), gene expression values are given as Reads Per Kilobase Million (RPKM). Mean values are indicated by the middle line in the boxplot. Individual data points are plotted along the whisker lines. Data points outside the whisker boundaries represent outliers outside the inner quartile range. In (c) synonymous and non-synonymous SNP mutations were not distinguished (Supplementary Note 1). Source data is provided in Supplementary Data 1.

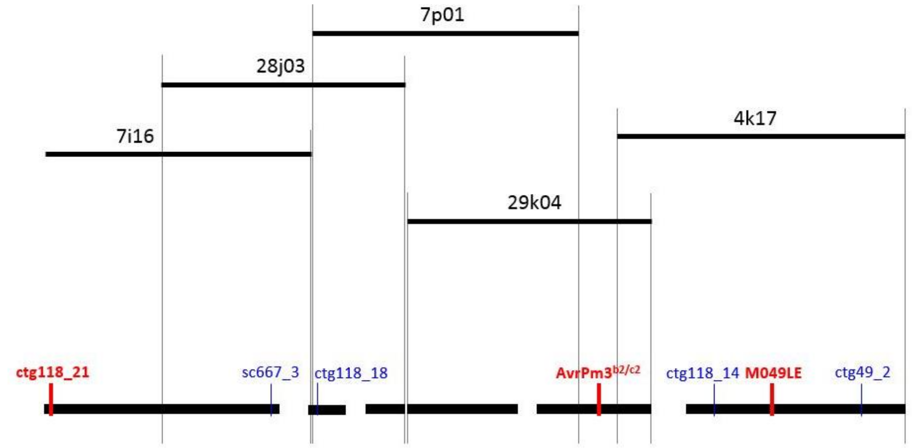

Supplementary Figure 2. Bacterial artificial chromosome (BAC) clones spanning the $AvrPm3^{b2/c2}$ genetic *Locus_3*. The minimal tiling path used to reconstruct *Locus_3* consists of five BACs spanning a physical interval of 371kb. BAC identifiers are indicated in black, genetic markers in this region are depicted in blue. The position of the *Locus_3* flanking markers ctg118_21 and M049LE as well as the position of $AvrPm3^{b2/c2}$ (*BgtE-20002*) are marked in red. Marker M049LE has previously been described [1]. Additional marker information can be found in Supplementary Table 3.

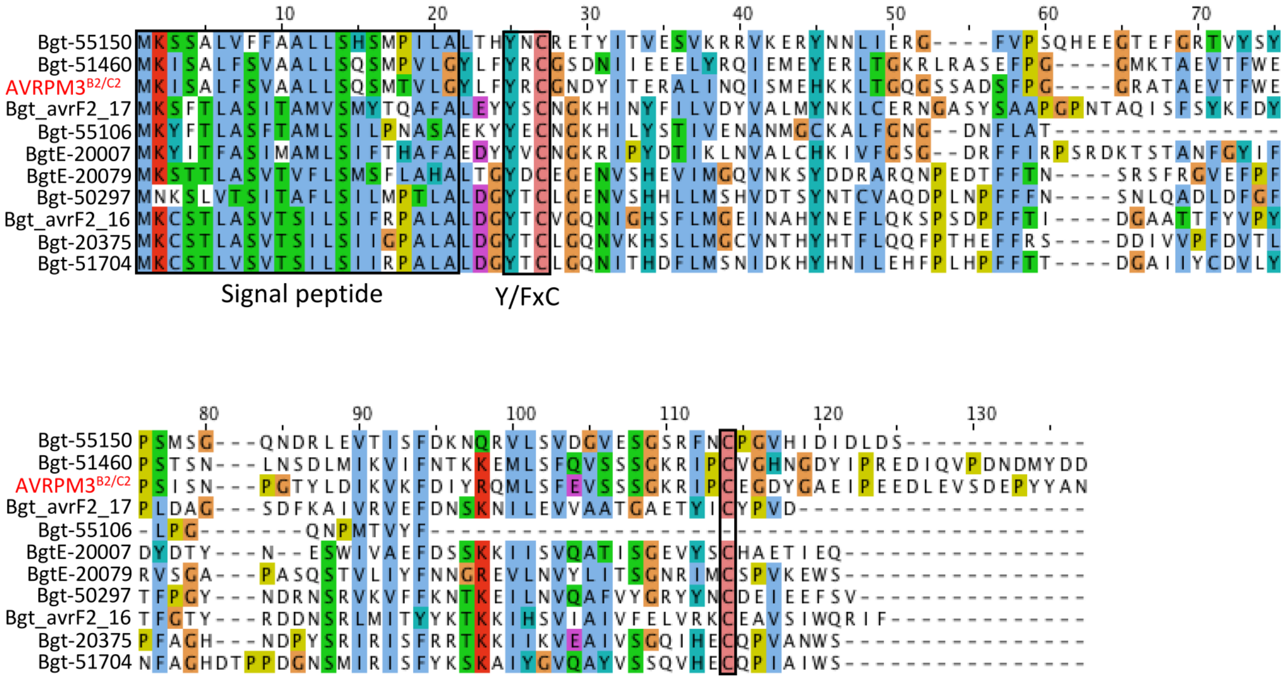

Supplementary Figure 3. Protein sequence alignments of the effector gene family E018 members found in the reference isolate Bgt_96224. AVRPM3$^{B2/C2}$ is highlighted (red). The N-terminal predicted signal peptide, the conserved Y/FxC motif and C-terminal cysteine are indicated (black boxes). Conserved amino acids are colored based on biochemical or physical properties (ClustalX color scheme: blue: hydrophobic; red: positively charged; purple: negatively charged; green: polar uncharged; yellow: proline; pink: cysteine; orange: glycine).

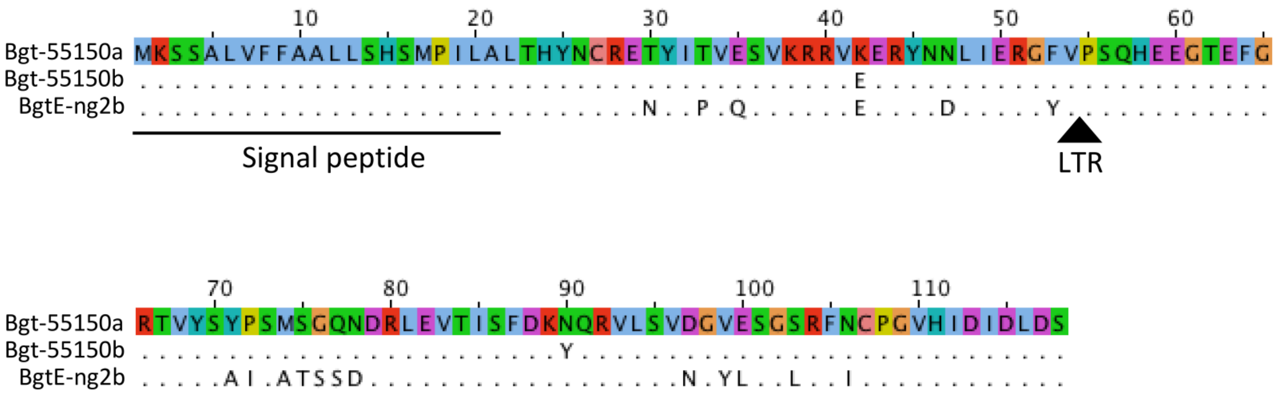

Supplementary Figure 4. Protein sequence alignment of the AVR candidates from *Locus_3* exhibiting polymorphisms between the reference isolates Bgt_96224 and Bgt_94202. The sequence of Bgt-55150a originating from Bgt_96224 is shown as a reference, for Bgt-55150b and BgtE-ng2b, both found in Bgt_94202, only polymorphic residues are indicated whereas identical residues are represented by dots. BgtE-ng2 is found to be disrupted by the insertion of a partial LTR-retrotransposon sequence (607bp) in Bgt_96224, the insertion site is indicated by a black triangle. The extent of the N-terminal predicted signal peptide is highlighted by a black line. Coloring of the amino acids is based on biochemical or physical properties (ClustalX color scheme: blue: hydrophobic; red: positively charged; purple: negatively charged; green: polar uncharged; yellow: proline; pink: cysteine; orange: glycine).

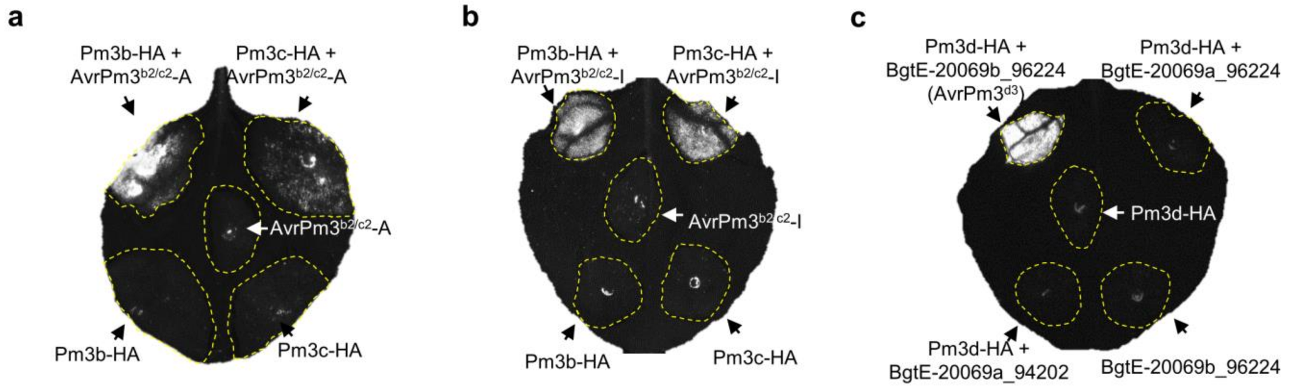

Supplementary Figure 5. *AvrPm3$^{b2/c2}$* and *AvrPm3^{d3}* functional validation assays: auto-activity controls. **(a-b)** R gene auto-activity controls for *Pm3b* and *Pm3c* in set-ups where these were individually expressed or combined with **(a)** AvrPm3$^{b2/c2}$-A, or **(b)** AvrPm3$^{b2/c2}$-I. **(c)** NLR auto-activity controls for Pm3d in a set-up where it is individually expressed or combined with BgtE-20069b_96224, BgtE20069a_96224, and BgtE_20069a_94202. HR was assessed using HSR imaging 5 days after *Agrobacterium* infiltration. Results were consistent over at least two independent assays of 6 to 8 independent leaf replicates**.**

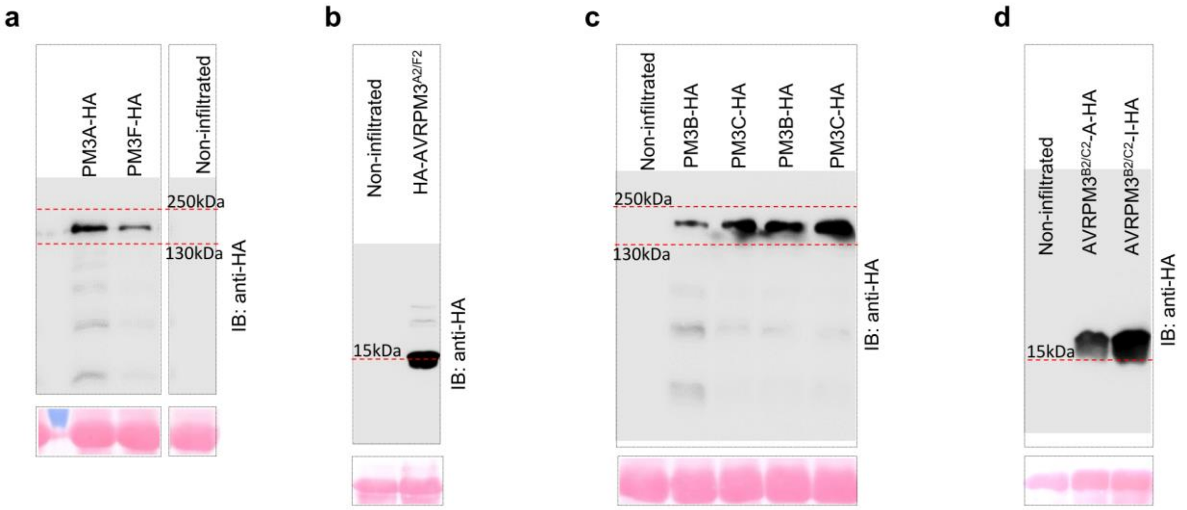

Supplementary Figure 6. Western blot detection of AVRPM3 and PM3 proteins.
(a-d) Western blot detection of epitope tagged NLR and AVR proteins from Fig. 2d-f. **(a)** Western blot detection of PM3A-HA and PM3F-HA from Fig. 2d. **(b)** Western blot detection of HA-AVRPM3$^{A2/F2}$ from Fig. 2d. **(c)** Western blot detection of PM3B-HA and PM3C-HA from Fig. 2e (two first bands to the left) and Fig. 2f (two last bands to the right). **(d)** Western blot detection of AVRPM3$^{B2/C2}$-A-HA from Fig. 2e and AVRPM3$^{B2/C2}$-I-HA from Fig. 2f. Ponceau staining of the Western blot membrane is depicted in the lower panels from a-d. Uncropped Western blot images are provided in a Source Data File.

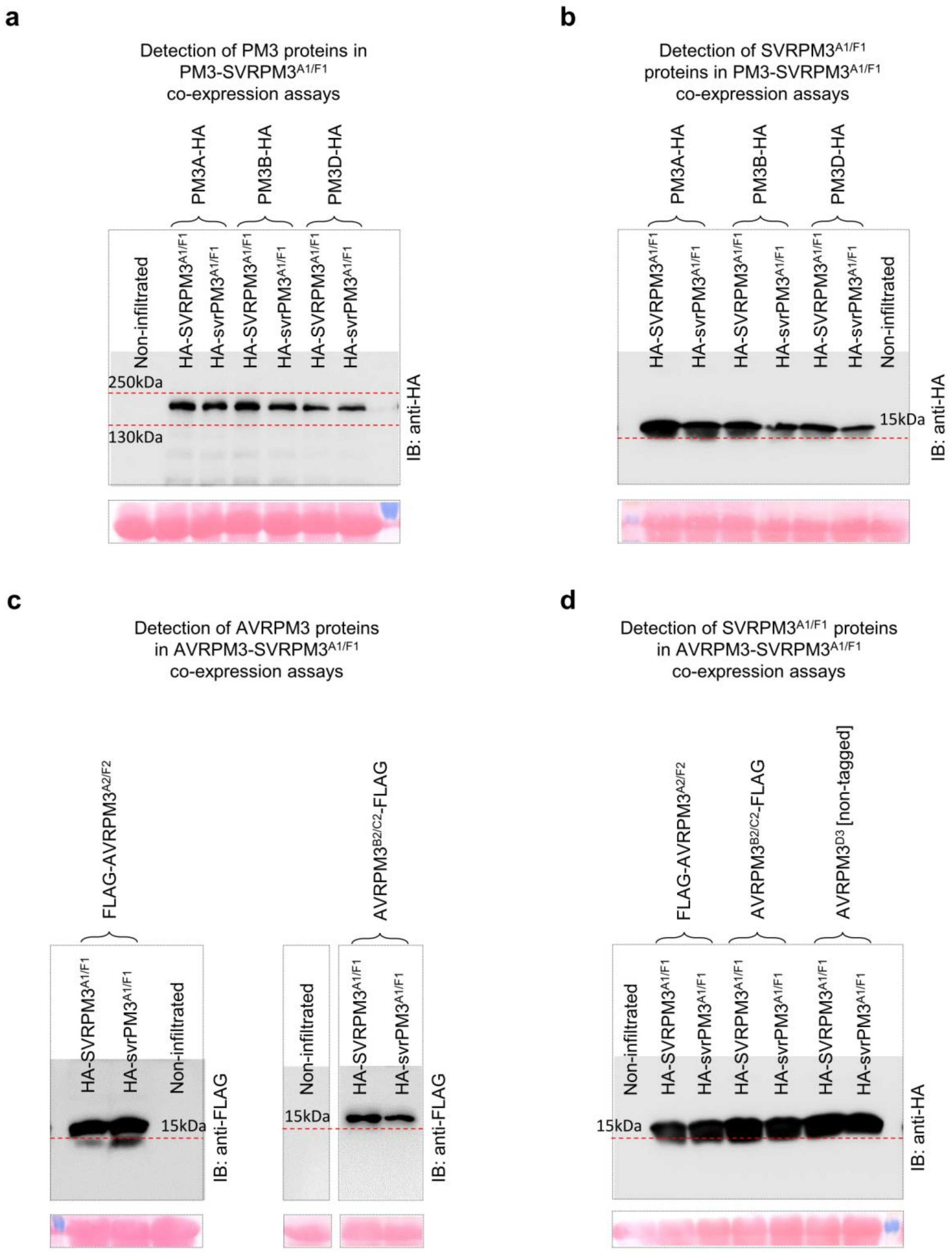

Supplementary Figure 7. Assessment of the effect of SVRPM3$^{A1/F1}$ expression in *Nicotiana benthamiana* on PM3 and AVRPM3 protein levels.

(a) Western blot detection of HA epitope tagged fusion of the PM3A, PM3B, and PM3D proteins in presence of the active HA-SVRPM3$^{A1/F1}$ or the inactive HA-svrPM3$^{A1/F1}$ variants. **(b)** Western blot detection of HA-SVRPM3$^{A1/F1}$ and HA-svrPM3$^{A1/F1}$ from the same samples as in (a). Based on (a) and (b) we conclude that the mode of action of SVRPM3$^{A1/F1}$ is not based on suppression of PM3 protein expression. **(c)** Western blot detection of FLAG-AVRPM3$^{A2/F2}$ and AVRPM3$^{B2/C2}$-FLAG in presence of the active HA-SVRPM3$^{A1/F1}$ and

the inactive HA-svrPM3$^{A1/F1}$ variants. **(d)** Western blot detection of HA-SVRPM3$^{A1/F1}$ and HA-svrPM3$^{A1/F1}$ from the same samples in (c) (first 4 bands from the left) and from one additional sample where both SVR variants were co-expressed with non-tagged AVRPM3^{D3} (last 2 bands on the right). Based on (c) and (d) we conclude that the mode of action of SVRPM3$^{A1/F1}$ is not based on suppression of AVRPM3 protein expression. Uncropped Western blot images are provided in a Source Data File.

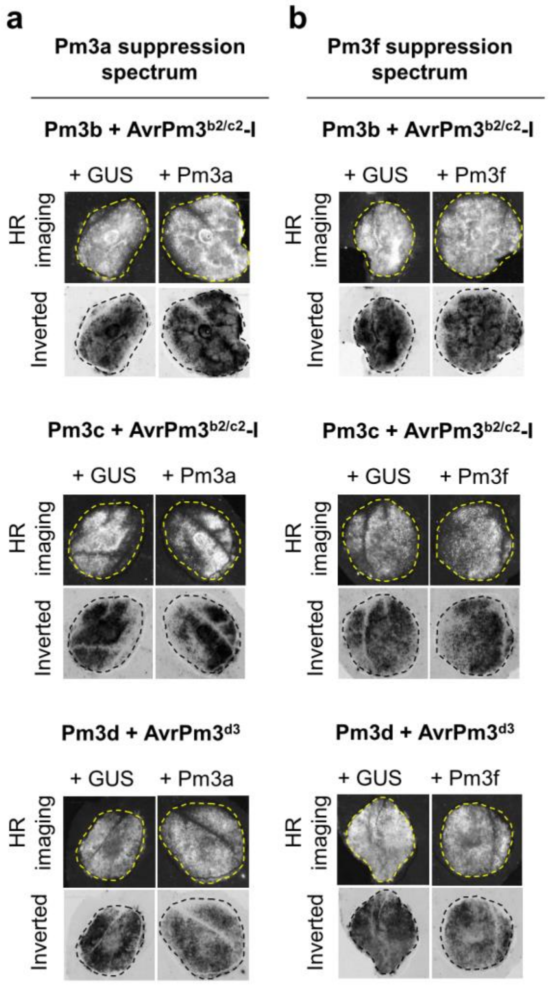

Supplementary Figure 8. *Pm3a* and *Pm3f* show no suppression activity towards *Pm3b*, *Pm3c* and *Pm3d*. The suppression spectra of *Pm3a* **(a)** and *Pm3f* **(b)** were assessed for every *AvrPm3*-*Pm3* pair in presence of other *Pm3* alleles as compared to a control where *Pm3a* and *Pm3f* are replaced by GUS. HR was assessed using HSR imaging 5 days after agrobacterium infiltration. Results are consistent over at least two independent assays of 6 to 8 independent leaf replicates each. Complete *N. benthamiana* leaf pictures are provided in a Source Data File.

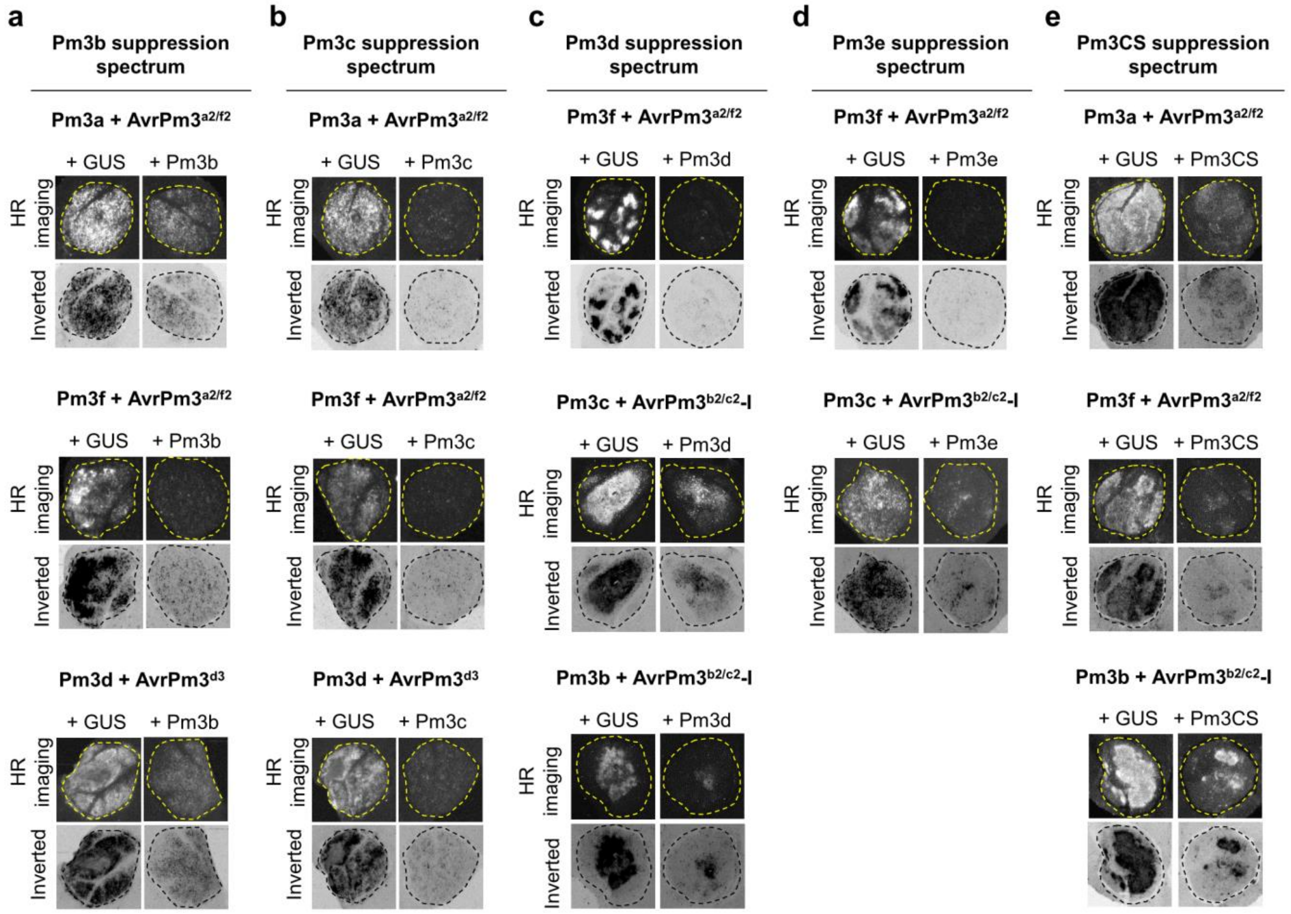

Supplementary Figure 9. Suppression of *AvrPm3-Pm3* recognition by *Pm3* NLR interactions. The suppression spectra of *Pm3b* **(a)**, *Pm3c* **(b)**, *Pm3d* **(c)**, and *Pm3e* **(d)** and *Pm3CS* **(e)** are depicted. Suppression is assessed for every *AvrPm3-Pm3* pair in presence of other *Pm3* alleles as compared to a control where the suppressor NLR is replaced by GUS. HR was assessed using HSR imaging 5 days after agrobacterium infiltration. Results are consistent over at least two independent assays of 6 to 8 independent leaf replicates each. Complete *N. benthamiana* leaf pictures are provided in a Source Data File.

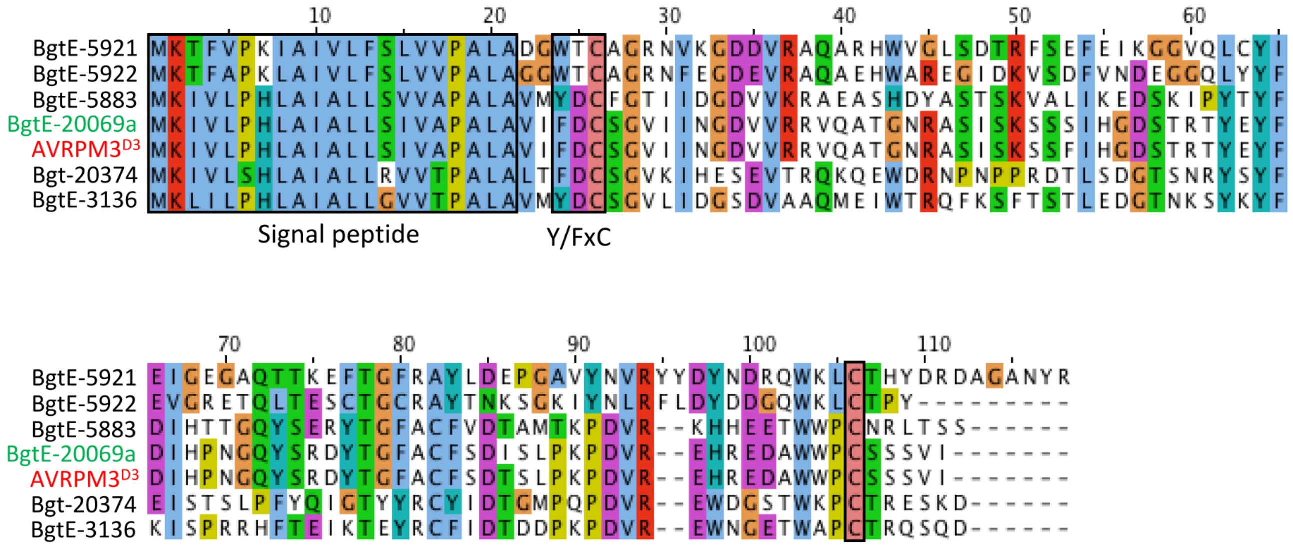

Supplementary Figure 10. Protein sequence alignments of the effector gene family E034 members found in the reference isolate Bgt_96224.

AVRPM3^{D3} (red) and its duplication BgtE-20069a (green) are highlighted. The N-terminal predicted signal peptide, the conserved Y/FxC motif and C-terminal cysteine are indicated (black boxes). Conserved amino acids are colored based on biochemical or physical properties (clustal x color scheme: blue, hydrophobic; red, positively charged; purple, negatively charged; green, polar uncharged; yellow, proline; pink, cysteine; orange, glycine).

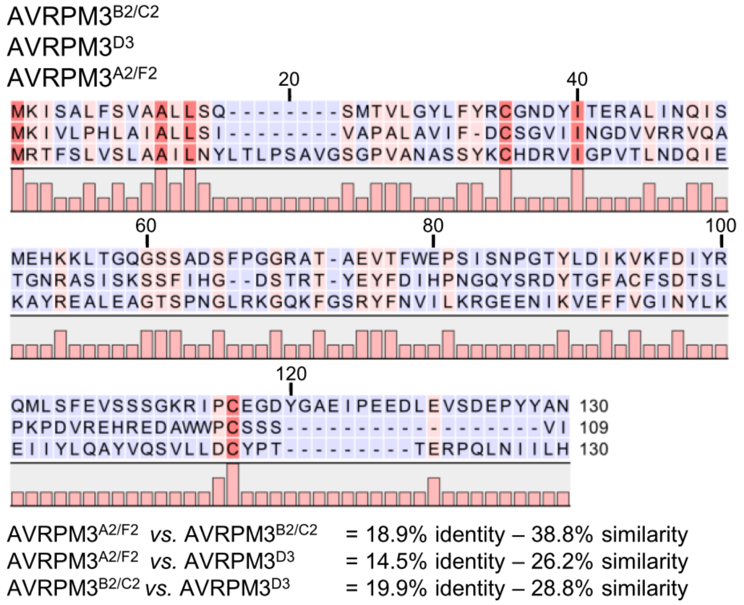

Supplementary Figure 11. Protein alignment of AVRPM3$^{A2/F2}$, AVRPM3$^{B2/C2}$, and AVRPM3^{D3}.

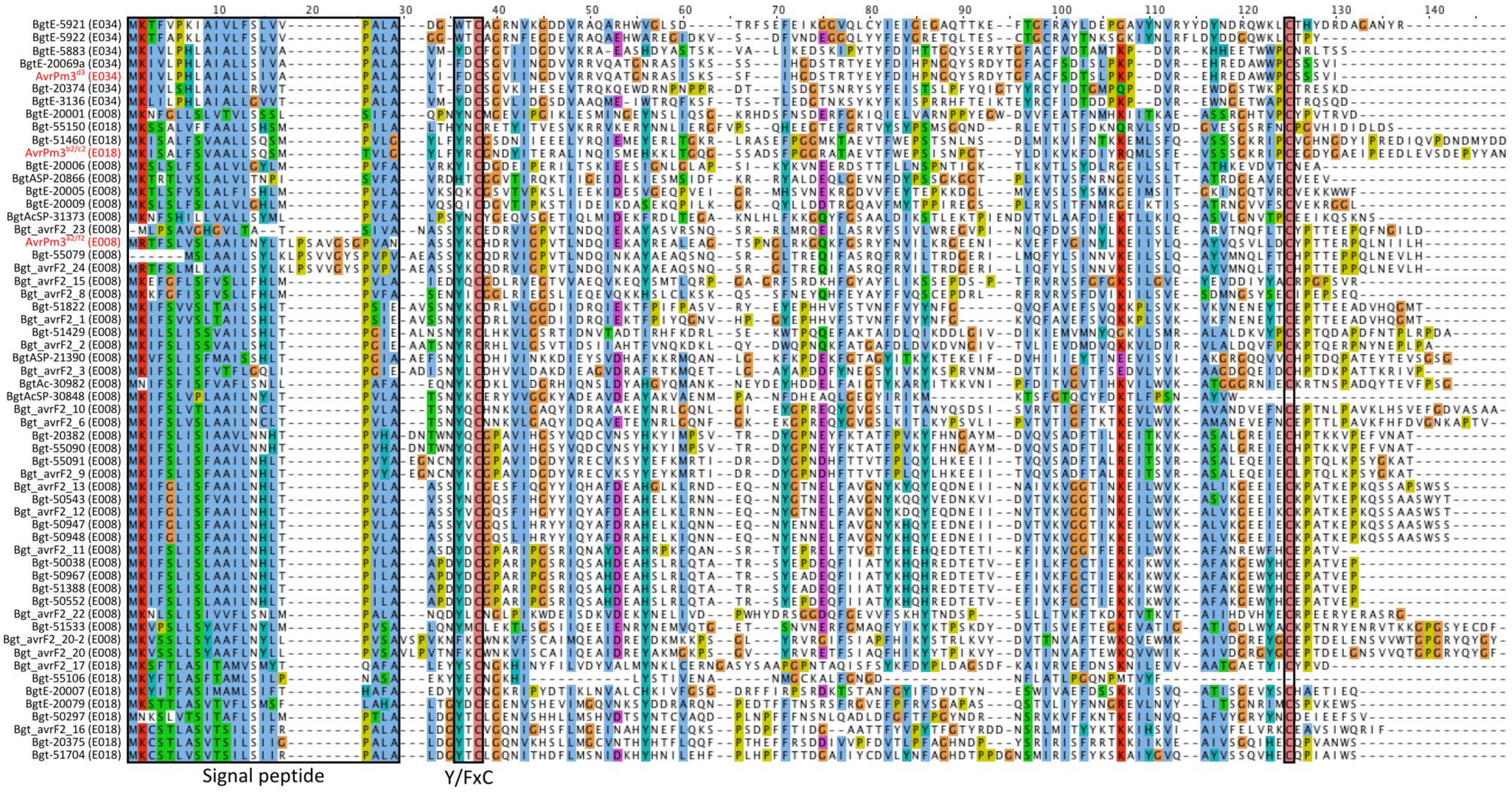

Supplementary Figure 12. Protein sequence alignments of the effector gene families E008, E018 and E034 found in the reference isolate Bgt_96224. AVRPM3$^{A2/F2}$, AVRPM3$^{B2/C2}$ and AVRPM3^{D3} are highlighted (red) and the corresponding families are indicated in brackets. The N-terminal predicted signal peptide, the conserved Y/FxC motif and C-terminal cysteine are indicated (black boxes). Conserved amino acids are colored based on biochemical or physical properties (clustal x color scheme: blue, hydrophobic; red, positively charged; purple, negatively charged; green, polar uncharged; yellow, proline; pink, cysteine; orange, glycine).

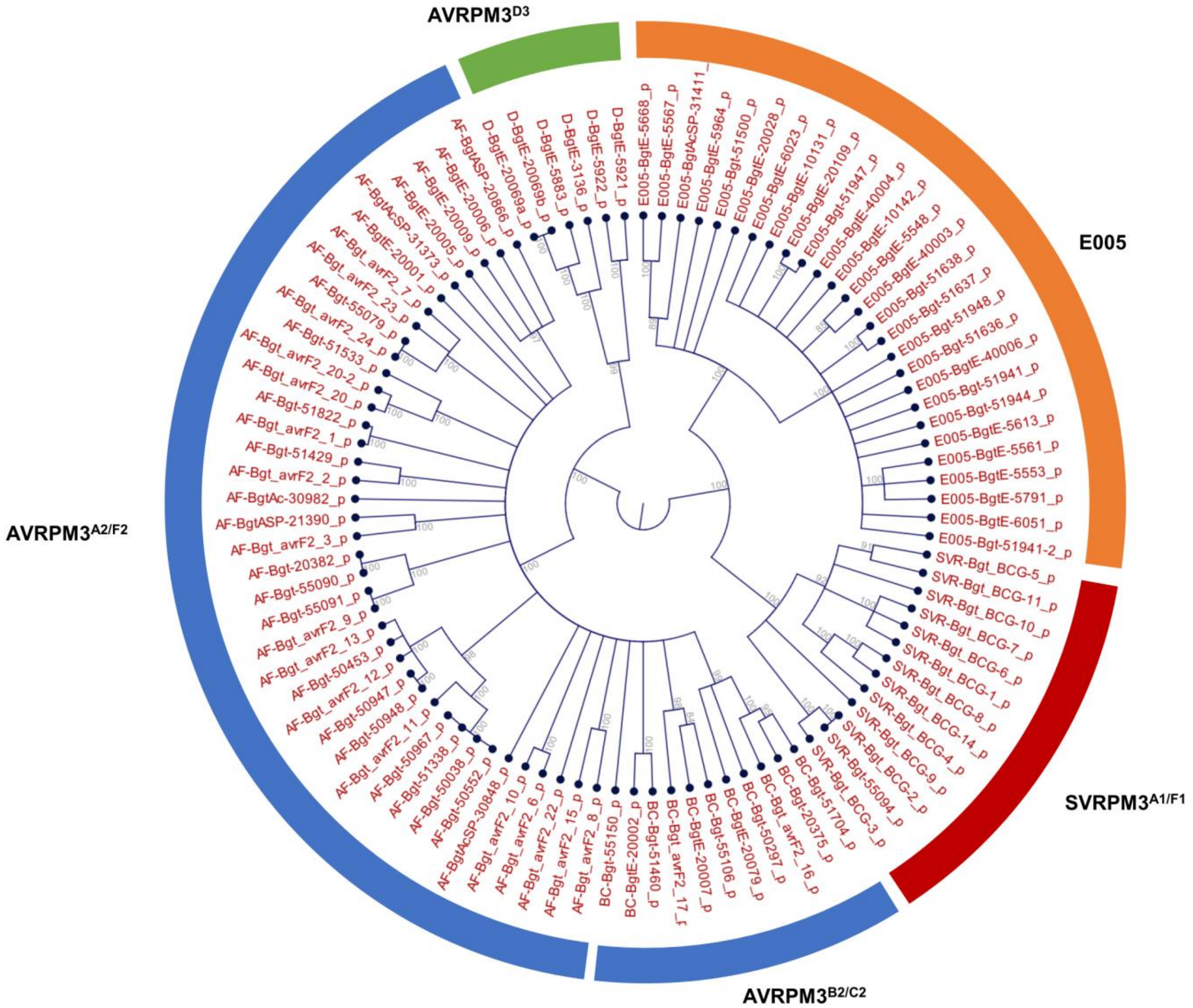

Supplementary Figure 13. High resolution depiction of the phylogenetic relationships among AVR and SVR effector protein families in *B.g. tritici*.

The effector families of AVRPM3$^{A2/F2}$ and AVRPM3$^{B2/C2}$ (blue segment), AVRPM3^{D3} (green segment) are indicated. The SVRPM3$^{A1/F1}$ (red segment) and E005 (orange segment) were used as outgroups. Wheat powdery mildew effector gene families were previously defined based on BLAST analysis [2]. The depicted phylogenetic analysis suggests the AVRPM3$^{A2/F2}$ and AVRPM3$^{B2/C2}$ families are not phylogenetically separate, but form one contiguous group of effectors that have probably evolved from a common ancestor. Only branches with a minimum bootstrap value of ‘80’ are depicted (see Methods).

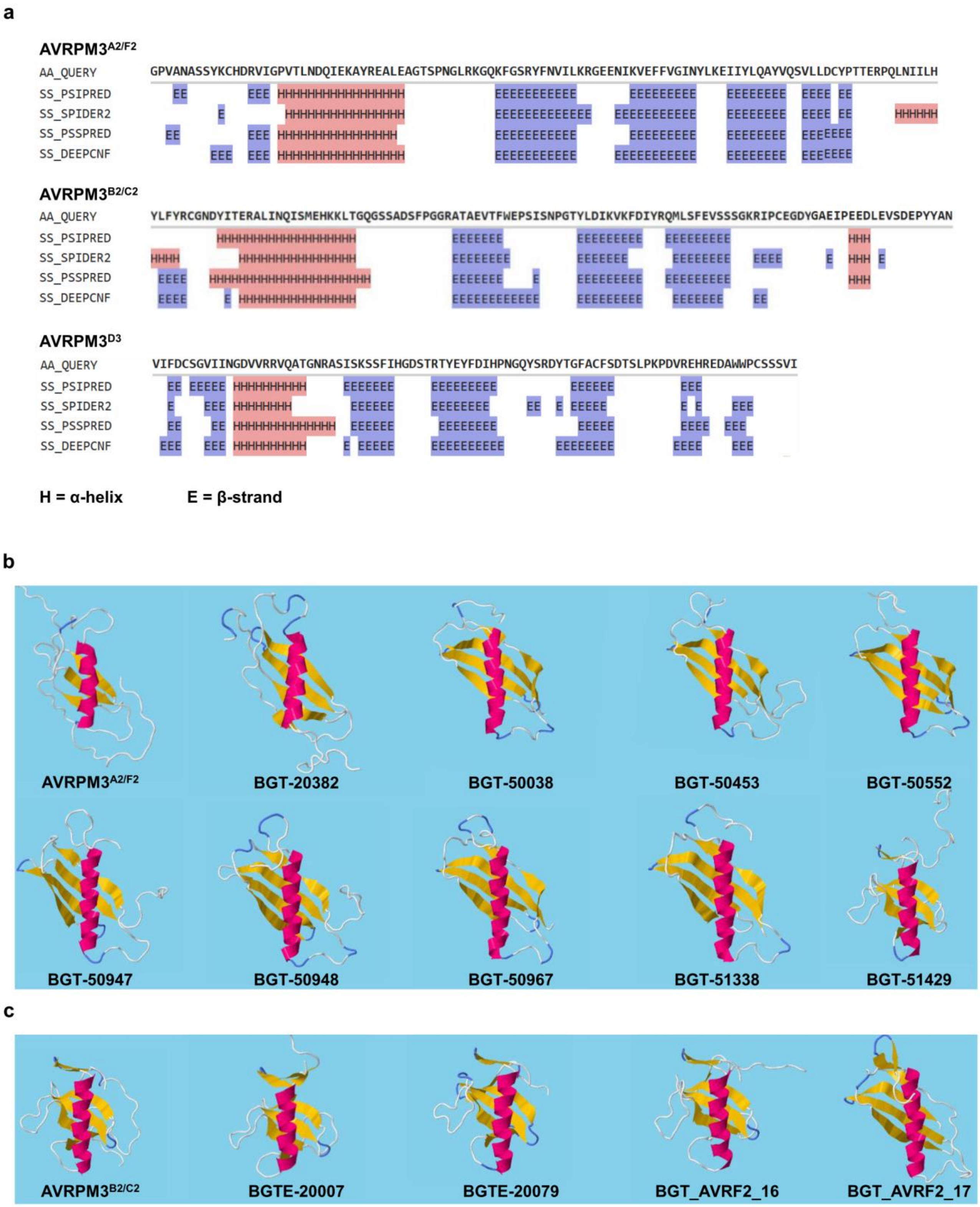

Supplementary Figure 14. *In silico* structural modelling of secondary and tertiary folds of the AVRPM3 proteins.

(a) Secondary fold prediction for AVRPM3$^{A2/F2}$, AVRPM3$^{B2/C2}$, and AVRPM3^{D3} using the Quick2D tool from the Max Planck Institute Bioinformatics Toolkit [3]. Output from four different secondary structure prediction

methods (i.e. PSIPRED, SPIDER2, PSSPred, and DEEPCNF) are depicted. Predicted secondary folds consistently consisted of one alpha helix (H) and at least 3 to 4 beta-strands (E). See (https://toolkit.tuebingen.mpg.de/#/) for detailed description of the prediction methods. **(b-c)** Selected examples of the best scored putative structures from RaptorX modelling for **(b)** AVRPM3$^{A2/F2}$ and 9 out of 39 members of the AVRPM3$^{A2/F2}$ family, and **(c)** AVRPM3$^{B2/C2}$ and 4 out of 10 members of the AVRPM3B2/C2 family. Full list of predicted structures can be found in Supplementary Data 5.

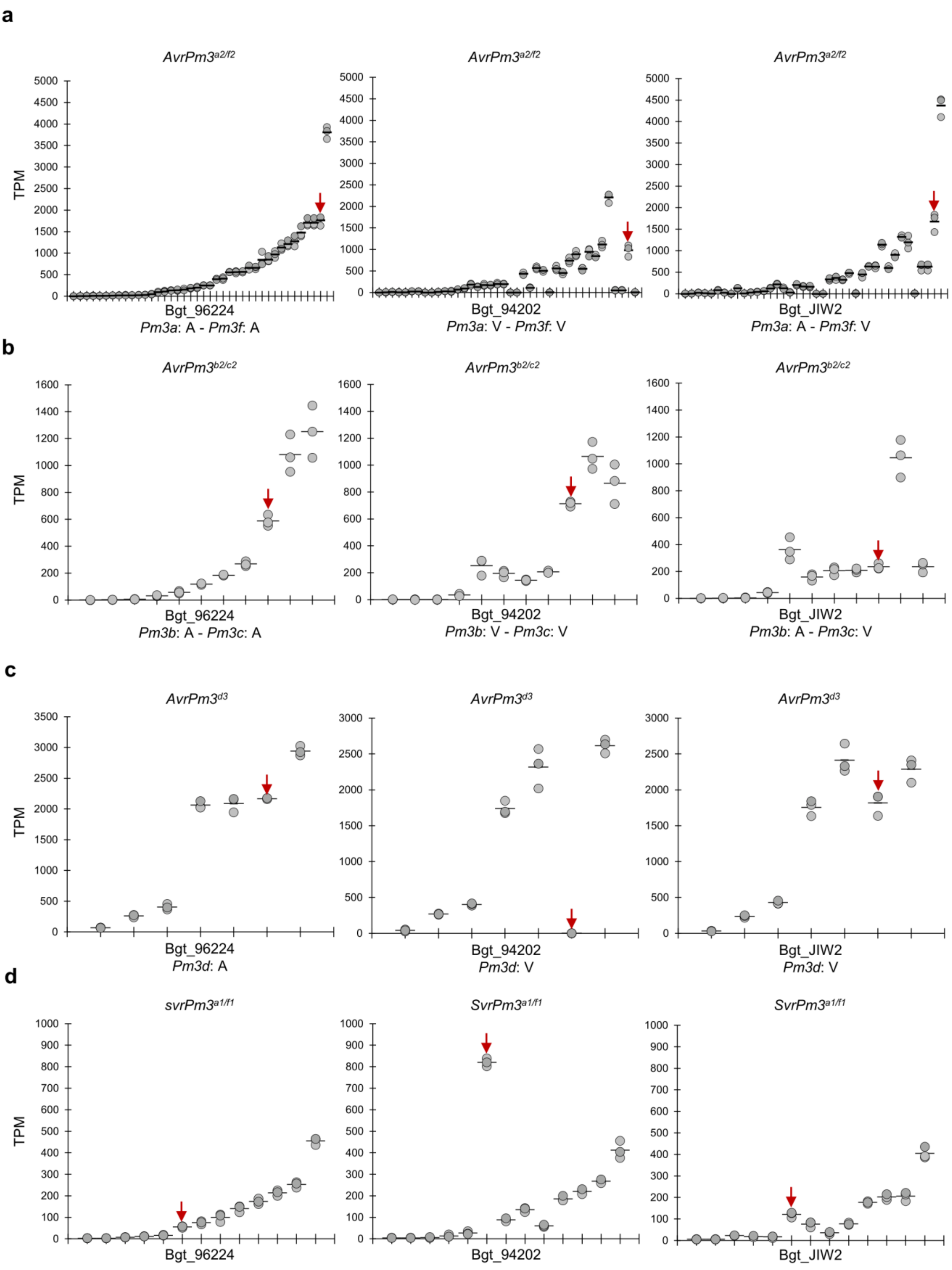
a
AvrPm3a2/f2
TPM
Bgt_96224
Pm3a: A - Pm3f: A
Bgt_94202
Pm3a: V - Pm3f: V
Bgt_JIW2
Pm3a: A - Pm3f: V
b
AvrPm3b2/c2
Pm3b: A - Pm3c: A
Pm3b: V - Pm3c: V
Pm3b: A - Pm3c: V
c
AvrPm3d3
Pm3d: A
Pm3d: V
Pm3d: V
d
svrPm3a1/f1
SvrPm3a1/f1

Supplementary Figure 15. Gene expression analysis of the *AvrPm3*$^{a2/f2}$, *AvrPm3*$^{b2/c2}$, *AvrPm3*^{d3}, and *SvrPm3*$^{a1/f1}$ effector gene families.

RNA-Seq assessment of gene expression levels of the *AvrPm3*$^{a2/f2}$ **(a)**, *AvrPm3*$^{b2/c2}$ **(b)**, *AvrPm3*^{d3} **(c)**, and *SvrPm3*$^{a1/f1}$**(d)** effector gene families in the reference *B.g. tritici* isolates Bgt_96224, _94202, and _JIW2 (avirulence (A) and virulence (V) patterns on relevant *Pm3* alleles are given). Every family member is represented by three vertically aligned dots corresponding to data points originating from three biological replicates. All members are always depicted in the same order in the three isolates. The position of the *AvrPm3*$^{a2/f2}$, *AvrPm3*$^{b2/c2}$, *AvrPm3*^{d3}, and *SvrPm3*$^{a1/f1}$ gene within the plot is indicated with a red arrow. Gene expression levels are indicated as Transcripts Per Kilobase Million (TPM). The mean TPM expression values are indicated with a horizontal line. Raw data underlying the reported gene expression levels are provided in a Source Data File.

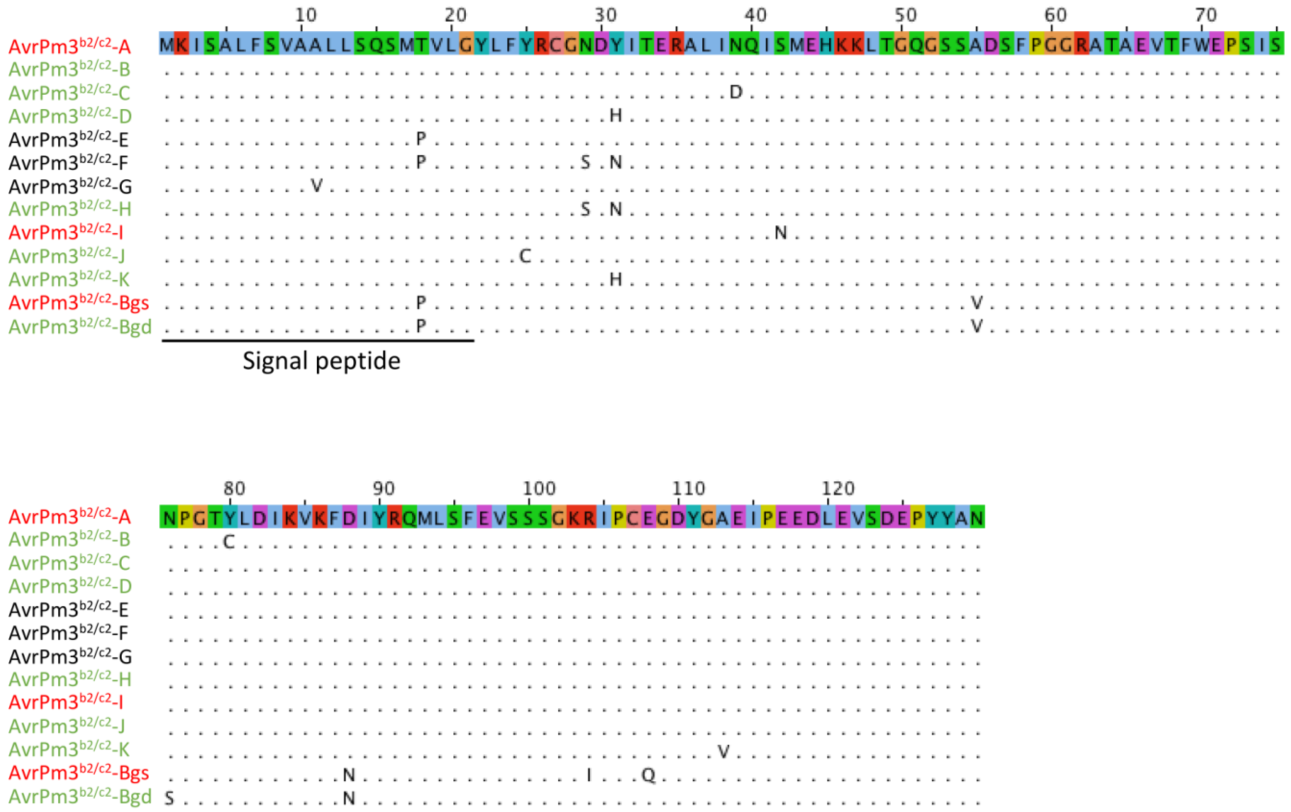

Supplementary Figure 16. Sequence alignments of the natural AVRPM3$^{B2/C2}$ protein variants. Variants recognized by PM3B, variants not-recognized by PM3B and untested variants are marked in red, green and black, respectively. The sequences of AVRPM3$^{B2/C2}$ -A from Bgt_96224 and Bgt_94202 are shown as a reference, for all variants only polymorphic residues are indicated whereas identical residues are represented by dots. The extent of the N-terminal predicted signal peptide is highlighted by a black line. Coloring of the amino acids is based on biochemical or physical properties (ClustalX color scheme: blue: hydrophobic; red: positively charged; purple: negatively charged; green: polar uncharged; yellow: proline; pink: cysteine; orange: glycine).

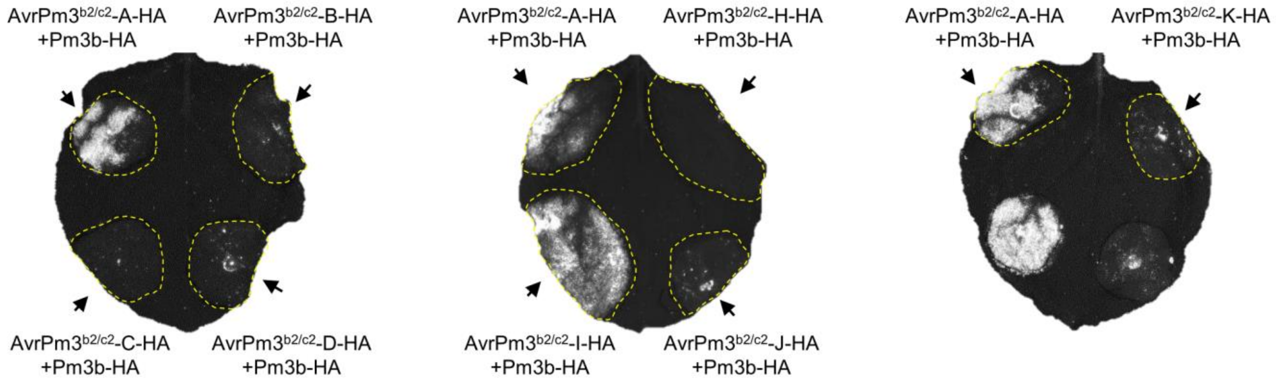

Supplementary Figure 17. Assessment of the functionality of tagged AVRPM3$^{B2/C2}$ haplotypes

Transient co-expression of C terminal HA tag fusion of *AvrPm3*$^{b2/c2}$ haplotypes together with *Pm3b*-HA in *Nicotiana benthamiana* leaves. Combination of *AvrPm3*$^{b2/c2}$-A-HA and *Pm3b*-HA is used as a control. HR was scored using HSR imaging 5 days after leaf agro-infiltration. Results are consistent over at least two independent replicates each consisting of at least 6-8 independent leaves.

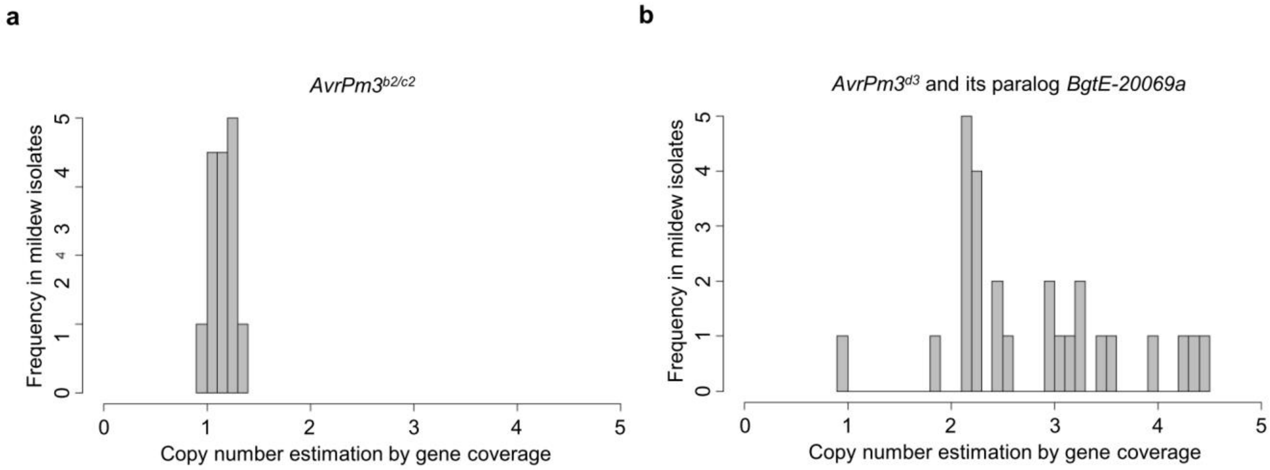

Supplementary Figure 18. Copy number variation of *AvrPm3*$^{b2/c2}$, *AvrPm3*^{d3}, and *BgtE-20069a* in wheat powdery mildew isolates.

Copy number variation (CNV) of **(a)** *AvrPm3*$^{b2/c2}$, and **(b)** *AvrPm3*^{d3} and its orthologue *BgtE-20069a*, in 36 wheat powdery mildew isolates. CNV estimates were extracted from previous analysis by Muller et al. (2018)[2] where all wheat powdery mildew effector gene families were analyzed, and the copy number of every family number was estimated. Raw data underlying copy number estimation is provided in a Source Data File.

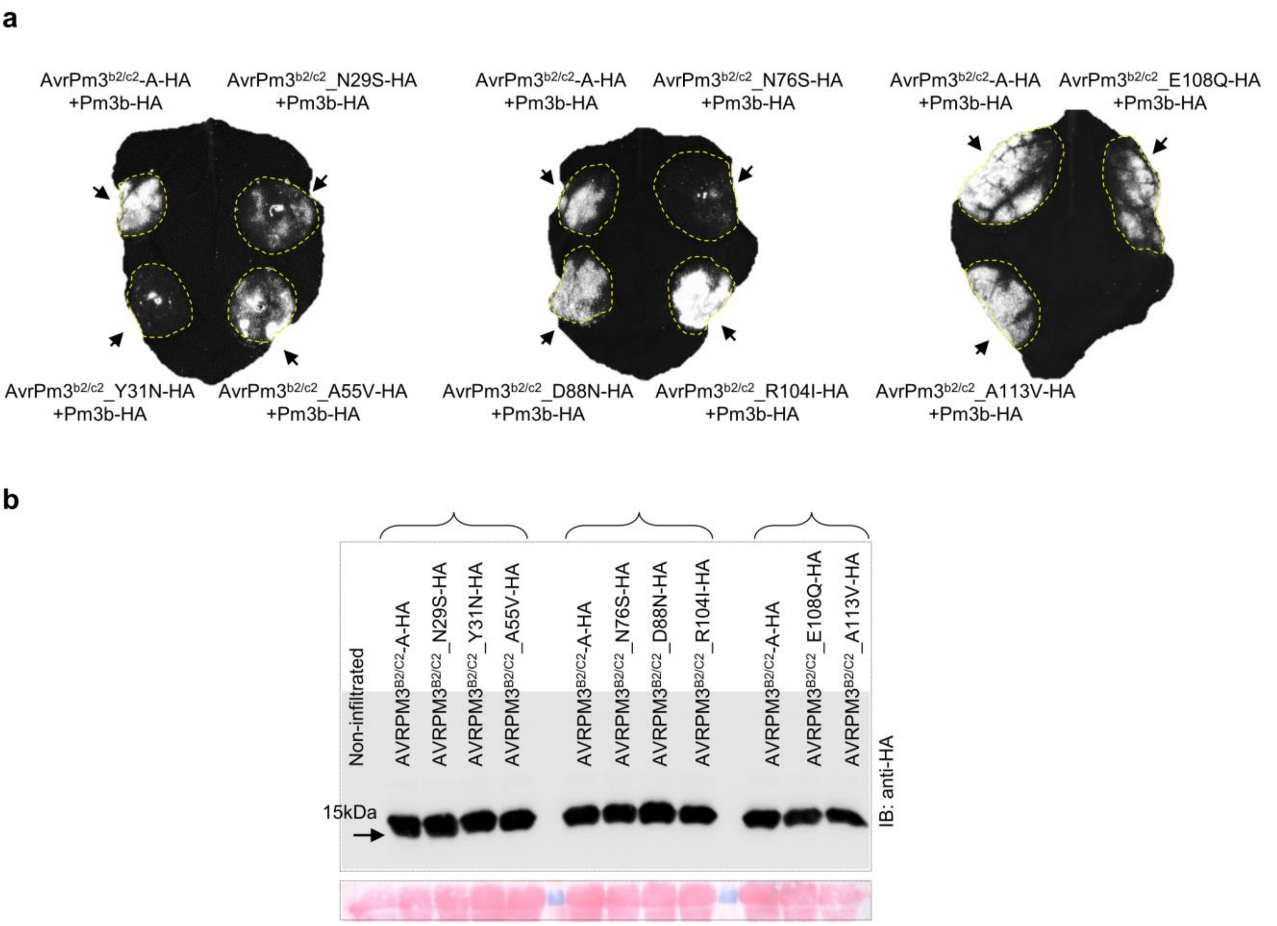

Supplementary Figure 19. Assessment of the functionality and protein expression levels of tagged AVRPM3$^{B2/C2}$ point mutants.

(a) Transient co-expression of C terminal HA tag fusion of *AvrPm3*$^{b2/c2}$ point mutants together with *Pm3b*-HA in *Nicotiana benthamiana* leaves. Combination of *AvrPm3*$^{b2/c2}$-A-HA and *Pm3b*-HA is used as a control. HR was scored using HSR imaging 5 days after leaf agro-infiltration. Results are consistent over at least two independent replicates each consisting of at least 6-8 independent leaves. **(b)** Western blot detection (upper panel) of C terminal HA epitope fusion of AVRPM3$^{B2/C2}$ point mutants. Ponceau staining of the western blot membranes is depicted in the lower panel. Braces indicate samples where all constructs were combined on the same leaf and rotated together with AVRPM3$^{B2/C2}$-A-HA as a reference control. Uncropped Western blot images are provided in a Source Data File.

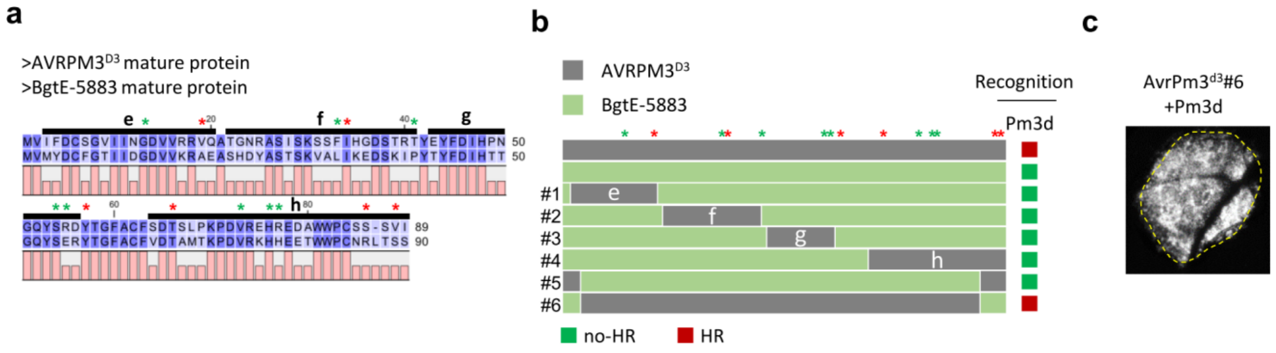

Supplementary Figure 20. Consequence of synthetic domain swaps on the recognition of AVRPM3^{D3} **(a)** Protein sequence alignment of the mature peptide of AVRPM3^{D3} and the closest family member BgtE-5883. **(b)** Schematic representation of the protein domains swapped between AVRPM3^{D3} (grey) and BgtE-5883 (green). Swapped domains are indicated and labeled e, f, g, and h. **(a-b)** Position of the residues identified from the natural sequence diversity (Fig. 4e) are indicated with asterisks. The impact of individual residues on AVR recognition is indicated with 'green' for mutations with a disruptive effect, and 'red' for mutations, with a neutral effect, according to the results summarized in Fig. 4e. **(c)** Transient expression assays in *N. benthamiana* indicating recognition of the AVRPM3^{D3} swap #6 by *Pm3d*. Complete *N. benthamiana* leaf pictures are provided in a Source Data File.

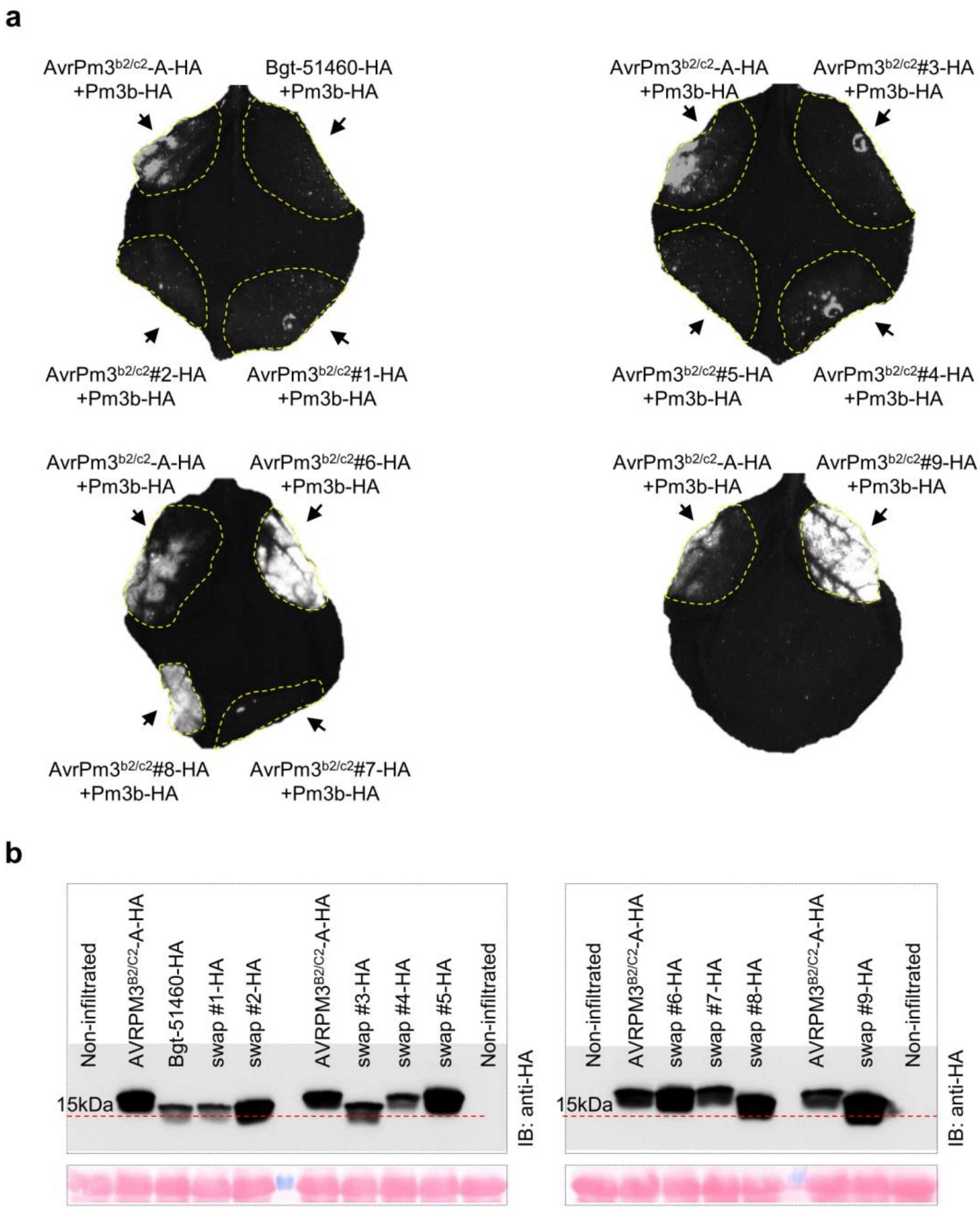

Supplementary Figure 21. Assessment of the functionality and protein expression levels of epitope tagged AVRPM3$^{B2/C2}$ x BGT-51460 domain swaps.

(a) Transient co-expression of C terminal HA tag fusion of *Bgt-51460* and *AvrPm3*$^{b2/c2}$ swaps #1 - #9 together with Pm3b-HA in *Nicotiana benthamiana* leaves. Combination of *AvrPm3*$^{b2/c2}$-A and *Pm3b*-HA is used as a control. HR was scored using HSR imaging 5 days after leaf agro-infiltration. Results are consistent over at least two independent replicates of at least 6-8 independent leaves. **(b)** Western blot detection (upper panel) of C terminal HA epitope fusion of AVRPM3$^{B2/C2}$, the closet family member BGT-51460, and swaps #1 to #9 depicted in Fig. 5b. Ponceau staining of the western blot membranes is depicted in the lower panel. Uncropped Western blot images are provided in a Source Data File.

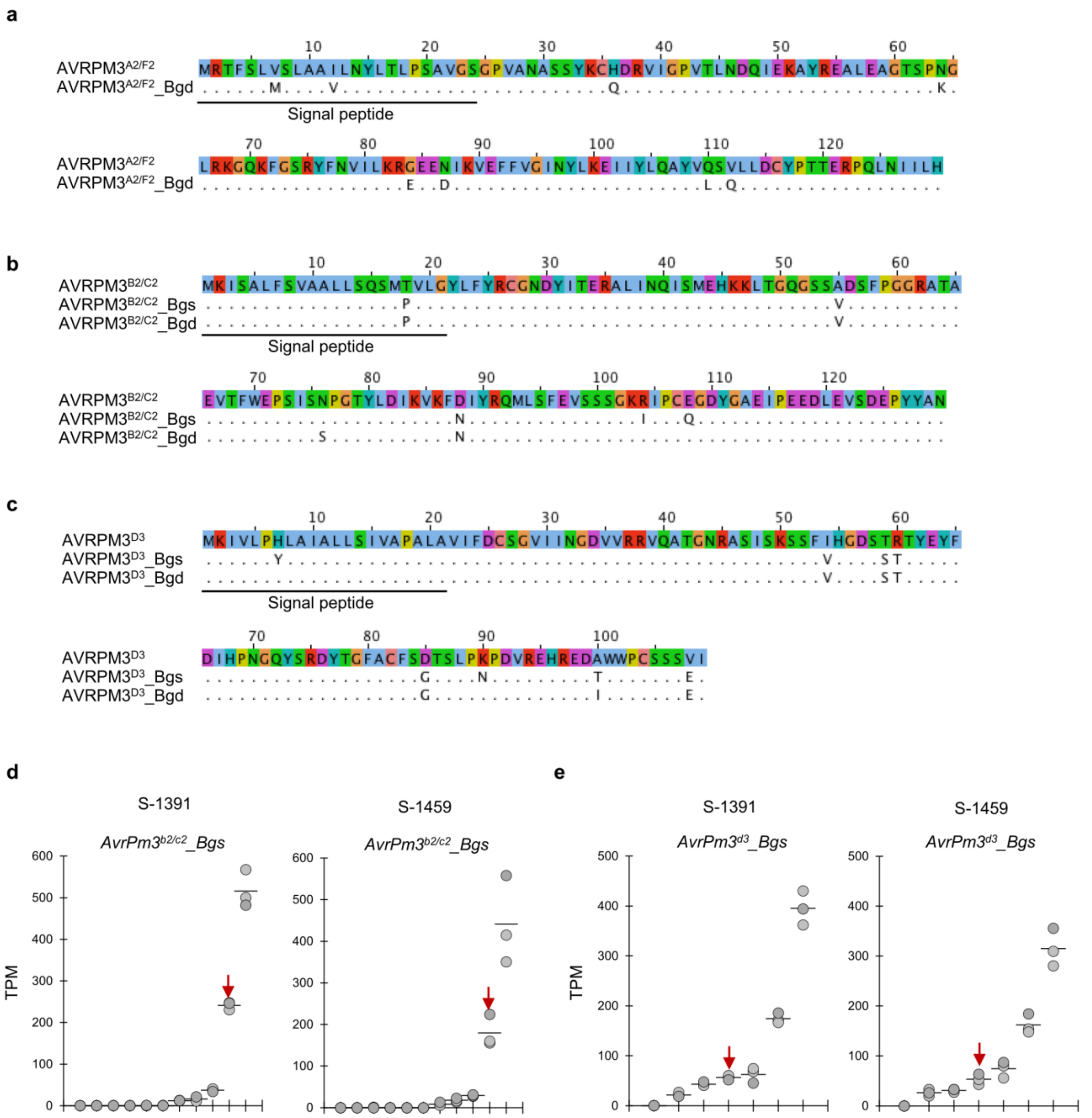

Supplementary Figure 22. Sequence and gene expression analysis of direct *AvrPm3* homologues in rye and *Dactylis* powdery mildews.

(a-c) Protein sequence alignment of **(a)** AVRPM3$^{A2/F2}$, **(b)** AVRPM3$^{B2/C2}$, and **(c)** AVRPM3^{D3} and homologues found in rye and *Dactylis* powdery mildew. The sequence of the AVRPM3 proteins from Bgt_96224 is shown as a reference. For all homologues only polymorphic residues are indicated whereas identical residues are represented by dots. The extent of the N-terminal predicted signal peptide is highlighted by a black line. Coloring of the amino acids is based on biochemical or physical properties (ClustalX color scheme: blue: hydrophobic; red: positively charged; purple: negatively charged; green: polar uncharged; yellow: proline; pink: cysteine; orange: glycine). **(d-e)** RNA-sequencing assessment of gene expression levels of **(d)** *AvrPm3$^{b2/c2}$* and **(e)** *AvrPm3^{d3}* homologues in the *B. g. secalis* isolates S-1391 and S-1459 [4,5]. Every family

member is represented by three vertically aligned dots corresponding to data points originating from three biological replicates. All members are always depicted in the same order in the two isolates. The position of the *AvrPm3*$^{b2/c2}$, and *AvrPm3*^{d3} genes within the plot is indicated with a red arrow. Gene expression levels are indicated as Transcripts Per Kilobase Million (TPM). The mean TPM expression values are indicated with a horizontal line. Raw data underlying the reported gene expression levels are provided in a Source Data File.

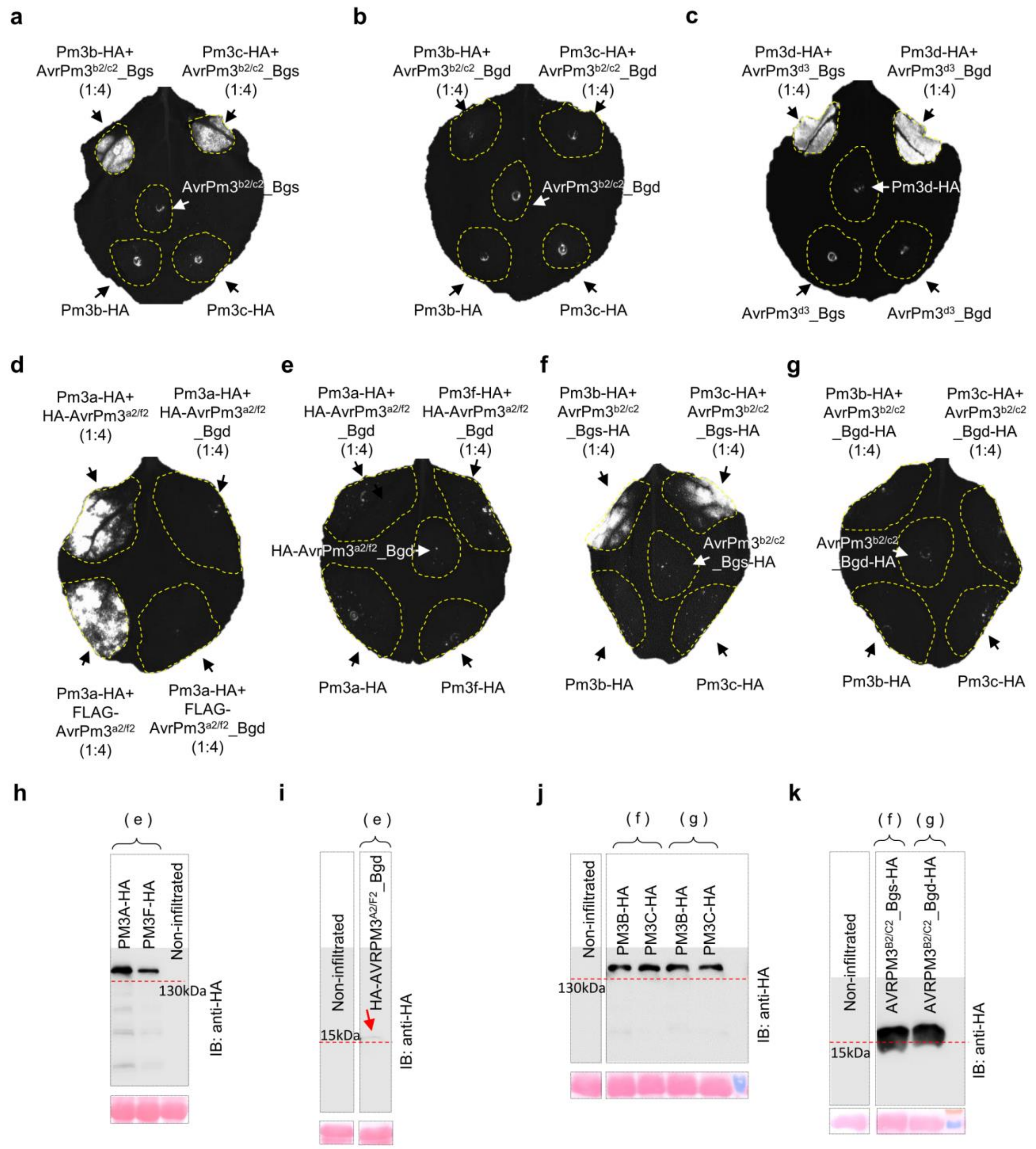

Supplementary Figure 23. *AvrPm3*$^{a2/f2}$*_Bgd*, *AvrPm3*$^{b2/c2}$*_Bgs*, and *AvrPm3*$^{b2/c2}$*_Bgd* functional validation, epitope tagging, and protein expression controls.

(a-c) Transient expression controls for functional validation assays of *AvrPm3*$^{b2/c2}$*_Bgs* **(a)**, *AvrPm3*$^{b2/c2}$*_Bgd* **(b)**, *AvrPm3*^{d3}*_Bgs* and *AvrPm3*^{d3}*_Bgd* **(c)**. **(d)** Functional assessment of *AvrPm3*$^{a2/f2}$*_Bgd* N terminal HA and FLAG epitope fusions in transient co-expression assays together with *Pm3a*-HA. *AvrPm3*$^{a2/f2}$*_Bgd* is not recognized, independently of the epitope tag. **(e)** Functional assessment of HA-*AvrPm3*$^{a2/f2}$*_Bgd* in co-expression assays together with *Pm3a*-HA and *Pm3f*-HA. HA-*AvrPm3*$^{a2/f2}$*_Bgd* is not recognized by *Pm3a*-

HA nor *Pm3f*-HA. **(f-g)** Functional assessment of *AvrPm3*$^{b2/c2}$*_Bgs*-HA **(f)** and *AvrPm3*$^{b2/c2}$*_Bgd*-HA **(g)** epitope tagged constructs in transient co-expression assays together with *Pm3b*-HA and *Pm3c*-HA. HR was revealed by HSR imaging and results were consistent across at least two independent assays of at least 8 leaf replicates. **(h-i)** Protein expression controls for PM3A-HA and PM3F-HA **(h)**, and HA-AVRPM3$^{A2/F2}$_Bgd **(i)** from the same setup depicted in (e). Protein expression controls for PM3B-HA and PM3C-HA **(j)**, and AVRPM3$^{B2/C2}$_Bgs-HA and AVRPM3$^{B2/C2}$_Bgd-HA **(k)** from the same setup depicted in (f) and (g), respectively. Uncropped Western blot images are provided in a Source Data File.

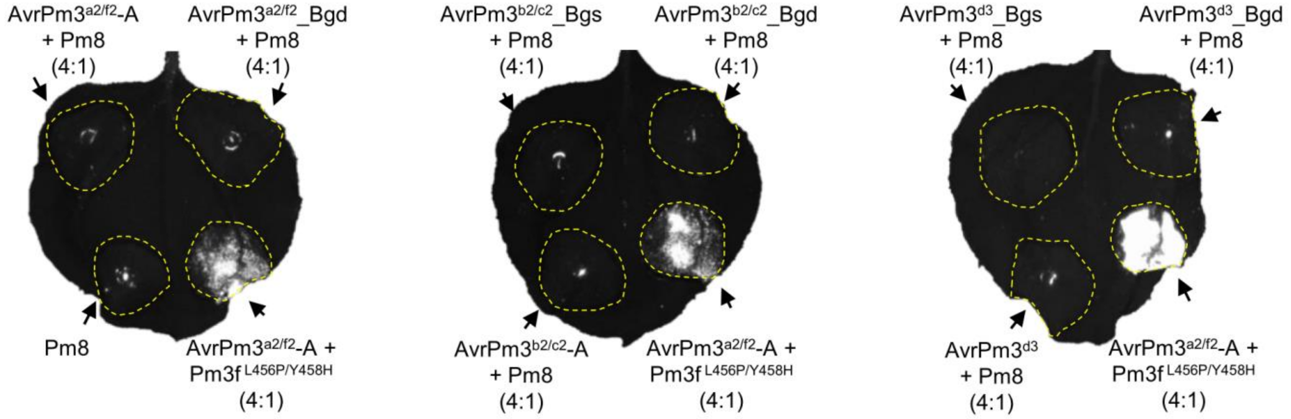

Supplementary Figure 24. Assessment of possible recognition of the *AvrPm3* homologues from rye and *Dactylis* powdery by the *Pm8* resistance gene, a *Pm3* orthologue from rye.

HR is revealed by HSR imaging 5 days post *Nicotiana benthamiana* agroinfiltration. Co-expression of *AvrPm3*$^{a2/f2}$-A and *Pm3f*$^{L456P/Y458H}$ is used as control for proper HR induction. Avr:R ratios are indicated. Results are consistent over at least two assays each consisting of 6–8 independent leaf replicates.

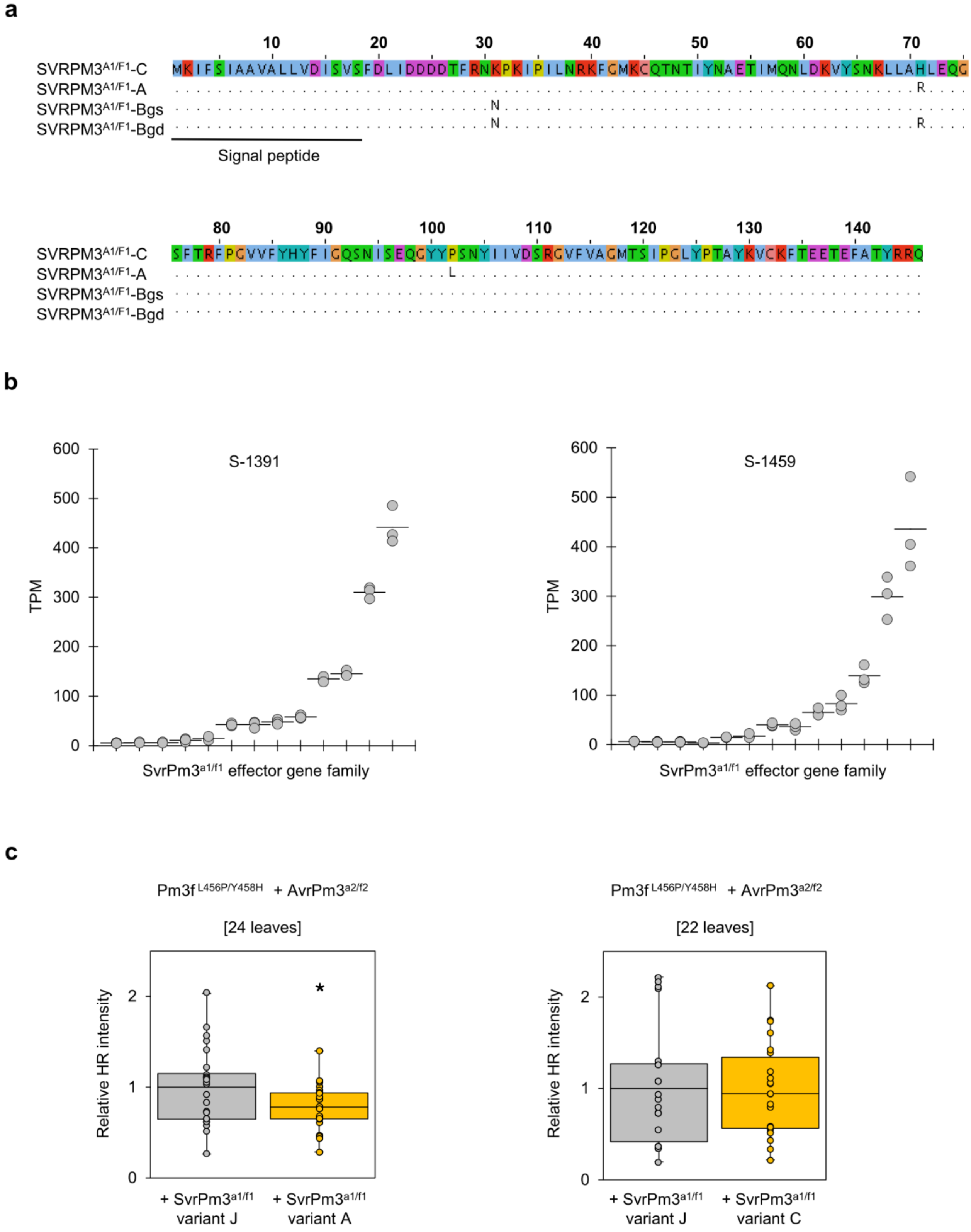
a
10
20
30
40
50
60
70
SVRPM3A1/F1-C
MKIFSIAAVALLVDISVSFDLIDDDDTFRNKPKIPILNRKFGMKCQTNTIYNAETIMQNLDKVYSNKLLAHLEQG
SVRPM3A1/F1-A
R
SVRPM3A1/F1-Bgs
N
SVRPM3A1/F1-Bgd
N
R
Signal peptide
80
90
100
110
120
130
140
SFTRFPGVVFYHYFIGQSNISEQGYYPSNYIIVDSRGVFVAGMTSIPGLYPTAYKVCKFTEETEFATYRRQ
L
b
S-1391
S-1459
TPM
0
100
200
300
400
500
600
SvrPm3a1/f1 effector gene family
c
Pm3f L456P/Y458H + AvrPm3a2/f2
[24 leaves]
[22 leaves]
Relative HR intensity
0
1
2
*
+ SvrPm3a1/f1 variant J
+ SvrPm3a1/f1 variant A
+ SvrPm3a1/f1 variant C

Supplementary Figure 25. Characterization of the *SvrPm3*$^{a1/f1}$*-J* variant from rye powdery mildew. **(a)** Sequence alignments of SVRPM3$^{A1/F1}$ protein variants. The sequence of SVRPM3$^{A1/F1}$-C from Bgt_96224 (according to McNally et al. 2018[6]) is shown as a reference. For the active suppressor SVRPM3$^{A1/F1}$ variant A from Bgt_94202 as well as the homologous proteins from *B.g. secalis* (identical to variant J from McNally et al. 2018[6]), and *B.g. dactylidis*, only polymorphic residues are indicated whereas identical residues are represented by dots. The extent of the N-terminal predicted signal peptide is indicated by a black line. Coloring of the amino acids is based on biochemical or physical properties (ClustalX color scheme: blue: hydrophobic; red: positively charged; purple: negatively charged; green: polar uncharged; yellow: proline; pink: cysteine; orange: glycine). **(b)** RNA-sequencing assessment of gene expression levels of the *SvrPm3*$^{a1/f1}$ family in the rye powdery mildew isolates S-1391 and S-1459 [4,5]. Every family member is represented by three vertically aligned dots corresponding to data points originating from three biological replicates. All members are always depicted in the same order in the two isolates. The position of *SvrPm3*$^{a1/f1}$*-J* within the plot is indicated with a red arrow. Gene expression levels are indicated as Transcripts Per Kilobase Million (TPM). The mean TPM expression values are indicated with a horizontal line. Raw data underlying the reported gene expression levels are provided in a Source Data File. **(c)** Quantification of the HR response in presence of *SvrPm3*$^{a1/f1}$*-J* (the variant encoded in *B.g. secalis*), compared to the active suppressor *SvrPm3*$^{a1/f1}$-A (left panel), and the inactive suppressor *SvrPm3*$^{a1/f1}$-C (right panel). Results demonstrate that *B.g. secalis* encodes an inactive suppressor comparable to the previously characterized *SvrPm3*$^{a1/f1}$-C from wheat powdery mildew. The number or independent leaf replicates is indicated. Mean values are indicated by the middle line in the boxplot. Individual data points are plotted along the whiskers delineating minimum and maximum values. Statistical significance was assessed with a two-sided Student t.Test for paired data and indicated with (*; $p < 0.05$). Raw data underlying the reported averages are provided in a Source Data File.

Supplementary Table 1. Primers used for the amplification of the *AvrPm3*$^{b2/c2}$ (*BgtE-20002*) and *AvrPm3*^{d3} (*BgtE-20069b*) haplotype from natural isolates of powdery mildew.

**Primer/Identifier**	**Sequence (5'-3')**
BgtE-20002-F	AAGCCGTGAGATCCAAGCTA
BgtE-20002-R	GTCATTAAGTCATTAGGCCATTCGAC
BgtE-20069a/b-F	CGTCATTTGAAGAGACTTGTG
BgtE-20069a/b-R	CTAATCTCGACAACTCTGTTATCG

Supplementary Table 2. Primers used for site-directed mutagenesis to produce *BgtE-20069a_96224*, *AvrPm3*^{d3} (*BgtE-20069b*), *SvrPm3*$^{a1/f1}$*_94202* and epitope tagged *AvrPm3* and *SvrPm3* variants

Primer/Identifier	Sequence (5’-3’)
BgtE-20069_F53S_F	GTAAATCCAGCTCTATACACGGAGATTC
BgtE-20069_F53S_R	TTATACTGGCTCGGTTACCCGT
BgtE-20069_I86T_F	TTCTGACACAAGCTTGCCAA
BgtE-20069_I86T_R	AAACAAGCAAAGCCCGTATAGTC
SvrPm3a1/f1_H54R_F	GGTCCTTTACCCGATTC
SvrPm3a1/f1_H54R_R	CCTGCTCTAGTCTAGCAAGAAG
SvrPm3a1/f1_P85L_F	ATCATCGTGGACTCTCG
SvrPm3a1/f1_P85L_R	GTAATTGGAAAGGTAGTATCCCTG
AvrPm3a2/f2-HA_F	GTTCCAGATTACGCTTAAAAGGGTGGGCGC
AvrPm3a2/f2-HA_R	ATCGTATGGGTAGTGCAAGATAATGTTCAACTGTG
AvrPm3a2/f2-FLAG_F	GATGACGACAAGTAAAAGGGTGGGCGC
AvrPm3a2/f2-FLAG_R	GTCTTTGTAGTCGTGCAAGATAATGTTCAACTGTG
HA-AvrPm3a2/f2_F	GTTCCAGATTACGCTGGTCCTGTCGCAAATGCTA
HA-AvrPm3a2/f2_R	ATCGTATGGGTACATGGTGAAGGGGGC
FLAG-AvrPm3a2/f2_F	GATGACGACAAGGGTCCTGTCGCAAATGCTA
FLAG-AvrPm3a2/f2_R	GTCTTTGTAGTCCATGGTGAAGGGGGC
AvrPm3b2/c2-HA_F	GTTCCAGATTACGCTTAGAAGGGTGGGCGC
AvrPm3b2/c2-HA_R	ATCGTATGGGTAGTTAGCATAATAAGGCTCGTCTG
AvrPm3b2/c2-FLAG_F	GATGACGACAAGTAGAAGGGTGGGCGC
AvrPm3b2/c2-FLAG_R	GTCTTTGTAGTCGTTAGCATAATAAGGCTCGTCTG
HA-AvrPm3b2/c2_F	GTTCCAGATTACGCTTATTTGTTTTACCGATGCGG
HA-AvrPm3b2/c2_R	ATCGTATGGGTACATGGTGAAGGGGGC
FLAG-AvrPm3b2/c2_F	GATGACGACAAGTATTTGTTTTACCGATGCGG
FLAG-AvrPm3b2/c2_R	GTCTTTGTAGTCCATGGTGAAGGGGGC
AvrPm3d3-HA_F	GTTCCAGATTACGCTTGAAAGGGTGGGCGC
AvrPm3d3-HA_R	ATCGTATGGGTAGATGACACTTGAACTGCACG
AvrPm3d3-FLAG_F	GATGACGACAAGTGAAAGGGTGGGCGC
AvrPm3d3-FLAG_R	GTCTTTGTAGTCGATGACACTTGAACTGCACG
HA-AvrPm3d3_F	GTTCCAGATTACGCTGTGATCTTCGATTGCTCAGG
HA-AvrPm3d3_R	ATCGTATGGGTACATGGTGAAGGGGGC
FLAG-AvrPm3d3_F	GATGACGACAAGGTGATCTTCGATTGCTCAGG
FLAG-AvrPm3d3_R	GTCTTTGTAGTCCATGGTGAAGGGGGC
SvrPm3a1/f1-HA_F	GTTCCAGATTACGCTTAGAAGGGTGGGCGC
SvrPm3a1/f1-HA_R	ATCGTATGGGTACTGCCGCCTATAGGTTGC
SvrPm3a1/f1-FLAG_F	GATGACGACAAGTAGAAGGGTGGGCGC
SvrPm3a1/f1-FLAG_R	GTCTTTGTAGTCCTGCCGCCTATAGGTTGC
HA-SvrPm3a1/f1_F	GTTCCAGATTACGCTTTCGACCTCATTGACGATG
HA-SvrPm3a1/f1_R	ATCGTATGGGTACATGGTGAAGGGGGC
FLAG-SvrPm3a1/f1_F	GATGACGACAAGTTCGACCTCATTGACGATG
FLAG-SvrPm3a1/f1_R	GTCTTTGTAGTCCATGGTGAAGGGGGC

Supplementary Table 3. Primers used to amplify genetic marker regions in *Locus_3*.

Primer/Identifier	Sequence (5’-3’)
Ctg118_21_F	TCCACTTCACCGAATACGTGATCT
Ctg118_21_R	CAAGGCAATCGCTACCACTACT
Sc667_3_F	GCATCGCTTCTTGTACACTTGTATTT
Sc667_3_R	AACAGATACTAGAAATCGCAATCG
Ctg118_18_F	ACCGGGATTGAATGTTCCTG
Ctg118_18_R	TTGAGCCTGCTGTTGGACAT
Ctg118_14_F	GACAGCTGGTTTCCCTGTCC
Ctg118_14_R	GTGGTTACGGCCCACCTTTA
Ctg49_2_F	GGATGGTGTGGTTGGCTATC
Ctg49_2_R	GGCAGAGGACGAGAGTGAGA

Supplementary Note 1. Effector benchmarking procedure.

The effector benchmarking approach was developed to reduce the large number of known candidate effector genes (595 at the time of analysis) to a manageable number for experimental analysis. It is based on the hypothesis that effector genes encoding for avirulence proteins are likely to share similar features in terms of sequence properties and gene expression levels. We therefore implemented a benchmarking scheme to identify powdery mildew effectors that resemble the functionally validated avirulence genes *AvrPm3*$^{a2/f2}$, *AvrPm2* from *B.g. tritici* [1,7] and *Avr*$_{a1}$, *Avr*$_{a13}$ from *B.g. hordei* [8].

We classified the features defining a putative candidate *Avr* effector into four groups: "1.1 Sequence polymorphism" between the Bgt_96224 reference isolate (avirulent on all *Pm3* alleles) and the isolates Bgt_94202 (virulent on all *Pm3* alleles) and Bgt_JIW2 (virulent on *Pm3c* and *Pm3f* only), "1.2 Protein structure", "2.1 Absolute expression" in the reference isolate Bgt_96224, and 2.2 "Differential expression" between Bgt_96224 and the phenotypically contrasting isolates Bgt_94202 and Bgt_JIW2 (Supplementary Data 1). For each category, we defined a scoring scheme based on the assumption that Bgt_96224, which is avirulent on all the *Pm3* alleles, should encode for all *AvrPm3* specificities. Therefore, putative effectors best fulfilling the criteria for an *Avr* in the Bgt_96224 isolate can be considered as possible *AvrPm3* genes. For each of the described 4 categories, we defined a series of criteria each of which describes specific features of an expected *Avr*. For example, in the category "1.2 Protein structure" we defined 10 (code 121-130 in Supplementary Data 1) criteria assessing the features of each mildew effector in terms of presence of a signal peptide, the number of cysteines, and the size of the native peptide (i.e. including the signal peptide) (Supplementary Data 1). Each criterion was given a weight so that for example a putative effector encoding a protein within the size range of the previously identified AVRs (defined as 115-135 aa) would receive a higher score than those encoding for much bigger or much small peptides (e.g. < 70 aa, or >300 aa). To determine the appropriate weight each criterion should receive, we manually tested several scoring schemes and progressively adapted the weights so that the functionally validated *Avrs* would score among the top 20 best candidates (i.e. serving as positive control for proper *Avr* identification). After each round we assessed to what extent the subset of the top 100 candidates was enriched in effectors simultaneously combining the best criteria for protein size, expression level, and sequence polymorphism as depicted in Supplementary Figure 1. The whole benchmarking procedure was executed based on the powdery mildew genome annotation and RNA-Seq data described in previous work by Praz and colleagues [5].

All candidates were manually re-annotated, and a subset of 16 effectors was applied to molecular validation of mRNA structure by RACE-PCR (Supplementary Data 4). We excluded members of the *AvrPm3*$^{a2/f2}$ family that had been already tested at the time we designed the assay [1,6]. Subsequently, the top 100 candidates were codon optimized for expression in *N. benthamiana*, and cloned by gene synthesis (Supplementary Data 3). This approach led to the identification *AvrPm3*$^{b2/c2}$, and *AvrPm3*^{d3} thus demonstrating that effector

benchmarking is indeed a rapid and effective alternative to *Avr* identification by classical map-based cloning or GWAS. However, while effector benchmarking demonstrates several advantages compared to GWAS and map-based cloning, one major limitation is that it can only be used if the candidate genes have well defined features. Also, the effectiveness of effector benchmarking is highly dependent on the quality of the genome annotation, since it is based on the comparison of well annotated effectors. We therefore propose that this approach is complementary to- and builds on classical genetics approaches, and it can be adapted to other plant pathogenic fungi based on specific features of avirulence genes in those systems.

Supplementary Note 2. Annotation of the *AvrPm3*$^{b2/c2}$ genetic locus.

The position of the *AvrPm3*^{b2} GWAS peak was located within the genetic interval previously identified as the genetic *Locus_3* which controls specificity towards *Pm3b* and *Pm3c* [1]. In an initial effort to map the *AvrPm3c* gene in a genetic cross between the mildew isolates Bgt_96224 and Bgt_JIW2 segregating for *Pm3c* [9], two flanking markers M049LE and ctg118_21, were identified (Supplementary Figure 2). Here we took advantage of the Bacterial Artificial Chromosome (BAC) clone library which was assembled for the reference isolates Bgt_96224 as another source for uncovering the full sequence of *Locus_3* [10]. The BAC clones were previously assembled into Finger Printed Contigs (FPC) thus allowing the identification of 6 overlapping BAC clones covering the physical region defined by *Locus_3* (Supplementary Figure 2). We used the same approach previously described by Bourras and colleagues [1] to validate the physical overlap between the BACs which has resulted in the selection of five clones (7i16, 28j03, 7p01, 29k04, and 4k17) for sequencing (Supplementary Figure 2)

We combined different resources to thoroughly annotate this genetically complex locus as follows: (i) we used the high quality PacBio sequence annotation of the locus, derived from the reference isolate Bgt_96224 [11], (ii) we assembled the sequences of the 5 BAC clones from the same Bgt_96224 reference isolate, spanning the whole region covering the flanking genetic markers (Supplementary Figure 2), (iii) we used RNA-Seq data from the Bgt_96224 reference (avirulent on *Pm3*b and *Pm3c*) to manually curate and thoroughly annotate genes and transposable elements, and (iv) we used RNAseq and genome re-sequencing data from the Bgt_94202 isolate (virulent on *Pm3b* and *Pm3c*) to identify sequence polymorphisms, locus rearrangements, and differential expression patterns that can be associated with the phenotype. This has resulted in a very-high quality sequence annotation of *Locus_3*, including the identification of novel effector sequences.

Supplementary Note 3. Epitope tagging of AVR and SVR proteins.

HA and FLAG epitope tags were added N and C-terminally to the mature peptide encoded by $AvrPm3^{a2/f2}$, $AvrPm3^{b2/c2}$, $AvrPm3^{d3}$ and $SvrPm3^{a1/f1}$ using site-directed mutagenesis (SDM). All constructs were recombined into the pIPKb004 expression vector and mobilized by electroporation into the *Agrobacterium tumefaciens* strain GV3101 as previously described [1,12]. Protein detection assays upon transient expression in *N. benthamiana* (see methods), revealed all 4 effectors had significantly different levels of tag tolerance including: not detectable ($\mathrm{AVRPM3}^{\mathrm{D3}}$), detectable in N terminal fusions only ($\mathrm{AVRPM3}^{\mathrm{A2/F2}}$), detectable in C terminal fusions only ($\mathrm{AVRPM3}^{\mathrm{B2/C2}}$) (Fig. 2a-c), and detectable in N and C terminal fusions ($\mathrm{SVRPM3}^{\mathrm{A1/F1}}$) (Fig. 3d). For $\mathrm{AVRPM3}^{\mathrm{D3}}$ no protein could be detected independently of tag position or sequence, despite several attempts optimizing western blotting procedure, using different ODs of *Agrobacteria* (0.5-1.5) and different time-points of extraction (1-4dpi). Altogether, this data suggest that epitope fusions can have severe negative effects on AVRPM3 protein expression and stability, suggesting these effectors are highly sensitive to structural modifications. Finally, $\mathrm{SVRPM3}^{\mathrm{A1/F1}}$ stands out in these assays as all attempted fusions resulted in the detection of high amounts of protein. This would suggest that $\mathrm{SVRPM3}^{\mathrm{A1/F1}}$ is a structurally more stable effector protein as compared to the AVRPM3s.

Supplementary Note 4. Specificity of AVR recognition and NLR-NLR interactions among the *Pm3* alleles. Compared to other well-described allelic series of resistance genes such as *RPP13*, the *L* and the *Mla* series from Arabidopsis, flax and barley, respectively, the *Pm3* alleles stand out with their high level of similarity (>97%) on the protein level [13–16]. An extreme case is exemplified by PM3D and PM3E that only differ by two amino acids in the LRR domain but recognize distinctly different spectra of mildew races [17]. Furthermore, PM3D and PM3E only differ from the PM3CS susceptible allele by respectively 3 and 2 residues in the LRR domain, yet they are among the strongest alleles in the field [17,18]. Interestingly, neither AVRPM3^{D3}, nor its recognized homologues from *B.g. secalis* or *B.g. dactylidis* are recognized by PM3E or PM3CS. Similarly, in the fungus, the duplicated paralog of *AvrPm3*^{d3} (*BgtE-20069a*) found in the genome of Bgt_96224 and Bgt_94202, encodes a protein that only differs from the active AVR by 2 and 3 amino acid polymorphisms, respectively. Taken together these observations indicate that specificity of AVR recognition by the PM3 variants is highly sensitive to single amino acid changes on both sides of the interaction.

Evidence of inter-allelic suppression among the Pm3 variants was initially reported from several genetic crosses between near-isogenic *Pm3* lines [12]. In one case this observation was molecularly validated in transient co-expression assays in *N. benthamiana* [1,12] where it was shown that *Pm3b* was able to suppress the HR induced by an auto-active variant of *Pm3f* [12], and also the HR induced by the natural *Pm3a* and *Pm3f* alleles upon recognition of *AvrPm3*$^{a2/f2}$ [1]. Here, we tested the suppression spectra of all *Pm3* alleles and the *Pm3CS* ancestral sequence, in presence of the newly identified *AvrPm3* genes (*AvrPm3*$^{b2/c2}$, and *AvrPm3*^{d3}), and taking full advantage of our improved experimental setup (i.e. use of codon optimized *Avr* constructs, and HR visualization using the HSR imaging technology).

We assayed the suppression activity for *Pm3a*, *Pm3b*, *Pm3c*, *Pm3d*, *Pm3e*, *Pm3f* and *Pm3CS*, in transient co-expression assays when every allele was combined with *AvrPm3*$^{a2/f2}$-*Pm3a*, *AvrPm3*$^{a2/f2}$-*Pm3f*, *AvrPm3*$^{b2/c2}$-*Pm3b*, *AvrPm3*$^{b2/c2}$-*Pm3c*, and *AvrPm3*^{d3}-*Pm3d*. The resulting HR was compared to a control where the putative NLR suppressor was replaced by GUS at equal ratios, similar to the experimental set-up previously described by Bourras and colleagues [1]. We found that *Pm3a* and *Pm3f* had no suppression activity towards *Pm3b*, *Pm3c*, or *Pm3d* (Supplementary Figure 8). The *Pm3b/c* alleles had the broadest suppression spectrum and were able to suppress recognition of *AvrPm3*$^{a2/f2}$ and *AvrPm3*^{d3} by *Pm3a/f* and *Pm3d*, respectively (Supplementary Figure 9a-b). *Pm3d* was able to suppress recognition of *AvrPm3*$^{b2/c2}$ by *Pm3b/c*, but interestingly *Pm3d* only suppressed the recognition of *AvrPm3*$^{a2/f2}$ by the weaker *Pm3f* allele (Supplementary Figure 9c). Similarly, *Pm3e* was able to suppress *AvrPm3*$^{a2/f2}$ and *AvrPm3*$^{b2/c2}$ only when combined with the weaker *Pm3f* and *Pm3c* alleles, respectively (Supplementary Figure 9d). We also assayed the inter-allelic suppression spectrum of the *Pm3CS* ancestral susceptible allele, using the same NLR-NLR suppression assay. We found that *Pm3CS* was capable to suppress *Pm3a*, *Pm3f*, and *Pm3b* (Supplementary Figure 9e), but not *Pm3c* and *Pm3d*. Interestingly PM3C and PM3D are identical to PM3CS in the CC-NB-ARC domain (CC:

coiled-coil, NB: nucleotide-binding, ARC: APAF-1 “apoptotic protease-activating factor-1”, R proteins and CED-4 “Caenorhabditis elegans death-4 protein”), while all three NLRs suppressed by PM3CS (i.e. PM3A, PM3F, and PM3B) have divergent CC-NB-ARC sequences (Figure 6a-c, lower panel).

To summarize, these assays show that *Pm3a/f* have no NLR suppression activity and are mostly suppressed by the other *Pm3* alleles. *Pm3e* is only active on the weaker *Pm3c* and *Pm3f* alleles, while *Pm3b/c* and *Pm3d* have reciprocal suppression capacity and act as the strongest suppressors among the functional *Pm3* NLRs. These results indicate that NLR mediated suppression of the *AvrPm3*-*Pm3* interactions is *Pm3* allele specific and independent of the cognate *Avr* sequence, and we hypothesize this can be mediated by the formation of NLR heterodimers or multimers inhibiting *R* gene activation or proper AVR recognition.

Supplementary Note 5. Gene expression analysis of the *AvrPm3*$^{a2/f2}$, *AvrPm3*$^{b2/c2}$, *AvrPm3*^{d3}, and *SvrPm3*$^{a1/f1}$ effector gene families.

We took advantage of the availability of the highly improved wheat powdery mildew genome sequence [11], with an updated definition of the effector gene families, to assess relative gene expression levels of the three *AvrPm3* genes and the *SvrPm3*$^{a1/f1}$ suppressor within their respective gene families. We used the RNA-Seq data previously produced by Praz and colleagues [5], the new mildew PacBio derived mildew genome [11], and the RNA-Seq analysis software “Salmon” [19], to assess for gene expression levels at 2dpi in the three reference isolates Bgt_96224, _94202, and _JIW2.

We found that the active *AvrPm3*$^{a2/f2}$ allele encoded by Bgt_96224, and _JIW2 (Supplementary Figure 15a), the active *AvrPm3*$^{b2/c2}$ allele encoded by all three isolates (Supplementary Figure 15b), as well as the active *AvrPm3*^{d3} allele encoded by Bgt_96224 and _JIW2 (Supplementary Figure 15c), were always among the most highly expressed members of their effector gene families. The data also suggests there is an association between the expression levels of the active *SvrPm3*$^{a1/f1}$ suppressor encoded by Bgt_94202 and _JIW2 (Supplementary Figure 15d), and virulence of these two isolates on *Pm3b/c* and *Pm3d*. In particular, low expression levels of the active *SvrPm3*$^{a1/f1}$ suppressor in _JIW2 seem to be sufficient for suppressing *Avr* recognition by the weak *Pm3f* and *Pm3c* alleles but not by the strong *Pm3a* and *Pm3b* alleles or *Pm3d*. These results suggest that the *AvrPm3* effectors are important virulence factors and that *SvrPm3*$^{a1/f1}$ might play an important role in maintaining effector function while suppressing effector recognition.

Supplementary Note 6. Consequence of synthetic domain swaps on the recognition of AVRPM3$^{B2/C2}$ and AVRPM3^{D3}.

Based on the functional data from the genetic diversity screens suggesting the presence of specific domains involved in R protein recognition, we wanted to study a possible structural basis for the specificity of the AVRPM3$^{B2/C2}$-PM3B and AVRPM3^{D3}-PM3D interactions. Therefore, we designed domain swaps between AVRPM3$^{B2/C2}$ and AVRPM3^{D3} and the most closely related member of their effector families, BGT-51460 and BGTE-5883, respectively. We did so by exchanging regions of approximately 20 amino acids, flanked by conserved residues, between the AVR and its non-recognized partner (Fig. 5a, Supplementary Figure 21a). Care was taken that regions with high levels of natural diversity (Figure 5b, Supplementary Figure 21b) would be located entirely within one exchanged segment. We postulate that structural conservation among close family members will allow the replacement of protein subdomains while preserving AVR protein structure. This should reveal which parts of the protein are involved in recognition.

We designed eight swaps between AVRPM3$^{B2/C2}$ and BGT-51460 all of which were codon optimized for *N. benthamiana*, cloned without signal peptide by gene synthesis and tested for recognition by PM3B and PM3C. In constructs #1-4 we replaced a defined region of BGT-51460 by its counterpart from AVRPM3$^{B2/C2}$ whereas for constructs #5-8 the opposite strategy was used (Fig. 5b). Interestingly none of the defined regions of AVRPM3$^{B2/C2}$ introduced into BGT-51460 conferred recognition by PM3B or PM3C on its own (swaps#1-4, Fig. 5b). Consistent with these findings, the two regions (segments a and c) harboring naturally occurring SNPs with a disruptive effect on *R*-gene recognition could not be replaced by BGT-51460 sequences without loss of recognition (swap#5 & #7, Fig. 5b). In contrast, one region of AVRPM3$^{B2/C2}$ (segment 'b') with low genetic diversity among natural isolates could be replaced without negative impact on *R*-gene recognition, and resulted in a stronger HR response (swap#6, Fig. 5c). Similarly, replacement of segment 'd' from AVRPM3$^{B2/C2}$ by its counterpart from BGT-51460 in swap#8 resulted in significantly stronger *Avr* recognition by *Pm3b* and *Pm3c* (swap#8, Fig. 5d). Finally, while individual replacement of segments 'a' and 'c' from BGT-51460 with their counterpart from AVRPM3$^{B2/C2}$ had no impact on recognition (swap#1 and #3), a stronger HR was observed with swap#9 where these segments were simultaneously exchanged (Fig. 5e). This data demonstrates that regions 'a' and 'c', are necessary and sufficient to confer AVR function, and together with regions 'b' and 'd'they can additionally affect the strength of NLR-AVR recognition. Taken together these findings imply that AVRPM3$^{B2/C2}$ recognition is dependent on two regions that correspond to sequences previously defined by the natural diversity screens, plus two regions possibly corresponding to a structurally conserved region in the AVRPM3$^{B2/C2}$ family.

For AVRPM3^{D3} and its paralog BGTE-20069A we observed a more uniform distribution of disruptive SNPs in natural diversity screens covering the complete central part of the AVR protein between the Y/FxC motif

and the conserved C-terminal cysteine (Figure 4c). We designed four swaps between AVRPM3^{D3} and BGTE-5883 covering different stretches of polymorphic residues (#1-4 Supplementary Figure 21a-b) and two additional ones in which only N- and C- terminal ends of AVRPM3^{D3}, consisting of two and six polymorphic residues, were replaced by sequences from BGTE-5883 and vice versa (#5-6 Supplementary Figure 21a-b). Similar to the findings from AVRPM3$^{B2/C2}$ none of the defined subdomains of AVRPM3^{D3} was sufficient to confer recognition by PM3D on its own (#1-5, Supplementary Figure 21b), whereas construct #6, containing the complete central part of AVRPM3^{D3} resulted in strong HR when combined with the R-protein, indicating that recognition is independent of the C- and N-terminal regions of the AVR (Supplementary Figure 21c)

To summarize, these experiments indicate structural conservation between AVRPM3$^{B2/C2}$ and the closest effector family member since several subdomains can be readily exchanged without loss of recognition. Furthermore, our data implies that multiple protein surface regions are involved in the interaction with the corresponding R-proteins and that information from natural diversity screens can be used to define such regions. This further supports the hypothesis that overall protein structure as well as specific contact regions are important for recognition.

Supplementary Note 7. Evidence for the role of *Avr-R* interactions as determinants of host-specificity in cereal mildews.

The role of *Avr-R* interactions in host specificity was originally investigated using genetic crosses between cereal mildews in a seminal work published over 20 years ago by Matsumura and Tosa [23]. Based on genetic analysis of phenotypic segregation patterns in a cross between two isolates of wheat and rye powdery mildew, the authors provided genetic evidence that the rye mildew isolate Sk-1 carries *AvrPm1, AvrPm2, AvrPm3a, AvrPm3b, AvrPm3c, and AvrPm4a*. In a recent study by Praz and colleagues [7] a functional *AvrPm*2 from rye mildew was indeed isolated and functionally validated for recognition by the *Pm2* resistance gene. In another study by Praz and colleagues [5], a comparative transcriptomic approach was used to study gene expression patterns in triticale powdery mildew, a new *forma specialis* that is a hybrid of wheat and rye mildews [4]. The authors suggested that altered expression of multiple effector genes, in particular *Avr* and *Svr* related factors, might play a role in mildew host adaptation based on hybridization [5].

In this study we had a unique opportunity to probe Matsumura and Tosa's predictions at the molecular level in the *Pm3* allelic series of NLRs. We aimed at investigating the role of the *Pm3* alleles as a determinant of host specificity to *B.g. secalis* and *B.g. dactylidis*, two mildew *formae speciales* specifically growing on rye (*Secale cereale*) and *Dactylis glomerata*. Rye is a wide spread cereal crop and a member of the *Triticeae* tribe, while Dactylis is a wild grass and a distant *Poeae* relative from the *Dactylidinae* tribe [24,25]. *B.g. secalis* has diverged from *B.g. tritici* within the *tritici* clade, while *B.g. dactylis* has very likely emerged after a host jump from the *tritici* clade to *Dactylis* [4]. On the host side, the functional diversity of *Pm3* alleles has emerged after the formation of hexaploid wheat, and all alleles have very likely diversified from *Pm3CS*, a non-functional ancestral sequence [26]. In this context, it is likely that the *Pm3* alleles have co-evolved along host specialization of *B.g. tritici* to the newly evolved hexaploid bread wheat to recognize important effectors for mildew virulence. In such case, conservation of the *Avr* function of these effectors in other *formae speciales* would demonstrate that the *Pm3* alleles can also act as a barrier to non-adapted mildew forms. Based on the conservation of *Avr* function of these effectors from other *formae speciales* we therefore hypothesized that the *Pm3* alleles, can also act as host-specificity determinants against non-adapted mildew forms, in addition to their role in race-specific resistance to adapted wheat mildew isotes.

To test this hypothesis, we made use of two sets of *Pm3* wheat lines. First, *Pm3b* and *Pm3d* transgenic lines in the background of the 'Bobwhite' cultivar, previously shown to confer strong race-specific resistance towards *B.g. tritici* both under laboratory conditions and in the field [27,28]. Second, the near-isogenic wheat lines 'Chul' backcrossed 8 times in Chancellor (Chul^{8xCC}, a *Pm3b* NIL), and Sonora^{8xCC} (a *Pm3c* NIL) that have been used to identify the genetic loci associated with avirulence towards *Pm3b* and *Pm3c* in *B.g. tritici* [1,9] (this study). We challenged the *Pm3* wheat lines and corresponding susceptible controls 'Bobwhite' and 'Chancellor' with

two different *B.g. secalis* isolates, S-1391 and S-1459, for which we have RNA-seq data showing that the *AvrPm3*$^{b2/c2}$ and *AvrPm3*^{d3} homologues encoded in these non-adapted pathogens are expressed (Supplementary Figure 26b). The ability of the rye isolates to infect the non-host wheat was assessed microscopically, at an early stage of the infection (48 hours), where compatible isolates can usually form a haustorium and a few secondary hyphae (hereafter referred to as "microcolony"). We used two different staining methods (see Methods) to distinguish the following phenotypic categories: (i) microcolony formation in the absence of hypersensitive cell-death, indicating successful host-penetration at an early stages of infection reminiscent of an infection from an adapted mildew, and (ii) arrest of spore growth in the presence or absence of a detectable hypersensitive cell-death, reminiscent of a race-specific resistance response. In agreement with our hypothesis that *Pm3b*, *Pm3c* and *Pm3d* contribute to non-host resistance to non-adapted *formae speciales*, the rate of microcolony formation of both tested *B.g. secalis* isolates was significantly ($p < 0.05$), and consistently reduced on the transgenic and near-isogenic *Pm3* wheat lines when compared to the susceptible controls 'Bobwhite' and 'Chancellor' (Figure 6d-e). We conclude that these assays further demonstrate that the *Pm3* alleles are potent host-specificity determinants, as predicted by Matsumura and Tosa two decades ago[23].

References

1. Bourras, S. *et al.* Multiple Avirulence Loci and Allele-Specific Effector Recognition Control the Pm3 Race-Specific Resistance of Wheat to Powdery Mildew. *Plant Cell* **27,** 2991–3012 (2015).
2. Müller, M. C. *et al.* A chromosome-scale genome assembly reveals a highly dynamic effector repertoire of wheat powdery mildew. *New Phytol.* (2018). doi:DOI:10.1111/nph.15529
3. Zimmermann, L. *et al.* A Completely Reimplemented MPI Bioinformatics Toolkit with a New HHpred Server at its Core. *J. Mol. Biol.* (2018). doi:10.1016/j.jmb.2017.12.007
4. Menardo, F. *et al.* Hybridization of powdery mildew strains gives rise to pathogens on novel agricultural crop species. *Nat. Genet.* **48,** 201–205 (2016).
5. Praz, C. R. *et al.* Non-parent of Origin Expression of Numerous Effector Genes Indicates a Role of Gene Regulation in Host Adaption of the Hybrid Triticale Powdery Mildew Pathogen. *Front. Plant Sci.* **9,** 49 (2018).
6. McNally, K. E. *et al.* Distinct domains of the AVRPM3A2/F2 avirulence protein from wheat powdery mildew are involved in immune receptor recognition and putative effector function. *New Phytol.* (2018). doi:10.1111/nph.15026
7. Praz, C. R. *et al.* AvrPm2 encodes an RNase-like avirulence effector which is conserved in the two different specialized forms of wheat and rye powdery mildew fungus. *New Phytol.* **213,** 1301–1314 (2017).
8. Lu, X. *et al.* Allelic barley MLA immune receptors recognize sequence-unrelated avirulence effectors of the powdery mildew pathogen. *Proc. Natl. Acad. Sci.* **113,** E6486–E6495 (2016).
9. Parlange, F. *et al.* Genetic and molecular characterization of a locus involved in avirulence of Blumeria graminis f. sp. tritici on wheat Pm3 resistance alleles. *Fungal Genet. Biol.* **82,** 181–92 (2015).
10. Wicker, T. *et al.* The wheat powdery mildew genome shows the unique evolution of an obligate biotroph. *Nat. Genet.* **45,** 1092–1096 (2013).
11. Müller, M. C. *et al.* A chromosome-scale assembly of the wheat powdery mildew genome provides insight into origin and evolution of genes associated with host-pathogen interactions. *Submitted* (2018).
12. Stirnweis, D. *et al.* Suppression among alleles encoding nucleotide-binding-leucine-rich repeat resistance proteins interferes with resistance in F1hybrid and allele-pyramided wheat plants. *Plant J.* **79,** 893–903 (2014).
13. Rose, L. E. *et al.* The Maintenance of Extreme Amino Acid Diversity at the Disease Resistance Gene, RPP13, in Arabidopsis thaliana. *Genetics* **166,** 1517–1527 (2004).
14. Ravensdale, M., Nemri, A., Thrall, P. H., Ellis, J. G. & Dodds, P. N. Co-evolutionary interactions between host resistance and pathogen effector genes in flax rust disease. *Molecular Plant Pathology* **12,** 93–102 (2011).

15. Seeholzer, S. *et al.* Diversity at the Mla powdery mildew resistance locus from cultivated barley reveals sites of positive selection. *Mol. Plant-Microbe Interact.* **23,** 497–509 (2010).
16. Bhullar, N. K., Mackay, M. & Keller, B. Genetic diversity of the Pm3 powdery mildew resistance alleles in wheat gene bank accessions as assessed by molecular markers. *Diversity* **2,** 768–786 (2010).
17. Brunner, S. *et al.* Intragenic allele pyramiding combines different specificities of wheat Pm3 resistance alleles. *Plant J.* **64,** 433–445 (2010).
18. Koller, T., Brunner, S., Herren, G., Hurni, S. & Keller, B. Pyramiding of transgenic Pm3 alleles in wheat results in improved powdery mildew resistance in the field. *Theor. Appl. Genet.* **131,** 861–871 (2018).
19. Patro, R., Duggal, G., Love, M. I., Irizarry, R. A. & Kingsford, C. Salmon provides fast and bias-aware quantification of transcript expression. *Nat. Methods* **14,** 417–419 (2017).
20. Thomas, W. J., Thireault, C. A., Kimbrel, J. A. & Chang, J. H. Recombineering and stable integration of the Pseudomonas syringae pv. syringae 61 hrp/hrc cluster into the genome of the soil bacterium Pseudomonas fluorescens Pf0-1. *Plant J.* **60,** 919–928 (2009).
21. Kim, M. G., Geng, X., Lee, S. Y. & Mackey, D. The Pseudomonas syringae type III effector AvrRpm1 induces significant defenses by activating the Arabidopsis nucleotide-binding leucine-rich repeat protein RPS2. *Plant J.* (2009). doi:10.1111/j.1365-313X.2008.03716.x
22. Liu, C. *et al.* The stripe rust fungal effector PEC6 suppresses pattern-triggered immunity in a host species-independent manner and interacts with adenosine kinases. *New Phytologist* (2016). doi:10.1111/nph.14034
23. Matsumura, K. & Tosa, Y. The Rye Mildew Fungus Carries Avirulence Genes Corresponding to Wheat Genes for Resistance to Races of the Wheat Mildew Fungus. *Phytopathology* **85,** 753 (1995).
24. Soreng, R. J. *et al.* A worldwide phylogenetic classification of the Poaceae (Gramineae); A worldwide phylogenetic classification of the Poaceae (Gramineae). *J. Syst. Evol.* **53,** 117–137 (2015).
25. Soreng, R. J. *et al.* A worldwide phylogenetic classification of the Poaceae (Gramineae) II: An update and a comparison of two 2015 classifications. *Journal of Systematics and Evolution* **55,** 259–290 (2017).
26. Krattinger, S. G. & Keller, B. Molecular genetics and evolution of disease resistance in cereals. *New Phytol.* **212,** 320–332 (2016).
27. Brunner, S. *et al.* Transgenic Pm3b wheat lines show resistance to powdery mildew in the field. *Plant Biotechnol. J.* (2011). doi:10.1111/j.1467-7652.2011.00603.x
28. Brunner, S. *et al.* Transgenic Pm3 multilines of wheat show increased powdery mildew resistance in the field. *Plant Biotechnol. J.* (2012). doi:10.1111/j.1467-7652.2011.00670.x
