# Supplementary material for: The AvrPm3-Pm3 effector-NLR interactions control both race-specific resistance and host-specificity of cereal mildews on wheat
Source: Nat Commun. 2019 May 23;10:2292. doi: 10.1038/s41467-019-10274-1 (PMC6533294; doi:10.1038/s41467-019-10274-1)
Supplement: Supplementary file 2 — Description of Additional Supplementary Files [file 41467_2019_10274_MOESM2_ESM.pdf]

Description of Additional Supplementary Files

File Name: Supplementary Data 1
Description: Effector benchmarking scoring sheet. Detailed description and source data of the effector benchmarking scores and scoring scheme. The benchmarking criteria are defined in the Summary table. Two main categories are defined based on (1) effector sequence features at the DNA and protein levels and (2) effector gene expression. Each category is further subdivided into criteria, and the weight of each of the criteria for candidate Avr scoring is indicated. The benchmarking scores for every effector are defined in the Scoring table. In addition to the defined criteria, the total score and the final ranking of the candidates is indicated. All categories and scores can be searched using table filter option in Excel. Detailed benchmarking procedure is provided in Supplementary Note 1. Effector gene sequences, family classification, and RNA-Seq based gene expression data was obtained from Praz et al. (2017) [https://dx.doi.org/10.3389%2Ffpls.2018.00049].

File Name: Supplementary Data 2
Description: List of the B.g. tritici genotypes and phenotypes used for GWAS. Excel table summarizing geographic origin, classification, year of collection and virulence phenotype (1=virulent, 0=avirulent) on the wheat line Chul/8*Chancellor (Pm3b) for each isolate used for GWAS.

File Name: Supplementary Data 3
Description: Sequences produced by gene-synthesis. Excel table listing all constructs that were codon optimized for N. benthamiana and produced by genesynthesis. The exact sequences (5'-3') and our commercial gene synthesis suppliers are indicated.

File Name: Supplementary Data 4
Description: Primers used for RACE-PCR based annotation of selected effector benchmarking candidates. Excel table listing primers used for RACE-PCR based annotation of effector benchmarking candidates. To achieve specific amplification of effector genes, primary and secondary (nested) primers were used. Gene name, RACE type and primer sequence (5'-3') are indicated.

File Name: Supplementary Data 5
Description: In silico structural modelling of secondary and tertiary folds of the AVRPM3 proteins. Detailed results from RaptorX structural modeling of all members of the AVRPM3A2/F2 (Sheet 1), AVRPM3B2/C2 (Sheet 2), and AVRPM3D3 (Sheet 3) effector families. For each protein, up to five alternative models were predicted and all are listed with their respective rank assigned by RaptorX based on p-value for similarity and the quality of the modeling. Detailed explanation of RaptorX output format and data explanation can be found at [http://raptorx.uchicago.edu/documentation/]
